# Supplementary material for: Species Diversity and Distribution Characteristics of Calonectria in Five Soil Layers in a Eucalyptus Plantation
Source: J Fungi (Basel). 2021 Oct 13;7(10):857. doi: 10.3390/jof7100857 (PMC8541508; doi:10.3390/jof7100857)
Supplement: Supplementary file 1 [file jof-07-00857-s001.zip › jof-1381697-supplementary.pdf]

Journal of Fungi

Supplementary Materials:

Table S1. Number of sampling points containing each *Calonectria* species in each soil layer.

Soil layer	*C. hongkongensis*	*C. aconidialis*	*C. kyotensis*	*C. ilicicola*	*C. chinensis*	*C. orientalis*	All six *Calonectria* species
0–20 cm	78	44	16	8	1	0	92
20–40 cm	31	10	4	4	0	0	40
40–60 cm	14	3	2	1	0	1	20
60–80 cm	2	2	1	1	0	1	7
80–100 cm	4	2	0	0	0	0	5
All five soil layers	82	49	22	11	1	1	93

Journal of Fungi

MDPI

Table S2. All 1037 isolates obtained and sequenced in this study.

Sampling point No. [1]	Soil layer	Sample and isolate information [2]	Isolate No. [3]	Identity	Genotype [4]	Collectors	GenBank Accession No. [5]			
							tef1	*tub2*	*cmdA*	*his3*
1	0–20 cm	20200709-1-(1)_0–20 cm_A_R1_SC1	CSF20257	*C. aconidialis*	ACAA	S.F. Chen, L.L. Liu, J.L. Han, Y Liu & X.Y. Liang	OK167865	OK168902	OK169156	OK169240
1	0–20 cm	20200709-1-(1)_0–20 cm_A_R1_SC2	CSF20258	*C. hongkongensis*	AAAA	S.F. Chen, L.L. Liu, J.L. Han, Y Liu & X.Y. Liang	OK167035	OK168072	OK169109	OK169194
1	0–20 cm	20200709-1-(1)_0–20 cm_A_R2_SC1	CSF20259	*C. hongkongensis*	AFAA	S.F. Chen, L.L. Liu, J.L. Han, Y Liu & X.Y. Liang	OK167610	OK168647	OK169124	OK169208
1	0–20 cm	20200709-1-(1)_0–20 cm_A_R2_SC2	CSF20260	*C. kyotensis*	ABAB	S.F. Chen, L.L. Liu, J.L. Han, Y Liu & X.Y. Liang	OK167983	OK169020	OK169164	OK169248
1	0–20 cm	20200709-1-(1)_0–20 cm_B_R1_SC1	CSF20261	*C. kyotensis*	AB--	S.F. Chen, L.L. Liu, J.L. Han, Y Liu & X.Y. Liang	OK167984	OK169021	– [6]	–
1	0–20 cm	20200709-1-(1)_0–20 cm_B_R1_SC2	CSF20262	*C. kyotensis*	AB--	S.F. Chen, L.L. Liu, J.L. Han, Y Liu & X.Y. Liang	OK167985	OK169022	–	–
1	20–40 cm	20200709-1-(1)_0–40 cm_A_R1_SC1	CSF20263	*C. hongkongensis*	AA--	S.F. Chen, L.L. Liu, J.L. Han, Y Liu & X.Y. Liang	OK167036	OK168073	–	–
1	20–40 cm	20200709-1-(1)_0–40 cm_A_R1_SC2	CSF20264	*C. hongkongensis*	AA--	S.F. Chen, L.L. Liu, J.L. Han, Y Liu & X.Y. Liang	OK167037	OK168074	–	–
1	20–40 cm	20200709-1-(1)_0–40 cm_A_R1_SC3	CSF20265	*C. hongkongensis*	AA--	S.F. Chen, L.L. Liu, J.L. Han, Y Liu & X.Y. Liang	OK167038	OK168075	–	–
1	20–40 cm	20200709-1-(1)_0–40 cm_A_R1_SC4	CSF20266	*C. hongkongensis*	AA--	S.F. Chen, L.L. Liu, J.L. Han, Y Liu & X.Y. Liang	OK167039	OK168076	–	–
1	40–60 cm	20200709-1-(1)_0–60 cm_C_R1_SC1	CSF20267	*C. hongkongensis*	AA--	S.F. Chen, L.L. Liu, J.L. Han, Y Liu & X.Y. Liang	OK167040	OK168077	–	–
1	40–60 cm	20200709-1-(1)_0–60 cm_C_R1_SC2	CSF20268	*C. hongkongensis*	AA--	S.F. Chen, L.L. Liu, J.L. Han, Y Liu & X.Y. Liang	OK167041	OK168078	–	–
1	40–60 cm	20200709-1-(1)_0–60 cm_C_R1_SC3	CSF20269	*C. hongkongensis*	AA--	S.F. Chen, L.L. Liu, J.L. Han, Y Liu & X.Y. Liang	OK167042	OK168079	–	–
1	40–60 cm	20200709-1-(1)_0–60 cm_C_R1_SC4	CSF20270	*C. hongkongensis*	AA--	S.F. Chen, L.L. Liu, J.L. Han, Y Liu & X.Y. Liang	OK167043	OK168080	–	–

2	0–20 cm	20200709-1-(2)_0–20 cm_A_R1_SC1	CSF20271	*C. hongkongensis*	AAAA	S.F. Chen, L.L. Liu, J.L. Han, Y Liu & X.Y. Liang	OK167044	OK168081	OK169110	OK169195
2	0–20 cm	20200709-1-(2)_0–20 cm_A_R1_SC2	CSF20272	*C. hongkongensis*	AA--	S.F. Chen, L.L. Liu, J.L. Han, Y Liu & X.Y. Liang	OK167045	OK168082	–	–
2	0–20 cm	20200709-1-(2)_0–20 cm_B_R2_SC1	CSF20273	*C. hongkongensis*	AA--	S.F. Chen, L.L. Liu, J.L. Han, Y Liu & X.Y. Liang	OK167046	OK168083	–	–
2	0–20 cm	20200709-1-(2)_0–20 cm_B_R2_SC2	CSF20274	*C. hongkongensis*	AA--	S.F. Chen, L.L. Liu, J.L. Han, Y Liu & X.Y. Liang	OK167047	OK168084	–	–
2	20–40 cm	20200709-1-(2)_0–40 cm_A_R1_SC1	CSF20275	*C. kyotensis*	BAAA	S.F. Chen, L.L. Liu, J.L. Han, Y Liu & X.Y. Liang	OK168002	OK169039	OK169168	OK169252
2	20–40 cm	20200709-1-(2)_0–40 cm_A_R1_SC2	CSF20276	*C. kyotensis*	BAAA	S.F. Chen, L.L. Liu, J.L. Han, Y Liu & X.Y. Liang	OK168003	OK169040	OK169169	OK169253
2	20–40 cm	20200709-1-(2)_0–40 cm_A_R1_SC3	CSF20277	*C. kyotensis*	BA--	S.F. Chen, L.L. Liu, J.L. Han, Y Liu & X.Y. Liang	OK168004	OK169041	–	–
2	20–40 cm	20200709-1-(2)_0–40 cm_A_R1_SC4	CSF20278	*C. kyotensis*	BA--	S.F. Chen, L.L. Liu, J.L. Han, Y Liu & X.Y. Liang	OK168005	OK169042	–	–
2	20–40 cm	20200709-1-(2)_0–40 cm_B_R1_SC1	CSF20279	*C. hongkongensis*	AA--	S.F. Chen, L.L. Liu, J.L. Han, Y Liu & X.Y. Liang	OK167048	OK168085	–	–
2	20–40 cm	20200709-1-(2)_0–40 cm_B_R1_SC2	CSF20280	*C. hongkongensis*	AA--	S.F. Chen, L.L. Liu, J.L. Han, Y Liu & X.Y. Liang	OK167049	OK168086	–	–
2	20–40 cm	20200709-1-(2)_0–40 cm_B_R1_SC3	CSF20281	*C. hongkongensis*	AA--	S.F. Chen, L.L. Liu, J.L. Han, Y Liu & X.Y. Liang	OK167050	OK168087	–	–
2	20–40 cm	20200709-1-(2)_0–40 cm_B_R1_SC4	CSF20282	*C. hongkongensis*	AA--	S.F. Chen, L.L. Liu, J.L. Han, Y Liu & X.Y. Liang	OK167051	OK168088	–	–
2	20–40 cm	20200709-1-(2)_0–40 cm_B_R2_SC1	CSF20283	*C. hongkongensis*	AA--	S.F. Chen, L.L. Liu, J.L. Han, Y Liu & X.Y. Liang	OK167052	OK168089	–	–
2	20–40 cm	20200709-1-(2)_0–40 cm_B_R2_SC2	CSF20284	*C. hongkongensis*	AA--	S.F. Chen, L.L. Liu, J.L. Han, Y Liu & X.Y. Liang	OK167053	OK168090	–	–
2	20–40 cm	20200709-1-(2)_0–40 cm_B_R2_SC3	CSF20285	*C. hongkongensis*	AA--	S.F. Chen, L.L. Liu, J.L. Han, Y Liu & X.Y. Liang	OK167054	OK168091	–	–
2	20–40 cm	20200709-1-(2)_0–40 cm_B_R2_SC4	CSF20286	*C. hongkongensis*	AA--	S.F. Chen, L.L. Liu, J.L. Han, Y Liu & X.Y. Liang	OK167055	OK168092	–	–
3	0–20 cm	20200709-1-(3)_0–20 cm_A_R2_SC1	CSF20291	*C. hongkongensis*	AAAA	S.F. Chen, L.L. Liu, J.L. Han, Y Liu & X.Y. Liang	OK167056	OK168093	OK169111	OK169196

3	0–20 cm	20200709-1-(3)_0–20 cm_A_R2_SC2	CSF20292	*C. hongkongensis*	AA--	S.F. Chen, L.L. Liu, J.L. Han, Y Liu & X.Y. Liang	OK167057	OK168094	–	–
3	80–100 cm	20200709-1-(3)_0–100 cm_A_R1_SC1	CSF20293	*C. hongkongensis*	AA--	S.F. Chen, L.L. Liu, J.L. Han, Y Liu & X.Y. Liang	OK167058	OK168095	–	–
3	80–100 cm	20200709-1-(3)_0–100 cm_A_R1_SC2	CSF20294	*C. hongkongensis*	AA--	S.F. Chen, L.L. Liu, J.L. Han, Y Liu & X.Y. Liang	OK167059	OK168096	–	–
3	80–100 cm	20200709-1-(3)_0–100 cm_A_R1_SC3	CSF20295	*C. hongkongensis*	AA--	S.F. Chen, L.L. Liu, J.L. Han, Y Liu & X.Y. Liang	OK167060	OK168097	–	–
3	80–100 cm	20200709-1-(3)_0–100 cm_A_R1_SC4	CSF20296	*C. hongkongensis*	AA--	S.F. Chen, L.L. Liu, J.L. Han, Y Liu & X.Y. Liang	OK167061	OK168098	–	–
3	80–100 cm	20200709-1-(3)_0–100 cm_D_R1_SC1	CSF20305	*C. hongkongensis*	AA--	S.F. Chen, L.L. Liu, J.L. Han, Y Liu & X.Y. Liang	OK167062	OK168099	–	–
3	80–100 cm	20200709-1-(3)_0–100 cm_D_R1_SC2	CSF20306	*C. hongkongensis*	AA--	S.F. Chen, L.L. Liu, J.L. Han, Y Liu & X.Y. Liang	OK167063	OK168100	–	–
3	80–100 cm	20200709-1-(3)_0–100 cm_D_R1_SC3	CSF20307	*C. hongkongensis*	AA--	S.F. Chen, L.L. Liu, J.L. Han, Y Liu & X.Y. Liang	OK167064	OK168101	–	–
3	80–100 cm	20200709-1-(3)_0–100 cm_D_R1_SC4	CSF20308	*C. hongkongensis*	AA--	S.F. Chen, L.L. Liu, J.L. Han, Y Liu & X.Y. Liang	OK167065	OK168102	–	–
4	0–20 cm	20200711-1-(1)_0–20 cm_A_R1_SC1	CSF20309	*C. hongkongensis*	AFAA	S.F. Chen, L.L. Liu, J.L. Han, Y Liu & X.Y. Liang	OK167611	OK168648	OK169125	OK169209
4	0–20 cm	20200711-1-(1)_0–20 cm_A_R1_SC2	CSF20310	*C. hongkongensis*	AF--	S.F. Chen, L.L. Liu, J.L. Han, Y Liu & X.Y. Liang	OK167612	OK168649	–	–
4	0–20 cm	20200711-1-(1)_0–20 cm_A_R2_SC1	CSF20311	*C. hongkongensis*	AF--	S.F. Chen, L.L. Liu, J.L. Han, Y Liu & X.Y. Liang	OK167613	OK168650	–	–
4	0–20 cm	20200711-1-(1)_0–20 cm_A_R2_SC2	CSF20312	*C. hongkongensis*	AF--	S.F. Chen, L.L. Liu, J.L. Han, Y Liu & X.Y. Liang	OK167614	OK168651	–	–
4	0–20 cm	20200711-1-(1)_0–20 cm_B_R1_SC1	CSF20313	*C. hongkongensis*	AA--	S.F. Chen, L.L. Liu, J.L. Han, Y Liu & X.Y. Liang	OK167066	OK168103	–	–
4	0–20 cm	20200711-1-(1)_0–20 cm_B_R1_SC2	CSF20314	*C. hongkongensis*	AA--	S.F. Chen, L.L. Liu, J.L. Han, Y Liu & X.Y. Liang	OK167067	OK168104	–	–
4	0–20 cm	20200711-1-(1)_0–20 cm_B_R2_SC1	CSF20315	*C. hongkongensis*	AA--	S.F. Chen, L.L. Liu, J.L. Han, Y Liu & X.Y. Liang	OK167068	OK168105	–	–
4	0–20 cm	20200711-1-(1)_0–20 cm_B_R2_SC2	CSF20316	*C. hongkongensis*	AA--	S.F. Chen, L.L. Liu, J.L. Han, Y Liu & X.Y. Liang	OK167069	OK168106	–	–

5	0–20 cm	20200711-1-(2)_0–20 cm_A_R1_SC1	CSF20317	*C. hongkongensis*	AA--	S.F. Chen, L.L. Liu, J.L. Han, Y Liu & X.Y. Liang	OK167070	OK168107	–	–
5	0–20 cm	20200711-1-(2)_0–20 cm_A_R1_SC2	CSF20318	*C. hongkongensis*	AA--	S.F. Chen, L.L. Liu, J.L. Han, Y Liu & X.Y. Liang	OK167071	OK168108	–	–
5	0–20 cm	20200711-1-(2)_0–20 cm_A_R2_SC1	CSF20319	*C. hongkongensis*	AA--	S.F. Chen, L.L. Liu, J.L. Han, Y Liu & X.Y. Liang	OK167072	OK168109	–	–
5	0–20 cm	20200711-1-(2)_0–20 cm_A_R2_SC2	CSF20320	*C. hongkongensis*	AA--	S.F. Chen, L.L. Liu, J.L. Han, Y Liu & X.Y. Liang	OK167073	OK168110	–	–
5	0–20 cm	20200711-1-(2)_0–20 cm_B_R2_SC1	CSF20321	*C. hongkongensis*	AA--	S.F. Chen, L.L. Liu, J.L. Han, Y Liu & X.Y. Liang	OK167074	OK168111	–	–
5	0–20 cm	20200711-1-(2)_0–20 cm_B_R2_SC2	CSF20322	*C. hongkongensis*	AA--	S.F. Chen, L.L. Liu, J.L. Han, Y Liu & X.Y. Liang	OK167075	OK168112	–	–
6	0–20 cm	20200711-1-(3)_0–20 cm_A_R1_SC1	CSF20323	*C. aconidialis*	ACAA	S.F. Chen, L.L. Liu, J.L. Han, Y Liu & X.Y. Liang	OK167866	OK168903	OK169157	OK169241
6	0–20 cm	20200711-1-(3)_0–20 cm_A_R2_SC1	CSF20324	*C. aconidialis*	AC--	S.F. Chen, L.L. Liu, J.L. Han, Y Liu & X.Y. Liang	OK167867	OK168904	–	–
6	0–20 cm	20200711-1-(3)_0–20 cm_A_R2_SC2	CSF20325	*C. aconidialis*	AAAA	S.F. Chen, L.L. Liu, J.L. Han, Y Liu & X.Y. Liang	OK167700	OK168737	OK169148	OK169232
6	0–20 cm	20200711-1-(3)_0–20 cm_B_R1_SC1	CSF20326	*C. hongkongensis*	AA--	S.F. Chen, L.L. Liu, J.L. Han, Y Liu & X.Y. Liang	OK167076	OK168113	–	–
6	0–20 cm	20200711-1-(3)_0–20 cm_B_R1_SC2	CSF20327	*C. hongkongensis*	AA--	S.F. Chen, L.L. Liu, J.L. Han, Y Liu & X.Y. Liang	OK167077	OK168114	–	–
6	0–20 cm	20200711-1-(3)_0–20 cm_B_R2_SC1	CSF20328	*C. hongkongensis*	AA--	S.F. Chen, L.L. Liu, J.L. Han, Y Liu & X.Y. Liang	OK167078	OK168115	–	–
6	0–20 cm	20200711-1-(3)_0–20 cm_B_R2_SC2	CSF20329	*C. hongkongensis*	AA--	S.F. Chen, L.L. Liu, J.L. Han, Y Liu & X.Y. Liang	OK167079	OK168116	–	–
6	20–40 cm	20200711-1-(3)_0–40 cm_A_R2_SC1	CSF20330	*C. hongkongensis*	AA--	S.F. Chen, L.L. Liu, J.L. Han, Y Liu & X.Y. Liang	OK167080	OK168117	–	–
6	20–40 cm	20200711-1-(3)_0–40 cm_A_R2_SC2	CSF20331	*C. hongkongensis*	AA--	S.F. Chen, L.L. Liu, J.L. Han, Y Liu & X.Y. Liang	OK167081	OK168118	–	–
6	20–40 cm	20200711-1-(3)_0–40 cm_A_R2_SC3	CSF20332	*C. hongkongensis*	AA--	S.F. Chen, L.L. Liu, J.L. Han, Y Liu & X.Y. Liang	OK167082	OK168119	–	–
6	20–40 cm	20200711-1-(3)_0–40 cm_A_R2_SC4	CSF20333	*C. hongkongensis*	AA--	S.F. Chen, L.L. Liu, J.L. Han, Y Liu & X.Y. Liang	OK167083	OK168120	–	–

6	20–40 cm	20200711-1-(3)_0–40 cm_B_R1_SC1	CSF20334	*C. hongkongensis*	DAAA	S.F. Chen, L.L. Liu, J.L. Han, Y Liu & X.Y. Liang	OK167669	OK168706	OK169144	OK169228
6	20–40 cm	20200711-1-(3)_0–40 cm_B_R1_SC2	CSF20335	*C. hongkongensis*	DA--	S.F. Chen, L.L. Liu, J.L. Han, Y Liu & X.Y. Liang	OK167670	OK168707	–	–
6	20–40 cm	20200711-1-(3)_0–40 cm_B_R1_SC3	CSF20336	*C. hongkongensis*	DA--	S.F. Chen, L.L. Liu, J.L. Han, Y Liu & X.Y. Liang	OK167671	OK168708	–	–
6	20–40 cm	20200711-1-(3)_0–40 cm_B_R1_SC4	CSF20337	*C. hongkongensis*	DA--	S.F. Chen, L.L. Liu, J.L. Han, Y Liu & X.Y. Liang	OK167672	OK168709	–	–
6	20–40 cm	20200711-1-(3)_0–40 cm_B_R2_SC1	CSF20338	*C. kyotensis*	ABBA	S.F. Chen, L.L. Liu, J.L. Han, Y Liu & X.Y. Liang	OK167986	OK169023	OK169165	OK169249
6	20–40 cm	20200711-1-(3)_0–40 cm_B_R2_SC2	CSF20339	*C. hongkongensis*	AA--	S.F. Chen, L.L. Liu, J.L. Han, Y Liu & X.Y. Liang	OK167084	OK168121	–	–
6	20–40 cm	20200711-1-(3)_0–40 cm_B_R2_SC3	CSF20340	*C. hongkongensis*	AA--	S.F. Chen, L.L. Liu, J.L. Han, Y Liu & X.Y. Liang	OK167085	OK168122	–	–
6	20–40 cm	20200711-1-(3)_0–40 cm_B_R2_SC4	CSF20341	*C. kyotensis*	AB--	S.F. Chen, L.L. Liu, J.L. Han, Y Liu & X.Y. Liang	OK167987	OK169024	–	–
6	60–80 cm	20200711-1-(3)_0–80 cm_A_R1_SC1	CSF20342	*C. hongkongensis*	AA--	S.F. Chen, L.L. Liu, J.L. Han, Y Liu & X.Y. Liang	OK167086	OK168123	–	–
6	60–80 cm	20200711-1-(3)_0–80 cm_A_R1_SC2	CSF20343	*C. hongkongensis*	AA--	S.F. Chen, L.L. Liu, J.L. Han, Y Liu & X.Y. Liang	OK167087	OK168124	–	–
6	60–80 cm	20200711-1-(3)_0–80 cm_A_R1_SC3	CSF20344	*C. hongkongensis*	AA--	S.F. Chen, L.L. Liu, J.L. Han, Y Liu & X.Y. Liang	OK167088	OK168125	–	–
6	60–80 cm	20200711-1-(3)_0–80 cm_A_R1_SC4	CSF20345	*C. hongkongensis*	AA--	S.F. Chen, L.L. Liu, J.L. Han, Y Liu & X.Y. Liang	OK167089	OK168126	–	–
7	0–20 cm	20200711-1-(4)_0–20 cm_A_R1_SC1	CSF20350	*C. hongkongensis*	AA--	S.F. Chen, L.L. Liu, J.L. Han, Y Liu & X.Y. Liang	OK167090	OK168127	–	–
7	0–20 cm	20200711-1-(4)_0–20 cm_A_R1_SC2	CSF20351	*C. hongkongensis*	AA--	S.F. Chen, L.L. Liu, J.L. Han, Y Liu & X.Y. Liang	OK167091	OK168128	–	–
7	0–20 cm	20200711-1-(4)_0–20 cm_A_R2_SC1	CSF20352	*C. hongkongensis*	AA--	S.F. Chen, L.L. Liu, J.L. Han, Y Liu & X.Y. Liang	OK167092	OK168129	–	–
7	0–20 cm	20200711-1-(4)_0–20 cm_A_R2_SC2	CSF20353	*C. hongkongensis*	CAAA	S.F. Chen, L.L. Liu, J.L. Han, Y Liu & X.Y. Liang	OK167664	OK168701	OK169140	OK169224
7	0–20 cm	20200711-1-(4)_0–20 cm_B_R1_SC1	CSF20354	*C. hongkongensis*	AA--	S.F. Chen, L.L. Liu, J.L. Han, Y Liu & X.Y. Liang	OK167093	OK168130	–	–

7	0–20 cm	20200711-1-(4)_0–20 cm_B_R1_SC2	CSF20355	*C. hongkongensis*	AA--	S.F. Chen, L.L. Liu, J.L. Han, Y Liu & X.Y. Liang	OK167094	OK168131	–	–
7	0–20 cm	20200711-1-(4)_0–20 cm_B_R2_SC1	CSF20356	*C. hongkongensis*	AA--	S.F. Chen, L.L. Liu, J.L. Han, Y Liu & X.Y. Liang	OK167095	OK168132	–	–
7	0–20 cm	20200711-1-(4)_0–20 cm_B_R2_SC2	CSF20357	*C. hongkongensis*	AA--	S.F. Chen, L.L. Liu, J.L. Han, Y Liu & X.Y. Liang	OK167096	OK168133	–	–
7	20–40 cm	20200711-1-(4)_0–40 cm_B_R1_SC1	CSF20358	*C. hongkongensis*	CAAA	S.F. Chen, L.L. Liu, J.L. Han, Y Liu & X.Y. Liang	OK167665	OK168702	OK169141	OK169225
7	20–40 cm	20200711-1-(4)_0–40 cm_B_R1_SC2	CSF20359	*C. hongkongensis*	CAAA	S.F. Chen, L.L. Liu, J.L. Han, Y Liu & X.Y. Liang	OK167666	OK168703	OK169142	OK169226
7	20–40 cm	20200711-1-(4)_0–40 cm_B_R1_SC3	CSF20360	*C. hongkongensis*	CAAA	S.F. Chen, L.L. Liu, J.L. Han, Y Liu & X.Y. Liang	OK167667	OK168704	OK169143	OK169227
7	20–40 cm	20200711-1-(4)_0–40 cm_B_R1_SC4	CSF20361	*C. hongkongensis*	CA--	S.F. Chen, L.L. Liu, J.L. Han, Y Liu & X.Y. Liang	OK167668	OK168705	–	–
7	40–60 cm	20200711-1-(4)_0–60 cm_B_R1_SC1	CSF20362	*C. hongkongensis*	AA--	S.F. Chen, L.L. Liu, J.L. Han, Y Liu & X.Y. Liang	OK167097	OK168134	–	–
7	40–60 cm	20200711-1-(4)_0–60 cm_B_R1_SC2	CSF20363	*C. hongkongensis*	AA--	S.F. Chen, L.L. Liu, J.L. Han, Y Liu & X.Y. Liang	OK167098	OK168135	–	–
7	40–60 cm	20200711-1-(4)_0–60 cm_B_R1_SC3	CSF20364	*C. hongkongensis*	AA--	S.F. Chen, L.L. Liu, J.L. Han, Y Liu & X.Y. Liang	OK167099	OK168136	–	–
7	40–60 cm	20200711-1-(4)_0–60 cm_B_R1_SC4	CSF20365	*C. hongkongensis*	AA--	S.F. Chen, L.L. Liu, J.L. Han, Y Liu & X.Y. Liang	OK167100	OK168137	–	–
8	0–20 cm	20200711-1-(5)_0–20 cm_A_R1_SC1	CSF20366	*C. hongkongensis*	AA--	S.F. Chen, L.L. Liu, J.L. Han, Y Liu & X.Y. Liang	OK167101	OK168138	–	–
8	0–20 cm	20200711-1-(5)_0–20 cm_A_R1_SC2	CSF20367	*C. hongkongensis*	AA--	S.F. Chen, L.L. Liu, J.L. Han, Y Liu & X.Y. Liang	OK167102	OK168139	–	–
8	0–20 cm	20200711-1-(5)_0–20 cm_A_R2_SC1	CSF20368	*C. hongkongensis*	AA--	S.F. Chen, L.L. Liu, J.L. Han, Y Liu & X.Y. Liang	OK167103	OK168140	–	–
8	0–20 cm	20200711-1-(5)_0–20 cm_A_R2_SC2	CSF20369	*C. hongkongensis*	AA--	S.F. Chen, L.L. Liu, J.L. Han, Y Liu & X.Y. Liang	OK167104	OK168141	–	–
8	0–20 cm	20200711-1-(5)_0–20 cm_B_R1_SC1	CSF20370	*C. hongkongensis*	AA--	S.F. Chen, L.L. Liu, J.L. Han, Y Liu & X.Y. Liang	OK167105	OK168142	–	–
8	0–20 cm	20200711-1-(5)_0–20 cm_B_R1_SC2	CSF20371	*C. hongkongensis*	AA--	S.F. Chen, L.L. Liu, J.L. Han, Y Liu & X.Y. Liang	OK167106	OK168143	–	–

8	0–20 cm	20200711-1-(5)_0–20 cm_B_R2_SC1	CSF20372	*C. kyotensis*	AAAA	S.F. Chen, L.L. Liu, J.L. Han, Y Liu & X.Y. Liang	OK167950	OK168987	OK169160	OK169244
8	0–20 cm	20200711-1-(5)_0–20 cm_B_R2_SC2	CSF20373	*C. kyotensis*	AA--	S.F. Chen, L.L. Liu, J.L. Han, Y Liu & X.Y. Liang	OK167951	OK168988	–	–
8	0–20 cm	20200711-1-(5)_0–20 cm_B_R2_SC3	CSF20374	*C. hongkongensis*	AA--	S.F. Chen, L.L. Liu, J.L. Han, Y Liu & X.Y. Liang	OK167107	OK168144	–	–
8	0–20 cm	20200711-1-(5)_0–20 cm_B_R2_SC4	CSF20375	*C. hongkongensis*	AA--	S.F. Chen, L.L. Liu, J.L. Han, Y Liu & X.Y. Liang	OK167108	OK168145	–	–
9	0–20 cm	20200711-1-(6)_0–20 cm_A_R1_SC1	CSF20376	*C. aconidialis*	ACAA	S.F. Chen, L.L. Liu, J.L. Han, Y Liu & X.Y. Liang	OK167868	OK168905	OK169158	OK169242
9	0–20 cm	20200711-1-(6)_0–20 cm_A_R1_SC2	CSF20377	*C. aconidialis*	AC--	S.F. Chen, L.L. Liu, J.L. Han, Y Liu & X.Y. Liang	OK167869	OK168906	–	–
9	0–20 cm	20200711-1-(6)_0–20 cm_A_R2_SC1	CSF20378	*C. aconidialis*	AACA	S.F. Chen, L.L. Liu, J.L. Han, Y Liu & X.Y. Liang	OK167701	OK168738	OK169149	OK169233
9	0–20 cm	20200711-1-(6)_0–20 cm_A_R2_SC2	CSF20379	*C. aconidialis*	AC--	S.F. Chen, L.L. Liu, J.L. Han, Y Liu & X.Y. Liang	OK167870	OK168907	–	–
9	0–20 cm	20200711-1-(6)_0–20 cm_B_R1_SC1	CSF20380	*C. hongkongensis*	AGAA	S.F. Chen, L.L. Liu, J.L. Han, Y Liu & X.Y. Liang	OK167630	OK168667	OK169128	OK169212
9	0–20 cm	20200711-1-(6)_0–20 cm_B_R1_SC2	CSF20381	*C. aconidialis*	AC--	S.F. Chen, L.L. Liu, J.L. Han, Y Liu & X.Y. Liang	OK167871	OK168908	–	–
9	0–20 cm	20200711-1-(6)_0–20 cm_B_R2_SC1	CSF20382	*C. hongkongensis*	AA--	S.F. Chen, L.L. Liu, J.L. Han, Y Liu & X.Y. Liang	OK167109	OK168146	–	–
9	0–20 cm	20200711-1-(6)_0–20 cm_B_R2_SC2	CSF20383	*C. hongkongensis*	DAAA	S.F. Chen, L.L. Liu, J.L. Han, Y Liu & X.Y. Liang	OK167673	OK168710	OK169145	OK169229
9	20–40 cm	20200711-1-(6)_0–40 cm_A_R2_SC1	CSF20384	*C. aconidialis*	AC--	S.F. Chen, L.L. Liu, J.L. Han, Y Liu & X.Y. Liang	OK167872	OK168909	–	–
9	20–40 cm	20200711-1-(6)_0–40 cm_A_R2_SC2	CSF20385	*C. aconidialis*	AC--	S.F. Chen, L.L. Liu, J.L. Han, Y Liu & X.Y. Liang	OK167873	OK168910	–	–
9	20–40 cm	20200711-1-(6)_0–40 cm_A_R2_SC3	CSF20386	*C. aconidialis*	AC--	S.F. Chen, L.L. Liu, J.L. Han, Y Liu & X.Y. Liang	OK167874	OK168911	–	–
9	20–40 cm	20200711-1-(6)_0–40 cm_A_R2_SC4	CSF20387	*C. aconidialis*	AC--	S.F. Chen, L.L. Liu, J.L. Han, Y Liu & X.Y. Liang	OK167875	OK168912	–	–
9	40–60 cm	20200711-1-(6)_0–60 cm_B_R1_SC1	CSF20388	*C. aconidialis*	AC--	S.F. Chen, L.L. Liu, J.L. Han, Y Liu & X.Y. Liang	OK167876	OK168913	–	–

9	40–60 cm	20200711-1-(6)_0–60 cm_B_R1_SC2	CSF20389	*C. aconidialis*	AA--	S.F. Chen, L.L. Liu, J.L. Han, Y Liu & X.Y. Liang	OK167702	OK168739	–	–
9	40–60 cm	20200711-1-(6)_0–60 cm_B_R1_SC3	CSF20390	*C. aconidialis*	AC--	S.F. Chen, L.L. Liu, J.L. Han, Y Liu & X.Y. Liang	OK167877	OK168914	–	–
9	40–60 cm	20200711-1-(6)_0–60 cm_B_R1_SC4	CSF20391	*C. aconidialis*	AA--	S.F. Chen, L.L. Liu, J.L. Han, Y Liu & X.Y. Liang	OK167703	OK168740	–	–
9	80–100 cm	20200711-1-(6)_0–100 cm_B_R1_SC1	CSF20396	*C. aconidialis*	AC--	S.F. Chen, L.L. Liu, J.L. Han, Y Liu & X.Y. Liang	OK167878	OK168915	–	–
9	80–100 cm	20200711-1-(6)_0–100 cm_B_R1_SC2	CSF20397	*C. aconidialis*	AC--	S.F. Chen, L.L. Liu, J.L. Han, Y Liu & X.Y. Liang	OK167879	OK168916	–	–
9	80–100 cm	20200711-1-(6)_0–100 cm_B_R1_SC3	CSF20398	*C. aconidialis*	AC--	S.F. Chen, L.L. Liu, J.L. Han, Y Liu & X.Y. Liang	OK167880	OK168917	–	–
9	80–100 cm	20200711-1-(6)_0–100 cm_B_R1_SC4	CSF20399	*C. aconidialis*	AC--	S.F. Chen, L.L. Liu, J.L. Han, Y Liu & X.Y. Liang	OK167881	OK168918	–	–
9	80–100 cm	20200711-1-(6)_0–100 cm_B_R2_SC1	CSF20400	*C. hongkongensis*	DA--	S.F. Chen, L.L. Liu, J.L. Han, Y Liu & X.Y. Liang	OK167674	OK168711	–	–
9	80–100 cm	20200711-1-(6)_0–100 cm_B_R2_SC2	CSF20401	*C. hongkongensis*	DA--	S.F. Chen, L.L. Liu, J.L. Han, Y Liu & X.Y. Liang	OK167675	OK168712	–	–
9	80–100 cm	20200711-1-(6)_0–100 cm_B_R2_SC3	CSF20402	*C. hongkongensis*	DA--	S.F. Chen, L.L. Liu, J.L. Han, Y Liu & X.Y. Liang	OK167676	OK168713	–	–
9	80–100 cm	20200711-1-(6)_0–100 cm_B_R2_SC4	CSF20403	*C. hongkongensis*	DA--	S.F. Chen, L.L. Liu, J.L. Han, Y Liu & X.Y. Liang	OK167677	OK168714	–	–
10	0–20 cm	20200711-1-(7)_0–20 cm_A_R1_SC1	CSF20408	*C. hongkongensis*	AA--	S.F. Chen, L.L. Liu, J.L. Han, Y Liu & X.Y. Liang	OK167110	OK168147	–	–
10	0–20 cm	20200711-1-(7)_0–20 cm_A_R1_SC2	CSF20409	*C. hongkongensis*	AA--	S.F. Chen, L.L. Liu, J.L. Han, Y Liu & X.Y. Liang	OK167111	OK168148	–	–
10	0–20 cm	20200711-1-(7)_0–20 cm_A_R2_SC1	CSF20410	*C. hongkongensis*	AA--	S.F. Chen, L.L. Liu, J.L. Han, Y Liu & X.Y. Liang	OK167112	OK168149	–	–
10	0–20 cm	20200711-1-(7)_0–20 cm_A_R2_SC2	CSF20411	*C. hongkongensis*	AA--	S.F. Chen, L.L. Liu, J.L. Han, Y Liu & X.Y. Liang	OK167113	OK168150	–	–
10	0–20 cm	20200711-1-(7)_0–20 cm_B_R1_SC1	CSF20412	*C. hongkongensis*	ADAA	S.F. Chen, L.L. Liu, J.L. Han, Y Liu & X.Y. Liang	OK167601	OK168638	OK169118	OK169202
10	0–20 cm	20200711-1-(7)_0–20 cm_B_R1_SC2	CSF20413	*C. hongkongensis*	AA--	S.F. Chen, L.L. Liu, J.L. Han, Y Liu & X.Y. Liang	OK167114	OK168151	–	–

10	0–20 cm	20200711-1-(7)_0–20 cm_B_R2_SC1	CSF20414	*C. hongkongensis*	AA--	S.F. Chen, L.L. Liu, J.L. Han, Y Liu & X.Y. Liang	OK167115	OK168152	–	–
10	0–20 cm	20200711-1-(7)_0–20 cm_B_R2_SC2	CSF20415	*C. hongkongensis*	AA--	S.F. Chen, L.L. Liu, J.L. Han, Y Liu & X.Y. Liang	OK167116	OK168153	–	–
10	20–40 cm	20200711-1-(7)_0–40 cm_A_R2_SC1	CSF20416	*C. hongkongensis*	AA--	S.F. Chen, L.L. Liu, J.L. Han, Y Liu & X.Y. Liang	OK167117	OK168154	–	–
10	20–40 cm	20200711-1-(7)_0–40 cm_A_R2_SC2	CSF20417	*C. hongkongensis*	AA--	S.F. Chen, L.L. Liu, J.L. Han, Y Liu & X.Y. Liang	OK167118	OK168155	–	–
10	20–40 cm	20200711-1-(7)_0–40 cm_A_R2_SC3	CSF20418	*C. hongkongensis*	AA--	S.F. Chen, L.L. Liu, J.L. Han, Y Liu & X.Y. Liang	OK167119	OK168156	–	–
10	20–40 cm	20200711-1-(7)_0–40 cm_A_R2_SC4	CSF20419	*C. hongkongensis*	AA--	S.F. Chen, L.L. Liu, J.L. Han, Y Liu & X.Y. Liang	OK167120	OK168157	–	–
10	20–40 cm	20200711-1-(7)_0–40 cm_B_R1_SC1	CSF20420	*C. hongkongensis*	AA--	S.F. Chen, L.L. Liu, J.L. Han, Y Liu & X.Y. Liang	OK167121	OK168158	–	–
10	20–40 cm	20200711-1-(7)_0–40 cm_B_R1_SC2	CSF20421	*C. hongkongensis*	AA--	S.F. Chen, L.L. Liu, J.L. Han, Y Liu & X.Y. Liang	OK167122	OK168159	–	–
10	20–40 cm	20200711-1-(7)_0–40 cm_B_R1_SC3	CSF20422	*C. hongkongensis*	AA--	S.F. Chen, L.L. Liu, J.L. Han, Y Liu & X.Y. Liang	OK167123	OK168160	–	–
10	20–40 cm	20200711-1-(7)_0–40 cm_B_R1_SC4	CSF20423	*C. hongkongensis*	AA--	S.F. Chen, L.L. Liu, J.L. Han, Y Liu & X.Y. Liang	OK167124	OK168161	–	–
10	20–40 cm	20200711-1-(7)_0–40 cm_B_R2_SC1	CSF20424	*C. hongkongensis*	AA--	S.F. Chen, L.L. Liu, J.L. Han, Y Liu & X.Y. Liang	OK167125	OK168162	–	–
10	20–40 cm	20200711-1-(7)_0–40 cm_B_R2_SC2	CSF20425	*C. hongkongensis*	AA--	S.F. Chen, L.L. Liu, J.L. Han, Y Liu & X.Y. Liang	OK167126	OK168163	–	–
10	20–40 cm	20200711-1-(7)_0–40 cm_B_R2_SC3	CSF20426	*C. hongkongensis*	AA--	S.F. Chen, L.L. Liu, J.L. Han, Y Liu & X.Y. Liang	OK167127	OK168164	–	–
10	20–40 cm	20200711-1-(7)_0–40 cm_B_R2_SC4	CSF20427	*C. hongkongensis*	AA--	S.F. Chen, L.L. Liu, J.L. Han, Y Liu & X.Y. Liang	OK167128	OK168165	–	–
10	40–60 cm	20200711-1-(7)_0–60 cm_B_R1_SC1	CSF20428	*C. hongkongensis*	AA--	S.F. Chen, L.L. Liu, J.L. Han, Y Liu & X.Y. Liang	OK167129	OK168166	–	–
10	40–60 cm	20200711-1-(7)_0–60 cm_B_R1_SC2	CSF20429	*C. hongkongensis*	AA--	S.F. Chen, L.L. Liu, J.L. Han, Y Liu & X.Y. Liang	OK167130	OK168167	–	–
10	40–60 cm	20200711-1-(7)_0–60 cm_B_R1_SC3	CSF20430	*C. hongkongensis*	AA--	S.F. Chen, L.L. Liu, J.L. Han, Y Liu & X.Y. Liang	OK167131	OK168168	–	–

10	40–60 cm	20200711-1-(7)_0–60 cm_B_R1_SC4	CSF20431	*C. hongkongensis*	AA--	S.F. Chen, L.L. Liu, J.L. Han, Y Liu & X.Y. Liang	OK167132	OK168169	–	–
10	40–60 cm	20200711-1-(7)_0–60 cm_B_R2_SC1	CSF20432	*C. kyotensis*	ABAB	S.F. Chen, L.L. Liu, J.L. Han, Y Liu & X.Y. Liang	OK167988	OK169025	OK169166	OK169250
10	40–60 cm	20200711-1-(7)_0–60 cm_B_R2_SC2	CSF20433	*C. kyotensis*	AB--	S.F. Chen, L.L. Liu, J.L. Han, Y Liu & X.Y. Liang	OK167989	OK169026	–	–
10	40–60 cm	20200711-1-(7)_0–60 cm_B_R2_SC3	CSF20434	*C. kyotensis*	AB--	S.F. Chen, L.L. Liu, J.L. Han, Y Liu & X.Y. Liang	OK167990	OK169027	–	–
10	80–100 cm	20200711-1-(7)_0–100 cm_A_R1_SC1	CSF20436	*C. hongkongensis*	AA--	S.F. Chen, L.L. Liu, J.L. Han, Y Liu & X.Y. Liang	OK167133	OK168170	–	–
10	80–100 cm	20200711-1-(7)_0–100 cm_A_R1_SC2	CSF20437	*C. hongkongensis*	AA--	S.F. Chen, L.L. Liu, J.L. Han, Y Liu & X.Y. Liang	OK167134	OK168171	–	–
10	80–100 cm	20200711-1-(7)_0–100 cm_A_R1_SC3	CSF20438	*C. hongkongensis*	AA--	S.F. Chen, L.L. Liu, J.L. Han, Y Liu & X.Y. Liang	OK167135	OK168172	–	–
10	80–100 cm	20200711-1-(7)_0–100 cm_A_R1_SC4	CSF20439	*C. hongkongensis*	AA--	S.F. Chen, L.L. Liu, J.L. Han, Y Liu & X.Y. Liang	OK167136	OK168173	–	–
11	0–20 cm	20200715-1-(1)_0–20 cm_A_R1_SC1	CSF20440	*C. hongkongensis*	AA--	S.F. Chen, L.L. Liu, J.L. Han, L.S. Sun & W.W. Li	OK167137	OK168174	–	–
11	0–20 cm	20200715-1-(1)_0–20 cm_A_R1_SC2	CSF20441	*C. hongkongensis*	AGAA	S.F. Chen, L.L. Liu, J.L. Han, L.S. Sun & W.W. Li	OK167631	OK168668	OK169129	OK169213
11	0–20 cm	20200715-1-(1)_0–20 cm_A_R2_SC1	CSF20442	*C. hongkongensis*	AA--	S.F. Chen, L.L. Liu, J.L. Han, L.S. Sun & W.W. Li	OK167138	OK168175	–	–
11	0–20 cm	20200715-1-(1)_0–20 cm_A_R2_SC2	CSF20443	*C. kyotensis*	AAAA	S.F. Chen, L.L. Liu, J.L. Han, L.S. Sun & W.W. Li	OK167952	OK168989	OK169161	OK169245
11	0–20 cm	20200715-1-(1)_0–20 cm_B_R1_SC1	CSF20444	*C. hongkongensis*	DAAA	S.F. Chen, L.L. Liu, J.L. Han, L.S. Sun & W.W. Li	OK167678	OK168715	OK169146	OK169230
11	0–20 cm	20200715-1-(1)_0–20 cm_B_R1_SC2	CSF20445	*C. hongkongensis*	AA--	S.F. Chen, L.L. Liu, J.L. Han, L.S. Sun & W.W. Li	OK167139	OK168176	–	–
11	0–20 cm	20200715-1-(1)_0–20 cm_B_R2_SC1	CSF20446	*C. hongkongensis*	AA--	S.F. Chen, L.L. Liu, J.L. Han, L.S. Sun & W.W. Li	OK167140	OK168177	–	–
11	0–20 cm	20200715-1-(1)_0–20 cm_B_R2_SC2	CSF20447	*C. aconidialis*	AACA	S.F. Chen, L.L. Liu, J.L. Han, L.S. Sun & W.W. Li	OK167704	OK168741	OK169150	OK169234
11	20–40 cm	20200715-1-(1)_0–40 cm_A_R1_SC1	CSF20448	*C. hongkongensis*	AA--	S.F. Chen, L.L. Liu, J.L. Han, L.S. Sun & W.W. Li	OK167141	OK168178	–	–

11	20–40 cm	20200715-1-(1)_0–40 cm_A_R1_SC2	CSF20449	*C. hongkongensis*	AA--	S.F. Chen, L.L. Liu, J.L. Han, L.S. Sun & W.W. Li	OK167142	OK168179	–	–
11	20–40 cm	20200715-1-(1)_0–40 cm_A_R1_SC3	CSF20450	*C. hongkongensis*	AA--	S.F. Chen, L.L. Liu, J.L. Han, L.S. Sun & W.W. Li	OK167143	OK168180	–	–
11	20–40 cm	20200715-1-(1)_0–40 cm_A_R1_SC4	CSF20451	*C. hongkongensis*	AA--	S.F. Chen, L.L. Liu, J.L. Han, L.S. Sun & W.W. Li	OK167144	OK168181	–	–
11	20–40 cm	20200715-1-(1)_0–40 cm_A_R2_SC1	CSF20452	*C. hongkongensis*	AA--	S.F. Chen, L.L. Liu, J.L. Han, L.S. Sun & W.W. Li	OK167145	OK168182	–	–
11	20–40 cm	20200715-1-(1)_0–40 cm_A_R2_SC2	CSF20453	*C. hongkongensis*	AA--	S.F. Chen, L.L. Liu, J.L. Han, L.S. Sun & W.W. Li	OK167146	OK168183	–	–
11	20–40 cm	20200715-1-(1)_0–40 cm_A_R2_SC3	CSF20454	*C. hongkongensis*	ADAA	S.F. Chen, L.L. Liu, J.L. Han, L.S. Sun & W.W. Li	OK167602	OK168639	OK169119	OK169203
11	20–40 cm	20200715-1-(1)_0–40 cm_A_R2_SC4	CSF20455	*C. hongkongensis*	AD--	S.F. Chen, L.L. Liu, J.L. Han, L.S. Sun & W.W. Li	OK167603	OK168640	–	–
11	20–40 cm	20200715-1-(1)_0–40 cm_B_R1_SC1	CSF20456	*C. hongkongensis*	AA--	S.F. Chen, L.L. Liu, J.L. Han, L.S. Sun & W.W. Li	OK167147	OK168184	–	–
11	20–40 cm	20200715-1-(1)_0–40 cm_B_R1_SC2	CSF20457	*C. hongkongensis*	AA--	S.F. Chen, L.L. Liu, J.L. Han, L.S. Sun & W.W. Li	OK167148	OK168185	–	–
11	20–40 cm	20200715-1-(1)_0–40 cm_B_R1_SC3	CSF20458	*C. hongkongensis*	AA--	S.F. Chen, L.L. Liu, J.L. Han, L.S. Sun & W.W. Li	OK167149	OK168186	–	–
11	20–40 cm	20200715-1-(1)_0–40 cm_B_R1_SC4	CSF20459	*C. hongkongensis*	AA--	S.F. Chen, L.L. Liu, J.L. Han, L.S. Sun & W.W. Li	OK167150	OK168187	–	–
11	20–40 cm	20200715-1-(1)_0–40 cm_B_R2_SC1	CSF20460	*C. hongkongensis*	AA--	S.F. Chen, L.L. Liu, J.L. Han, L.S. Sun & W.W. Li	OK167151	OK168188	–	–
11	20–40 cm	20200715-1-(1)_0–40 cm_B_R2_SC2	CSF20461	*C. hongkongensis*	AA--	S.F. Chen, L.L. Liu, J.L. Han, L.S. Sun & W.W. Li	OK167152	OK168189	–	–
11	20–40 cm	20200715-1-(1)_0–40 cm_B_R2_SC3	CSF20462	*C. hongkongensis*	AA--	S.F. Chen, L.L. Liu, J.L. Han, L.S. Sun & W.W. Li	OK167153	OK168190	–	–
11	20–40 cm	20200715-1-(1)_0–40 cm_B_R2_SC4	CSF20463	*C. hongkongensis*	AA--	S.F. Chen, L.L. Liu, J.L. Han, L.S. Sun & W.W. Li	OK167154	OK168191	–	–
11	40–60 cm	20200715-1-(1)_0–60 cm_B_R1_SC1	CSF20464	*C. hongkongensis*	AA--	S.F. Chen, L.L. Liu, J.L. Han, L.S. Sun & W.W. Li	OK167155	OK168192	–	–
11	40–60 cm	20200715-1-(1)_0–60 cm_B_R1_SC2	CSF20465	*C. hongkongensis*	AA--	S.F. Chen, L.L. Liu, J.L. Han, L.S. Sun & W.W. Li	OK167156	OK168193	–	–

11	40–60 cm	20200715-1-(1)_0–60 cm_B_R1_SC3	CSF20466	*C. hongkongensis*	AA--	S.F. Chen, L.L. Liu, J.L. Han, L.S. Sun & W.W. Li	OK167157	OK168194	–	–
11	40–60 cm	20200715-1-(1)_0–60 cm_B_R1_SC4	CSF20467	*C. hongkongensis*	AA--	S.F. Chen, L.L. Liu, J.L. Han, L.S. Sun & W.W. Li	OK167158	OK168195	–	–
12	0–20 cm	20200715-1-(2)_0–20 cm_A_R1_SC1	CSF20468	*C. hongkongensis*	AA--	S.F. Chen, L.L. Liu, J.L. Han, L.S. Sun & W.W. Li	OK167159	OK168196	–	–
12	0–20 cm	20200715-1-(2)_0–20 cm_A_R1_SC2	CSF20469	*C. hongkongensis*	AA--	S.F. Chen, L.L. Liu, J.L. Han, L.S. Sun & W.W. Li	OK167160	OK168197	–	–
12	0–20 cm	20200715-1-(2)_0–20 cm_A_R2_SC1	CSF20470	*C. hongkongensis*	AFAA	S.F. Chen, L.L. Liu, J.L. Han, L.S. Sun & W.W. Li	OK167615	OK168652	OK169126	OK169210
12	0–20 cm	20200715-1-(2)_0–20 cm_B_R1_SC1	CSF20472	*C. hongkongensis*	BAAA	S.F. Chen, L.L. Liu, J.L. Han, L.S. Sun & W.W. Li	OK167649	OK168686	OK169136	OK169220
12	0–20 cm	20200715-1-(2)_0–20 cm_B_R1_SC2	CSF20473	*C. hongkongensis*	AA--	S.F. Chen, L.L. Liu, J.L. Han, L.S. Sun & W.W. Li	OK167161	OK168198	–	–
12	0–20 cm	20200715-1-(2)_0–20 cm_B_R2_SC1	CSF20474	*C. hongkongensis*	BA--	S.F. Chen, L.L. Liu, J.L. Han, L.S. Sun & W.W. Li	OK167650	OK168687	–	–
12	0–20 cm	20200715-1-(2)_0–20 cm_B_R2_SC2	CSF20475	*C. hongkongensis*	BA--	S.F. Chen, L.L. Liu, J.L. Han, L.S. Sun & W.W. Li	OK167651	OK168688	–	–
12	20–40 cm	20200715-1-(2)_0–40 cm_B_R1_SC1	CSF20476	*C. hongkongensis*	AA--	S.F. Chen, L.L. Liu, J.L. Han, L.S. Sun & W.W. Li	OK167162	OK168199	–	–
12	20–40 cm	20200715-1-(2)_0–40 cm_B_R1_SC2	CSF20477	*C. hongkongensis*	AA--	S.F. Chen, L.L. Liu, J.L. Han, L.S. Sun & W.W. Li	OK167163	OK168200	–	–
12	20–40 cm	20200715-1-(2)_0–40 cm_B_R1_SC3	CSF20478	*C. hongkongensis*	AA--	S.F. Chen, L.L. Liu, J.L. Han, L.S. Sun & W.W. Li	OK167164	OK168201	–	–
12	20–40 cm	20200715-1-(2)_0–40 cm_B_R1_SC4	CSF20479	*C. hongkongensis*	AA--	S.F. Chen, L.L. Liu, J.L. Han, L.S. Sun & W.W. Li	OK167165	OK168202	–	–
12	40–60 cm	20200715-1-(2)_0–60 cm_B_R2_SC1	CSF20480	*C. hongkongensis*	AA--	S.F. Chen, L.L. Liu, J.L. Han, L.S. Sun & W.W. Li	OK167166	OK168203	–	–
12	40–60 cm	20200715-1-(2)_0–60 cm_B_R2_SC2	CSF20481	*C. hongkongensis*	AA--	S.F. Chen, L.L. Liu, J.L. Han, L.S. Sun & W.W. Li	OK167167	OK168204	–	–
12	40–60 cm	20200715-1-(2)_0–60 cm_B_R2_SC3	CSF20482	*C. hongkongensis*	AA--	S.F. Chen, L.L. Liu, J.L. Han, L.S. Sun & W.W. Li	OK167168	OK168205	–	–
12	40–60 cm	20200715-1-(2)_0–60 cm_B_R2_SC4	CSF20483	*C. hongkongensis*	AA--	S.F. Chen, L.L. Liu, J.L. Han, L.S. Sun & W.W. Li	OK167169	OK168206	–	–

13	0–20 cm	20200715-1-(3)_0–20 cm_A_R1_SC1	CSF20484	*C. hongkongensis*	AA--	S.F. Chen, L.L. Liu, J.L. Han, L.S. Sun & W.W. Li	OK167170	OK168207	–	–
13	0–20 cm	20200715-1-(3)_0–20 cm_A_R1_SC2	CSF20485	*C. aconidialis*	AC--	S.F. Chen, L.L. Liu, J.L. Han, L.S. Sun & W.W. Li	OK167882	OK168919	–	–
13	0–20 cm	20200715-1-(3)_0–20 cm_A_R2_SC1	CSF20486	*C. aconidialis*	AC--	S.F. Chen, L.L. Liu, J.L. Han, L.S. Sun & W.W. Li	OK167883	OK168920	–	–
13	0–20 cm	20200715-1-(3)_0–20 cm_A_R2_SC2	CSF20487	*C. aconidialis*	AC--	S.F. Chen, L.L. Liu, J.L. Han, L.S. Sun & W.W. Li	OK167884	OK168921	–	–
13	0–20 cm	20200715-1-(3)_0–20 cm_B_R1_SC1	CSF20488	*C. hongkongensis*	AA--	S.F. Chen, L.L. Liu, J.L. Han, L.S. Sun & W.W. Li	OK167171	OK168208	–	–
13	0–20 cm	20200715-1-(3)_0–20 cm_B_R2_SC1	CSF20490	*C. hongkongensis*	AA--	S.F. Chen, L.L. Liu, J.L. Han, L.S. Sun & W.W. Li	OK167172	OK168209	–	–
13	0–20 cm	20200715-1-(3)_0–20 cm_B_R2_SC2	CSF20491	*C. hongkongensis*	AA--	S.F. Chen, L.L. Liu, J.L. Han, L.S. Sun & W.W. Li	OK167173	OK168210	–	–
14	0–20 cm	20200715-1-(4)_0–20 cm_A_R1_SC1	CSF20492	*C. aconidialis*	AA--	S.F. Chen, L.L. Liu, J.L. Han, L.S. Sun & W.W. Li	OK167705	OK168742	–	–
14	0–20 cm	20200715-1-(4)_0–20 cm_A_R1_SC2	CSF20493	*C. hongkongensis*	AA--	S.F. Chen, L.L. Liu, J.L. Han, L.S. Sun & W.W. Li	OK167174	OK168211	–	–
14	0–20 cm	20200715-1-(4)_0–20 cm_A_R2_SC1	CSF20494	*C. hongkongensis*	AA--	S.F. Chen, L.L. Liu, J.L. Han, L.S. Sun & W.W. Li	OK167175	OK168212	–	–
14	0–20 cm	20200715-1-(4)_0–20 cm_A_R2_SC2	CSF20495	*C. hongkongensis*	AA--	S.F. Chen, L.L. Liu, J.L. Han, L.S. Sun & W.W. Li	OK167176	OK168213	–	–
14	0–20 cm	20200715-1-(4)_0–20 cm_B_R1_SC1	CSF20496	*C. aconidialis*	AA--	S.F. Chen, L.L. Liu, J.L. Han, L.S. Sun & W.W. Li	OK167706	OK168743	–	–
14	0–20 cm	20200715-1-(4)_0–20 cm_B_R1_SC2	CSF20497	*C. aconidialis*	AA--	S.F. Chen, L.L. Liu, J.L. Han, L.S. Sun & W.W. Li	OK167707	OK168744	–	–
14	0–20 cm	20200715-1-(4)_0–20 cm_B_R2_SC1	CSF20498	*C. hongkongensis*	AA--	S.F. Chen, L.L. Liu, J.L. Han, L.S. Sun & W.W. Li	OK167177	OK168214	–	–
14	0–20 cm	20200715-1-(4)_0–20 cm_B_R2_SC2	CSF20499	*C. aconidialis*	AA--	S.F. Chen, L.L. Liu, J.L. Han, L.S. Sun & W.W. Li	OK167708	OK168745	–	–
14	20–40 cm	20200715-1-(4)_0–40 cm_A_R1_SC1	CSF20500	*C. hongkongensis*	AA--	S.F. Chen, L.L. Liu, J.L. Han, L.S. Sun & W.W. Li	OK167178	OK168215	–	–
14	20–40 cm	20200715-1-(4)_0–40 cm_A_R1_SC2	CSF20501	*C. hongkongensis*	AA--	S.F. Chen, L.L. Liu, J.L. Han, L.S. Sun & W.W. Li	OK167179	OK168216	–	–

14	20–40 cm	20200715-1-(4)_0–40 cm_A_R1_SC3	CSF20502	*C. hongkongensis*	AA--	S.F. Chen, L.L. Liu, J.L. Han, L.S. Sun & W.W. Li	OK167180	OK168217	–	–
14	20–40 cm	20200715-1-(4)_0–40 cm_A_R1_SC4	CSF20503	*C. hongkongensis*	AA--	S.F. Chen, L.L. Liu, J.L. Han, L.S. Sun & W.W. Li	OK167181	OK168218	–	–
14	20–40 cm	20200715-1-(4)_0–40 cm_A_R2_SC2	CSF20505	*C. hongkongensis*	AA--	S.F. Chen, L.L. Liu, J.L. Han, L.S. Sun & W.W. Li	OK167182	OK168219	–	–
14	20–40 cm	20200715-1-(4)_0–40 cm_A_R2_SC3	CSF20506	*C. hongkongensis*	AA--	S.F. Chen, L.L. Liu, J.L. Han, L.S. Sun & W.W. Li	OK167183	OK168220	–	–
14	20–40 cm	20200715-1-(4)_0–40 cm_A_R2_SC4	CSF20507	*C. hongkongensis*	AA--	S.F. Chen, L.L. Liu, J.L. Han, L.S. Sun & W.W. Li	OK167184	OK168221	–	–
14	40–60 cm	20200715-1-(4)_0–60 cm_A_R1_SC1	CSF20508	*C. hongkongensis*	AA--	S.F. Chen, L.L. Liu, J.L. Han, L.S. Sun & W.W. Li	OK167185	OK168222	–	–
14	40–60 cm	20200715-1-(4)_0–60 cm_A_R1_SC2	CSF20509	*C. hongkongensis*	AA--	S.F. Chen, L.L. Liu, J.L. Han, L.S. Sun & W.W. Li	OK167186	OK168223	–	–
14	40–60 cm	20200715-1-(4)_0–60 cm_A_R1_SC3	CSF20510	*C. hongkongensis*	AA--	S.F. Chen, L.L. Liu, J.L. Han, L.S. Sun & W.W. Li	OK167187	OK168224	–	–
14	40–60 cm	20200715-1-(4)_0–60 cm_A_R1_SC4	CSF20511	*C. hongkongensis*	AA--	S.F. Chen, L.L. Liu, J.L. Han, L.S. Sun & W.W. Li	OK167188	OK168225	–	–
14	40–60 cm	20200715-1-(4)_0–60 cm_B_R1_SC1	CSF20512	*C. hongkongensis*	AA--	S.F. Chen, L.L. Liu, J.L. Han, L.S. Sun & W.W. Li	OK167189	OK168226	–	–
14	40–60 cm	20200715-1-(4)_0–60 cm_B_R1_SC2	CSF20513	*C. hongkongensis*	AA--	S.F. Chen, L.L. Liu, J.L. Han, L.S. Sun & W.W. Li	OK167190	OK168227	–	–
14	40–60 cm	20200715-1-(4)_0–60 cm_B_R1_SC4	CSF20515	*C. hongkongensis*	AA--	S.F. Chen, L.L. Liu, J.L. Han, L.S. Sun & W.W. Li	OK167191	OK168228	–	–
15	0–20 cm	20200715-1-(5)_0–20 cm_B_R1_SC1	CSF20516	*C. hongkongensis*	AA--	S.F. Chen, L.L. Liu, J.L. Han, L.S. Sun & W.W. Li	OK167192	OK168229	–	–
16	0–20 cm	20200715-1-(6)_0–20 cm_B_R2_SC1	CSF20518	*C. kyotensis*	AAAB	S.F. Chen, L.L. Liu, J.L. Han, L.S. Sun & W.W. Li	OK167953	OK168990	OK169162	OK169246
16	0–20 cm	20200715-1-(6)_0–20 cm_B_R2_SC2	CSF20519	*C. kyotensis*	AA--	S.F. Chen, L.L. Liu, J.L. Han, L.S. Sun & W.W. Li	OK167954	OK168991	–	–
17	0–20 cm	20200715-1-(7)_0–20 cm_A_R1_SC1	CSF20520	*C. aconidialis*	AC--	S.F. Chen, L.L. Liu, J.L. Han, L.S. Sun & W.W. Li	OK167885	OK168922	–	–
17	0–20 cm	20200715-1-(7)_0–20 cm_A_R1_SC2	CSF20521	*C. hongkongensis*	AA--	S.F. Chen, L.L. Liu, J.L. Han, L.S. Sun & W.W. Li	OK167193	OK168230	–	–

17	0–20 cm	20200715-1-(7)_0–20 cm_A_R2_SC2	CSF20523	*C. hongkongensis*	AA--	S.F. Chen, L.L. Liu, J.L. Han, L.S. Sun & W.W. Li	OK167194	OK168231	–	–
17	0–20 cm	20200715-1-(7)_0–20 cm_B_R1_SC1	CSF20524	*C. hongkongensis*	ACAA	S.F. Chen, L.L. Liu, J.L. Han, L.S. Sun & W.W. Li	OK167597	OK168634	OK169114	OK169198
17	0–20 cm	20200715-1-(7)_0–20 cm_B_R1_SC2	CSF20525	*C. hongkongensis*	ACAA	S.F. Chen, L.L. Liu, J.L. Han, L.S. Sun & W.W. Li	OK167598	OK168635	OK169115	OK169199
17	0–20 cm	20200715-1-(7)_0–20 cm_B_R2_SC1	CSF20526	*C. hongkongensis*	AA--	S.F. Chen, L.L. Liu, J.L. Han, L.S. Sun & W.W. Li	OK167195	OK168232	–	–
17	0–20 cm	20200715-1-(7)_0–20 cm_B_R2_SC2	CSF20527	*C. hongkongensis*	AA--	S.F. Chen, L.L. Liu, J.L. Han, L.S. Sun & W.W. Li	OK167196	OK168233	–	–
17	40–60 cm	20200715-1-(7)_0–60 cm_A_R1_SC1	CSF20528	*C. hongkongensis*	AGAA	S.F. Chen, L.L. Liu, J.L. Han, L.S. Sun & W.W. Li	OK167632	OK168669	OK169130	OK169214
17	40–60 cm	20200715-1-(7)_0–60 cm_A_R1_SC2	CSF20529	*C. hongkongensis*	AG--	S.F. Chen, L.L. Liu, J.L. Han, L.S. Sun & W.W. Li	OK167633	OK168670	–	–
17	40–60 cm	20200715-1-(7)_0–60 cm_A_R1_SC3	CSF20530	*C. hongkongensis*	AG--	S.F. Chen, L.L. Liu, J.L. Han, L.S. Sun & W.W. Li	OK167634	OK168671	–	–
17	40–60 cm	20200715-1-(7)_0–60 cm_A_R1_SC4	CSF20531	*C. hongkongensis*	AG--	S.F. Chen, L.L. Liu, J.L. Han, L.S. Sun & W.W. Li	OK167635	OK168672	–	–
17	40–60 cm	20200715-1-(7)_0–60 cm_B_R2_SC1	CSF20532	*C. hongkongensis*	DA--	S.F. Chen, L.L. Liu, J.L. Han, L.S. Sun & W.W. Li	OK167679	OK168716	–	–
17	40–60 cm	20200715-1-(7)_0–60 cm_B_R2_SC2	CSF20533	*C. hongkongensis*	DA--	S.F. Chen, L.L. Liu, J.L. Han, L.S. Sun & W.W. Li	OK167680	OK168717	–	–
17	40–60 cm	20200715-1-(7)_0–60 cm_B_R2_SC3	CSF20534	*C. hongkongensis*	DA--	S.F. Chen, L.L. Liu, J.L. Han, L.S. Sun & W.W. Li	OK167681	OK168718	–	–
18	0–20 cm	20200716-1-(1)_0–20 cm_A_R1_SC1	CSF20536	*C. aconidialis*	AA--	S.F. Chen, L.L. Liu, J.L. Han & L.S. Sun	OK167709	OK168746	–	–
18	0–20 cm	20200716-1-(1)_0–20 cm_A_R1_SC2	CSF20537	*C. aconidialis*	AA--	S.F. Chen, L.L. Liu, J.L. Han & L.S. Sun	OK167710	OK168747	–	–
18	0–20 cm	20200716-1-(1)_0–20 cm_A_R2_SC1	CSF20538	*C. aconidialis*	AA--	S.F. Chen, L.L. Liu, J.L. Han & L.S. Sun	OK167711	OK168748	–	–
18	0–20 cm	20200716-1-(1)_0–20 cm_A_R2_SC2	CSF20539	*C. aconidialis*	AA--	S.F. Chen, L.L. Liu, J.L. Han & L.S. Sun	OK167712	OK168749	–	–
20	0–20 cm	20200716-1-(3)_0–20 cm_A_R1_SC1	CSF20540	*C. hongkongensis*	AA--	S.F. Chen, L.L. Liu, J.L. Han & L.S. Sun	OK167197	OK168234	–	–

20	0–20 cm	20200716-1-(3)_0–20 cm_A_R1_SC2	CSF20541	*C. hongkongensis*	AA--	S.F. Chen, L.L. Liu, J.L. Han & L.S. Sun	OK167198	OK168235	–	–
20	0–20 cm	20200716-1-(3)_0–20 cm_A_R2_SC1	CSF20542	*C. hongkongensis*	AA--	S.F. Chen, L.L. Liu, J.L. Han & L.S. Sun	OK167199	OK168236	–	–
20	0–20 cm	20200716-1-(3)_0–20 cm_A_R2_SC2	CSF20543	*C. hongkongensis*	AA--	S.F. Chen, L.L. Liu, J.L. Han & L.S. Sun	OK167200	OK168237	–	–
20	0–20 cm	20200716-1-(3)_0–20 cm_B_R2_SC1	CSF20544	*C. hongkongensis*	AA--	S.F. Chen, L.L. Liu, J.L. Han & L.S. Sun	OK167201	OK168238	–	–
20	0–20 cm	20200716-1-(3)_0–20 cm_B_R2_SC2	CSF20545	*C. hongkongensis*	AA--	S.F. Chen, L.L. Liu, J.L. Han & L.S. Sun	OK167202	OK168239	–	–
21	0–20 cm	20200716-1-(4)_0–20 cm_A_R1_SC1	CSF20546	*C. hongkongensis*	AA--	S.F. Chen, L.L. Liu, J.L. Han & L.S. Sun	OK167203	OK168240	–	–
21	0–20 cm	20200716-1-(4)_0–20 cm_A_R1_SC2	CSF20547	*C. hongkongensis*	AA--	S.F. Chen, L.L. Liu, J.L. Han & L.S. Sun	OK167204	OK168241	–	–
21	0–20 cm	20200716-1-(4)_0–20 cm_A_R2_SC1	CSF20548	*C. hongkongensis*	AA--	S.F. Chen, L.L. Liu, J.L. Han & L.S. Sun	OK167205	OK168242	–	–
21	0–20 cm	20200716-1-(4)_0–20 cm_B_R1_SC1	CSF20550	*C. hongkongensis*	AF--	S.F. Chen, L.L. Liu, J.L. Han & L.S. Sun	OK167616	OK168653	–	–
21	0–20 cm	20200716-1-(4)_0–20 cm_B_R1_SC2	CSF20551	*C. hongkongensis*	AF--	S.F. Chen, L.L. Liu, J.L. Han & L.S. Sun	OK167617	OK168654	–	–
21	0–20 cm	20200716-1-(4)_0–20 cm_B_R2_SC1	CSF20552	*C. hongkongensis*	AA--	S.F. Chen, L.L. Liu, J.L. Han & L.S. Sun	OK167206	OK168243	–	–
21	0–20 cm	20200716-1-(4)_0–20 cm_B_R2_SC2	CSF20553	*C. hongkongensis*	AA--	S.F. Chen, L.L. Liu, J.L. Han & L.S. Sun	OK167207	OK168244	–	–
21	20–40 cm	20200716-1-(4)_0–40 cm_A_R1_SC1	CSF20554	*C. hongkongensis*	AA--	S.F. Chen, L.L. Liu, J.L. Han & L.S. Sun	OK167208	OK168245	–	–
21	20–40 cm	20200716-1-(4)_0–40 cm_A_R1_SC2	CSF20555	*C. hongkongensis*	AA--	S.F. Chen, L.L. Liu, J.L. Han & L.S. Sun	OK167209	OK168246	–	–
21	20–40 cm	20200716-1-(4)_0–40 cm_A_R1_SC3	CSF20556	*C. hongkongensis*	AA--	S.F. Chen, L.L. Liu, J.L. Han & L.S. Sun	OK167210	OK168247	–	–
21	20–40 cm	20200716-1-(4)_0–40 cm_A_R1_SC4	CSF20557	*C. hongkongensis*	AA--	S.F. Chen, L.L. Liu, J.L. Han & L.S. Sun	OK167211	OK168248	–	–
21	20–40 cm	20200716-1-(4)_0–40 cm_B_R1_SC1	CSF20558	*C. hongkongensis*	AA--	S.F. Chen, L.L. Liu, J.L. Han & L.S. Sun	OK167212	OK168249	–	–

21	20–40 cm	20200716-1-(4)_0–40 cm_B_R1_SC3	CSF20560	*C. hongkongensis*	AA--	S.F. Chen, L.L. Liu, J.L. Han & L.S. Sun	OK167213	OK168250	–	–
21	20–40 cm	20200716-1-(4)_0–40 cm_B_R1_SC4	CSF20561	*C. hongkongensis*	AA--	S.F. Chen, L.L. Liu, J.L. Han & L.S. Sun	OK167214	OK168251	–	–
21	20–40 cm	20200716-1-(4)_0–40 cm_B_R2_SC1	CSF20562	*C. hongkongensis*	AA--	S.F. Chen, L.L. Liu, J.L. Han & L.S. Sun	OK167215	OK168252	–	–
21	20–40 cm	20200716-1-(4)_0–40 cm_B_R2_SC2	CSF20563	*C. hongkongensis*	AA--	S.F. Chen, L.L. Liu, J.L. Han & L.S. Sun	OK167216	OK168253	–	–
21	20–40 cm	20200716-1-(4)_0–40 cm_B_R2_SC3	CSF20564	*C. hongkongensis*	AA--	S.F. Chen, L.L. Liu, J.L. Han & L.S. Sun	OK167217	OK168254	–	–
21	20–40 cm	20200716-1-(4)_0–40 cm_B_R2_SC4	CSF20565	*C. hongkongensis*	AA--	S.F. Chen, L.L. Liu, J.L. Han & L.S. Sun	OK167218	OK168255	–	–
22	40–60 cm	20200716-1-(5)_0–60 cm_B_R2_SC1	CSF20566	*C. hongkongensis*	AA--	S.F. Chen, L.L. Liu, J.L. Han & L.S. Sun	OK167219	OK168256	–	–
22	40–60 cm	20200716-1-(5)_0–60 cm_B_R2_SC2	CSF20567	*C. hongkongensis*	AA--	S.F. Chen, L.L. Liu, J.L. Han & L.S. Sun	OK167220	OK168257	–	–
22	40–60 cm	20200716-1-(5)_0–60 cm_B_R2_SC3	CSF20568	*C. hongkongensis*	AA--	S.F. Chen, L.L. Liu, J.L. Han & L.S. Sun	OK167221	OK168258	–	–
22	40–60 cm	20200716-1-(5)_0–60 cm_B_R2_SC4	CSF20569	*C. hongkongensis*	AA--	S.F. Chen, L.L. Liu, J.L. Han & L.S. Sun	OK167222	OK168259	–	–
25	0–20 cm	20200727-1-(1)_0–20 cm_A_R2_SC1	CSF20570	*C. hongkongensis*	AA--	L.L. Liu, J.L. Han & L.S. Sun	OK167223	OK168260	–	–
25	0–20 cm	20200727-1-(1)_0–20 cm_A_R2_SC2	CSF20571	*C. aconidialis*	AA--	L.L. Liu, J.L. Han & L.S. Sun	OK167713	OK168750	–	–
25	0–20 cm	20200727-1-(1)_0–20 cm_B_R1_SC1	CSF20572	*C. hongkongensis*	AA--	L.L. Liu, J.L. Han & L.S. Sun	OK167224	OK168261	–	–
25	0–20 cm	20200727-1-(1)_0–20 cm_B_R1_SC2	CSF20573	*C. hongkongensis*	AA--	L.L. Liu, J.L. Han & L.S. Sun	OK167225	OK168262	–	–
25	0–20 cm	20200727-1-(1)_0–20 cm_B_R2_SC1	CSF20574	*C. aconidialis*	AA--	L.L. Liu, J.L. Han & L.S. Sun	OK167714	OK168751	–	–
25	0–20 cm	20200727-1-(1)_0–20 cm_B_R2_SC2	CSF20575	*C. aconidialis*	AC--	L.L. Liu, J.L. Han & L.S. Sun	OK167886	OK168923	–	–
27	0–20 cm	20200727-1-(3)_0–20 cm_A_R1_SC1	CSF20576	*C. hongkongensis*	AA--	L.L. Liu, J.L. Han & L.S. Sun	OK167226	OK168263	–	–

27	0–20 cm	20200727-1-(3)_0–20 cm_A_R1_SC2	CSF20577	*C. hongkongensis*	AA--	L.L. Liu, J.L. Han & L.S. Sun	OK167227	OK168264	–	–
27	0–20 cm	20200727-1-(3)_0–20 cm_A_R2_SC1	CSF20578	*C. aconidialis*	AA--	L.L. Liu, J.L. Han & L.S. Sun	OK167715	OK168752	–	–
27	0–20 cm	20200727-1-(3)_0–20 cm_A_R2_SC2	CSF20579	*C. aconidialis*	AA--	L.L. Liu, J.L. Han & L.S. Sun	OK167716	OK168753	–	–
27	0–20 cm	20200727-1-(3)_0–20 cm_B_R2_SC1	CSF20580	*C. aconidialis*	AA--	L.L. Liu, J.L. Han & L.S. Sun	OK167717	OK168754	–	–
27	0–20 cm	20200727-1-(3)_0–20 cm_B_R2_SC2	CSF20581	*C. aconidialis*	AA--	L.L. Liu, J.L. Han & L.S. Sun	OK167718	OK168755	–	–
27	40–60 cm	20200727-1-(3)_0–60 cm_B_R1_SC1	CSF20582	*C. aconidialis*	AA--	L.L. Liu, J.L. Han & L.S. Sun	OK167719	OK168756	–	–
27	40–60 cm	20200727-1-(3)_0–60 cm_B_R1_SC2	CSF20583	*C. aconidialis*	AA--	L.L. Liu, J.L. Han & L.S. Sun	OK167720	OK168757	–	–
27	40–60 cm	20200727-1-(3)_0–60 cm_B_R1_SC3	CSF20584	*C. aconidialis*	AA--	L.L. Liu, J.L. Han & L.S. Sun	OK167721	OK168758	–	–
27	40–60 cm	20200727-1-(3)_0–60 cm_B_R1_SC4	CSF20585	*C. aconidialis*	AA--	L.L. Liu, J.L. Han & L.S. Sun	OK167722	OK168759	–	–
28	0–20 cm	20200727-1-(4)_0–20 cm_A_R1_SC1	CSF20586	*C. hongkongensis*	AA--	L.L. Liu, J.L. Han & L.S. Sun	OK167228	OK168265	–	–
28	0–20 cm	20200727-1-(4)_0–20 cm_A_R1_SC2	CSF20587	*C. hongkongensis*	AA--	L.L. Liu, J.L. Han & L.S. Sun	OK167229	OK168266	–	–
28	0–20 cm	20200727-1-(4)_0–20 cm_A_R2_SC1	CSF20588	*C. hongkongensis*	AA--	L.L. Liu, J.L. Han & L.S. Sun	OK167230	OK168267	–	–
28	0–20 cm	20200727-1-(4)_0–20 cm_A_R2_SC2	CSF20589	*C. hongkongensis*	AA--	L.L. Liu, J.L. Han & L.S. Sun	OK167231	OK168268	–	–
28	20–40 cm	20200727-1-(4)_0–40 cm_B_R2_SC1	CSF20590	*C. hongkongensis*	AA--	L.L. Liu, J.L. Han & L.S. Sun	OK167232	OK168269	–	–
28	20–40 cm	20200727-1-(4)_0–40 cm_B_R2_SC3	CSF20592	*C. hongkongensis*	AA--	L.L. Liu, J.L. Han & L.S. Sun	OK167233	OK168270	–	–
28	20–40 cm	20200727-1-(4)_0–40 cm_B_R2_SC4	CSF20593	*C. hongkongensis*	AA--	L.L. Liu, J.L. Han & L.S. Sun	OK167234	OK168271	–	–
29	0–20 cm	20200727-1-(5)_0–20 cm_A_R2_SC1	CSF20594	*C. ilicicola*	AAAB	L.L. Liu, J.L. Han & L.S. Sun	OK168008	OK169045	OK169172	OK169256

29	0–20 cm	20200727-1-(5)_0–20 cm_A_R2_SC2	CSF20595	*C. ilicicola*	AA--	L.L. Liu, J.L. Han & L.S. Sun	OK168009	OK169046	–	–
30	0–20 cm	20200727-1-(6)_0–20 cm_A_R2_SC1	CSF20596	*C. aconidialis*	AA--	L.L. Liu, J.L. Han & L.S. Sun	OK167723	OK168760	–	–
30	0–20 cm	20200727-1-(6)_0–20 cm_A_R2_SC2	CSF20597	*C. aconidialis*	AA--	L.L. Liu, J.L. Han & L.S. Sun	OK167724	OK168761	–	–
31	0–20 cm	20200729-1-(1)_0–20 cm_B_R1_SC1	CSF20598	*C. aconidialis*	AA--	L.L. Liu, J.L. Han, L.S. Sun, Y Liu & X.Y. Liang	OK167725	OK168762	–	–
31	0–20 cm	20200729-1-(1)_0–20 cm_B_R1_SC2	CSF20599	*C. aconidialis*	AA--	L.L. Liu, J.L. Han, L.S. Sun, Y Liu & X.Y. Liang	OK167726	OK168763	–	–
31	0–20 cm	20200729-1-(1)_0–20 cm_B_R2_SC1	CSF20600	*C. hongkongensis*	AA--	L.L. Liu, J.L. Han, L.S. Sun, Y Liu & X.Y. Liang	OK167235	OK168272	–	–
31	0–20 cm	20200729-1-(1)_0–20 cm_B_R2_SC2	CSF20601	*C. hongkongensis*	AA--	L.L. Liu, J.L. Han, L.S. Sun, Y Liu & X.Y. Liang	OK167236	OK168273	–	–
31	40–60 cm	20200729-1-(1)_0–60 cm_A_R1_SC1	CSF20602	*C. orientalis*	AAAA	L.L. Liu, J.L. Han, L.S. Sun, Y Liu & X.Y. Liang	OK168057	OK169094	OK169186	OK169270
31	40–60 cm	20200729-1-(1)_0–60 cm_A_R1_SC2	CSF20603	*C. orientalis*	AAAA	L.L. Liu, J.L. Han, L.S. Sun, Y Liu & X.Y. Liang	OK168058	OK169095	OK169187	OK169271
31	40–60 cm	20200729-1-(1)_0–60 cm_A_R1_SC3	CSF20604	*C. orientalis*	AA--	L.L. Liu, J.L. Han, L.S. Sun, Y Liu & X.Y. Liang	OK168059	OK169096	–	–
31	40–60 cm	20200729-1-(1)_0–60 cm_A_R1_SC4	CSF20605	*C. orientalis*	AA--	L.L. Liu, J.L. Han, L.S. Sun, Y Liu & X.Y. Liang	OK168060	OK169097	–	–
31	40–60 cm	20200729-1-(1)_0–60 cm_B_R1_SC1	CSF20606	*C. orientalis*	AAAA	L.L. Liu, J.L. Han, L.S. Sun, Y Liu & X.Y. Liang	OK168061	OK169098	OK169188	OK169272
31	40–60 cm	20200729-1-(1)_0–60 cm_B_R1_SC2	CSF20607	*C. orientalis*	AAAA	L.L. Liu, J.L. Han, L.S. Sun, Y Liu & X.Y. Liang	OK168062	OK169099	OK169189	OK169273
31	40–60 cm	20200729-1-(1)_0–60 cm_B_R1_SC3	CSF20608	*C. orientalis*	AA--	L.L. Liu, J.L. Han, L.S. Sun, Y Liu & X.Y. Liang	OK168063	OK169100	–	–
31	40–60 cm	20200729-1-(1)_0–60 cm_B_R2_SC1	CSF20610	*C. orientalis*	AAAA	L.L. Liu, J.L. Han, L.S. Sun, Y Liu & X.Y. Liang	OK168064	OK169101	OK169190	OK169274
31	40–60 cm	20200729-1-(1)_0–60 cm_B_R2_SC2	CSF20611	*C. orientalis*	AAAA	L.L. Liu, J.L. Han, L.S. Sun, Y Liu & X.Y. Liang	OK168065	OK169102	OK169191	OK169275
31	40–60 cm	20200729-1-(1)_0–60 cm_B_R2_SC3	CSF20612	*C. orientalis*	AA--	L.L. Liu, J.L. Han, L.S. Sun, Y Liu & X.Y. Liang	OK168066	OK169103	–	–

31	40–60 cm	20200729-1-(1)_0–60 cm_B_R2_SC4	CSF20613	*C. orientalis*	AA--	L.L. Liu, J.L. Han, L.S. Sun, Y Liu & X.Y. Liang	OK168067	OK169104	–	–
31	60–80 cm	20200729-1-(1)_0–80 cm_B_R1_SC1	CSF20614	*C. orientalis*	AAAA	L.L. Liu, J.L. Han, L.S. Sun, Y Liu & X.Y. Liang	OK168068	OK169105	OK169192	OK169276
31	60–80 cm	20200729-1-(1)_0–80 cm_B_R1_SC2	CSF20615	*C. orientalis*	AAAA	L.L. Liu, J.L. Han, L.S. Sun, Y Liu & X.Y. Liang	OK168069	OK169106	OK169193	OK169277
31	60–80 cm	20200729-1-(1)_0–80 cm_B_R1_SC3	CSF20616	*C. orientalis*	AA--	L.L. Liu, J.L. Han, L.S. Sun, Y Liu & X.Y. Liang	OK168070	OK169107	–	–
31	60–80 cm	20200729-1-(1)_0–80 cm_B_R1_SC4	CSF20617	*C. orientalis*	AA--	L.L. Liu, J.L. Han, L.S. Sun, Y Liu & X.Y. Liang	OK168071	OK169108	–	–
32	0–20 cm	20200729-1-(2)_0–20 cm_A_R1_SC1	CSF20618	*C. ilicicola*	ABAA	L.L. Liu, J.L. Han, L.S. Sun, Y Liu & X.Y. Liang	OK168034	OK169071	OK169176	OK169260
32	0–20 cm	20200729-1-(2)_0–20 cm_A_R1_SC2	CSF20619	*C. ilicicola*	AB--	L.L. Liu, J.L. Han, L.S. Sun, Y Liu & X.Y. Liang	OK168035	OK169072	–	–
32	0–20 cm	20200729-1-(2)_0–20 cm_A_R2_SC1	CSF20620	*C. ilicicola*	ABAA	L.L. Liu, J.L. Han, L.S. Sun, Y Liu & X.Y. Liang	OK168036	OK169073	OK169177	OK169261
32	0–20 cm	20200729-1-(2)_0–20 cm_A_R2_SC2	CSF20621	*C. ilicicola*	AB--	L.L. Liu, J.L. Han, L.S. Sun, Y Liu & X.Y. Liang	OK168037	OK169074	–	–
32	0–20 cm	20200729-1-(2)_0–20 cm_B_R1_SC1	CSF20622	*C. hongkongensis*	AA--	L.L. Liu, J.L. Han, L.S. Sun, Y Liu & X.Y. Liang	OK167237	OK168274	–	–
32	0–20 cm	20200729-1-(2)_0–20 cm_B_R1_SC2	CSF20623	*C. hongkongensis*	AA--	L.L. Liu, J.L. Han, L.S. Sun, Y Liu & X.Y. Liang	OK167238	OK168275	–	–
32	20–40 cm	20200729-1-(2)_0–40 cm_A_R1_SC1	CSF20624	*C. ilicicola*	ABAA	L.L. Liu, J.L. Han, L.S. Sun, Y Liu & X.Y. Liang	OK168038	OK169075	OK169178	OK169262
32	20–40 cm	20200729-1-(2)_0–40 cm_A_R1_SC2	CSF20625	*C. ilicicola*	AB--	L.L. Liu, J.L. Han, L.S. Sun, Y Liu & X.Y. Liang	OK168039	OK169076	–	–
32	20–40 cm	20200729-1-(2)_0–40 cm_A_R1_SC3	CSF20626	*C. ilicicola*	AB--	L.L. Liu, J.L. Han, L.S. Sun, Y Liu & X.Y. Liang	OK168040	OK169077	–	–
32	20–40 cm	20200729-1-(2)_0–40 cm_A_R1_SC4	CSF20627	*C. ilicicola*	AB--	L.L. Liu, J.L. Han, L.S. Sun, Y Liu & X.Y. Liang	OK168041	OK169078	–	–
32	20–40 cm	20200729-1-(2)_0–40 cm_A_R2_SC1	CSF20628	*C. hongkongensis*	DA--	L.L. Liu, J.L. Han, L.S. Sun, Y Liu & X.Y. Liang	OK167682	OK168719	–	–
32	20–40 cm	20200729-1-(2)_0–40 cm_A_R2_SC2	CSF20629	*C. hongkongensis*	DA--	L.L. Liu, J.L. Han, L.S. Sun, Y Liu & X.Y. Liang	OK167683	OK168720	–	–

32	20–40 cm	20200729-1-(2)_0–40 cm_A_R2_SC3	CSF20630	*C. hongkongensis*	DA--	L.L. Liu, J.L. Han, L.S. Sun, Y Liu & X.Y. Liang	OK167684	OK168721	–	–
32	20–40 cm	20200729-1-(2)_0–40 cm_A_R2_SC4	CSF20631	*C. hongkongensis*	DA--	L.L. Liu, J.L. Han, L.S. Sun, Y Liu & X.Y. Liang	OK167685	OK168722	–	–
35	0–20 cm	20200729-1-(5)_0–20 cm_A_R2_SC1	CSF20632	*C. hongkongensis*	AA--	L.L. Liu, J.L. Han, L.S. Sun, Y Liu & X.Y. Liang	OK167239	OK168276	–	–
35	0–20 cm	20200729-1-(5)_0–20 cm_A_R2_SC2	CSF20633	*C. hongkongensis*	AA--	L.L. Liu, J.L. Han, L.S. Sun, Y Liu & X.Y. Liang	OK167240	OK168277	–	–
35	0–20 cm	20200729-1-(5)_0–20 cm_B_R1_SC1	CSF20634	*C. hongkongensis*	AA--	L.L. Liu, J.L. Han, L.S. Sun, Y Liu & X.Y. Liang	OK167241	OK168278	–	–
35	0–20 cm	20200729-1-(5)_0–20 cm_B_R1_SC2	CSF20635	*C. hongkongensis*	AA--	L.L. Liu, J.L. Han, L.S. Sun, Y Liu & X.Y. Liang	OK167242	OK168279	–	–
36	0–20 cm	20200730-1-(1)_0–20 cm_A_R1_SC1	CSF20636	*C. hongkongensis*	AA--	L.L. Liu, J.L. Han & L.S. Sun	OK167243	OK168280	–	–
36	0–20 cm	20200730-1-(1)_0–20 cm_A_R1_SC2	CSF20637	*C. hongkongensis*	AA--	L.L. Liu, J.L. Han & L.S. Sun	OK167244	OK168281	–	–
36	0–20 cm	20200730-1-(1)_0–20 cm_A_R2_SC1	CSF20638	*C. aconidialis*	AA--	L.L. Liu, J.L. Han & L.S. Sun	OK167727	OK168764	–	–
36	0–20 cm	20200730-1-(1)_0–20 cm_B_R2_SC1	CSF20640	*C. hongkongensis*	AA--	L.L. Liu, J.L. Han & L.S. Sun	OK167245	OK168282	–	–
36	0–20 cm	20200730-1-(1)_0–20 cm_B_R2_SC2	CSF20641	*C. hongkongensis*	AA--	L.L. Liu, J.L. Han & L.S. Sun	OK167246	OK168283	–	–
37	0–20 cm	20200730-1-(2)_0–20 cm_A_R1_SC1	CSF20642	*C. aconidialis*	AA--	L.L. Liu, J.L. Han & L.S. Sun	OK167728	OK168765	–	–
37	0–20 cm	20200730-1-(2)_0–20 cm_A_R1_SC2	CSF20643	*C. aconidialis*	AA--	L.L. Liu, J.L. Han & L.S. Sun	OK167729	OK168766	–	–
37	0–20 cm	20200730-1-(2)_0–20 cm_A_R2_SC1	CSF20644	*C. aconidialis*	AC--	L.L. Liu, J.L. Han & L.S. Sun	OK167887	OK168924	–	–
37	0–20 cm	20200730-1-(2)_0–20 cm_A_R2_SC2	CSF20645	*C. aconidialis*	AC--	L.L. Liu, J.L. Han & L.S. Sun	OK167888	OK168925	–	–
37	0–20 cm	20200730-1-(2)_0–20 cm_B_R1_SC1	CSF20646	*C. aconidialis*	AA--	L.L. Liu, J.L. Han & L.S. Sun	OK167730	OK168767	–	–
37	0–20 cm	20200730-1-(2)_0–20 cm_B_R1_SC2	CSF20647	*C. aconidialis*	AA--	L.L. Liu, J.L. Han & L.S. Sun	OK167731	OK168768	–	–

37	0–20 cm	20200730-1-(2)_0–20 cm_B_R2_SC1	CSF20648	*C. aconidialis*	AA--	L.L. Liu, J.L. Han & L.S. Sun	OK167732	OK168769	–	–
37	0–20 cm	20200730-1-(2)_0–20 cm_B_R2_SC2	CSF20649	*C. aconidialis*	AA--	L.L. Liu, J.L. Han & L.S. Sun	OK167733	OK168770	–	–
39	0–20 cm	20200730-1-(4)_0–20 cm_A_R1_SC1	CSF20650	*C. hongkongensis*	AA--	L.L. Liu, J.L. Han & L.S. Sun	OK167247	OK168284	–	–
39	0–20 cm	20200730-1-(4)_0–20 cm_A_R1_SC2	CSF20651	*C. hongkongensis*	AA--	L.L. Liu, J.L. Han & L.S. Sun	OK167248	OK168285	–	–
39	0–20 cm	20200730-1-(4)_0–20 cm_A_R2_SC1	CSF20652	*C. kyotensis*	AA--	L.L. Liu, J.L. Han & L.S. Sun	OK167955	OK168992	–	–
39	0–20 cm	20200730-1-(4)_0–20 cm_A_R2_SC2	CSF20653	*C. hongkongensis*	AA--	L.L. Liu, J.L. Han & L.S. Sun	OK167249	OK168286	–	–
39	0–20 cm	20200730-1-(4)_0–20 cm_B_R1_SC1	CSF20654	*C. aconidialis*	AC--	L.L. Liu, J.L. Han & L.S. Sun	OK167889	OK168926	–	–
39	0–20 cm	20200730-1-(4)_0–20 cm_B_R1_SC2	CSF20655	*C. aconidialis*	AC--	L.L. Liu, J.L. Han & L.S. Sun	OK167890	OK168927	–	–
39	0–20 cm	20200730-1-(4)_0–20 cm_B_R2_SC1	CSF20656	*C. aconidialis*	AC--	L.L. Liu, J.L. Han & L.S. Sun	OK167891	OK168928	–	–
39	0–20 cm	20200730-1-(4)_0–20 cm_B_R2_SC2	CSF20657	*C. aconidialis*	AC--	L.L. Liu, J.L. Han & L.S. Sun	OK167892	OK168929	–	–
39	20–40 cm	20200730-1-(4)_0–40 cm_B_R2_SC1	CSF20658	*C. hongkongensis*	AA--	L.L. Liu, J.L. Han & L.S. Sun	OK167250	OK168287	–	–
39	20–40 cm	20200730-1-(4)_0–40 cm_B_R2_SC3	CSF20660	*C. hongkongensis*	AA--	L.L. Liu, J.L. Han & L.S. Sun	OK167251	OK168288	–	–
39	20–40 cm	20200730-1-(4)_0–40 cm_B_R2_SC4	CSF20661	*C. hongkongensis*	AA--	L.L. Liu, J.L. Han & L.S. Sun	OK167252	OK168289	–	–
39	20–40 cm	20200730-1-(4)_0–40 cm_B_R2_SC5	CSF20662	*C. hongkongensis*	AA--	L.L. Liu, J.L. Han & L.S. Sun	OK167253	OK168290	–	–
40	0–20 cm	20200730-1-(5)_0–20 cm_B_R1_SC1	CSF20665	*C. aconidialis*	AA--	L.L. Liu, J.L. Han & L.S. Sun	OK167734	OK168771	–	–
40	0–20 cm	20200730-1-(5)_0–20 cm_B_R1_SC2	CSF20666	*C. aconidialis*	AA--	L.L. Liu, J.L. Han & L.S. Sun	OK167735	OK168772	–	–
40	0–20 cm	20200730-1-(5)_0–20 cm_B_R2_SC1	CSF20667	*C. hongkongensis*	AA--	L.L. Liu, J.L. Han & L.S. Sun	OK167254	OK168291	–	–

40	0–20 cm	20200730-1-(5)_0–20 cm_B_R2_SC2	CSF20668	*C. hongkongensis*	AA--	L.L. Liu, J.L. Han & L.S. Sun	OK167255	OK168292	–	–
41	0–20 cm	20200730-1-(6)_0–20 cm_A_R1_SC1	CSF20669	*C. hongkongensis*	AA--	L.L. Liu, J.L. Han & L.S. Sun	OK167256	OK168293	–	–
41	0–20 cm	20200730-1-(6)_0–20 cm_A_R1_SC2	CSF20670	*C. hongkongensis*	AA--	L.L. Liu, J.L. Han & L.S. Sun	OK167257	OK168294	–	–
41	0–20 cm	20200730-1-(6)_0–20 cm_A_R2_SC1	CSF20671	*C. hongkongensis*	AA--	L.L. Liu, J.L. Han & L.S. Sun	OK167258	OK168295	–	–
41	0–20 cm	20200730-1-(6)_0–20 cm_A_R2_SC2	CSF20672	*C. hongkongensis*	AA--	L.L. Liu, J.L. Han & L.S. Sun	OK167259	OK168296	–	–
41	0–20 cm	20200730-1-(6)_0–20 cm_B_R1_SC1	CSF20673	*C. hongkongensis*	AA--	L.L. Liu, J.L. Han & L.S. Sun	OK167260	OK168297	–	–
41	0–20 cm	20200730-1-(6)_0–20 cm_B_R1_SC2	CSF20674	*C. hongkongensis*	AA--	L.L. Liu, J.L. Han & L.S. Sun	OK167261	OK168298	–	–
42	0–20 cm	20200730-1-(7)_0–20 cm_A_R1_SC1	CSF20675	*C. hongkongensis*	AA--	L.L. Liu, J.L. Han & L.S. Sun	OK167262	OK168299	–	–
42	0–20 cm	20200730-1-(7)_0–20 cm_A_R1_SC2	CSF20676	*C. hongkongensis*	AA--	L.L. Liu, J.L. Han & L.S. Sun	OK167263	OK168300	–	–
42	0–20 cm	20200730-1-(7)_0–20 cm_A_R2_SC1	CSF20677	*C. hongkongensis*	AA--	L.L. Liu, J.L. Han & L.S. Sun	OK167264	OK168301	–	–
42	0–20 cm	20200730-1-(7)_0–20 cm_A_R2_SC2	CSF20678	*C. hongkongensis*	AG--	L.L. Liu, J.L. Han & L.S. Sun	OK167636	OK168673	–	–
42	0–20 cm	20200730-1-(7)_0–20 cm_B_R1_SC1	CSF20679	*C. hongkongensis*	AA--	L.L. Liu, J.L. Han & L.S. Sun	OK167265	OK168302	–	–
42	0–20 cm	20200730-1-(7)_0–20 cm_B_R1_SC2	CSF20680	*C. hongkongensis*	AG--	L.L. Liu, J.L. Han & L.S. Sun	OK167637	OK168674	–	–
42	0–20 cm	20200730-1-(7)_0–20 cm_B_R2_SC1	CSF20681	*C. hongkongensis*	AG--	L.L. Liu, J.L. Han & L.S. Sun	OK167638	OK168675	–	–
42	0–20 cm	20200730-1-(7)_0–20 cm_B_R2_SC2	CSF20682	*C. hongkongensis*	AG--	L.L. Liu, J.L. Han & L.S. Sun	OK167639	OK168676	–	–
43	0–20 cm	20200730-1-(8)_0–20 cm_A_R1_SC1	CSF20683	*C. hongkongensis*	AG--	L.L. Liu, J.L. Han & L.S. Sun	OK167640	OK168677	–	–
43	0–20 cm	20200730-1-(8)_0–20 cm_A_R1_SC2	CSF20684	*C. hongkongensis*	AG--	L.L. Liu, J.L. Han & L.S. Sun	OK167641	OK168678	–	–

43	0–20 cm	20200730-1-(8)_0–20 cm_A_R2_SC1	CSF20685	*C. hongkongensis*	AA--	L.L. Liu, J.L. Han & L.S. Sun	OK167266	OK168303	–	–
43	0–20 cm	20200730-1-(8)_0–20 cm_A_R2_SC2	CSF20686	*C. hongkongensis*	AA--	L.L. Liu, J.L. Han & L.S. Sun	OK167267	OK168304	–	–
43	0–20 cm	20200730-1-(8)_0–20 cm_B_R1_SC1	CSF20687	*C. hongkongensis*	AA--	L.L. Liu, J.L. Han & L.S. Sun	OK167268	OK168305	–	–
43	0–20 cm	20200730-1-(8)_0–20 cm_B_R1_SC2	CSF20688	*C. hongkongensis*	AA--	L.L. Liu, J.L. Han & L.S. Sun	OK167269	OK168306	–	–
43	0–20 cm	20200730-1-(8)_0–20 cm_B_R2_SC1	CSF20689	*C. hongkongensis*	AA--	L.L. Liu, J.L. Han & L.S. Sun	OK167270	OK168307	–	–
43	0–20 cm	20200730-1-(8)_0–20 cm_B_R2_SC2	CSF20690	*C. hongkongensis*	AA--	L.L. Liu, J.L. Han & L.S. Sun	OK167271	OK168308	–	–
44	0–20 cm	20200731-1-(1)_0–20 cm_A_R1_SC1	CSF20691	*C. hongkongensis*	AA--	L.L. Liu, J.L. Han & L.S. Sun	OK167272	OK168309	–	–
44	0–20 cm	20200731-1-(1)_0–20 cm_A_R1_SC2	CSF20692	*C. hongkongensis*	AA--	L.L. Liu, J.L. Han & L.S. Sun	OK167273	OK168310	–	–
44	0–20 cm	20200731-1-(1)_0–20 cm_A_R2_SC1	CSF20693	*C. hongkongensis*	AA--	L.L. Liu, J.L. Han & L.S. Sun	OK167274	OK168311	–	–
44	0–20 cm	20200731-1-(1)_0–20 cm_A_R2_SC2	CSF20694	*C. hongkongensis*	AA--	L.L. Liu, J.L. Han & L.S. Sun	OK167275	OK168312	–	–
44	0–20 cm	20200731-1-(1)_0–20 cm_B_R1_SC1	CSF20695	*C. hongkongensis*	AA--	L.L. Liu, J.L. Han & L.S. Sun	OK167276	OK168313	–	–
44	0–20 cm	20200731-1-(1)_0–20 cm_B_R1_SC2	CSF20696	*C. hongkongensis*	AA--	L.L. Liu, J.L. Han & L.S. Sun	OK167277	OK168314	–	–
44	0–20 cm	20200731-1-(1)_0–20 cm_B_R2_SC1	CSF20697	*C. hongkongensis*	AA--	L.L. Liu, J.L. Han & L.S. Sun	OK167278	OK168315	–	–
44	0–20 cm	20200731-1-(1)_0–20 cm_B_R2_SC2	CSF20698	*C. hongkongensis*	AA--	L.L. Liu, J.L. Han & L.S. Sun	OK167279	OK168316	–	–
45	0–20 cm	20200731-1-(2)_0–20 cm_A_R1_SC1	CSF20699	*C. aconidialis*	AA--	L.L. Liu, J.L. Han & L.S. Sun	OK167736	OK168773	–	–
45	0–20 cm	20200731-1-(2)_0–20 cm_A_R1_SC2	CSF20700	*C. aconidialis*	AA--	L.L. Liu, J.L. Han & L.S. Sun	OK167737	OK168774	–	–
45	0–20 cm	20200731-1-(2)_0–20 cm_A_R2_SC1	CSF20701	*C. aconidialis*	AA--	L.L. Liu, J.L. Han & L.S. Sun	OK167738	OK168775	–	–

45	0–20 cm	20200731-1-(2)_0–20 cm_A_R2_SC2	CSF20702	*C. hongkongensis*	AA--	L.L. Liu, J.L. Han & L.S. Sun	OK167280	OK168317	–	–
45	0–20 cm	20200731-1-(2)_0–20 cm_B_R1_SC1	CSF20703	*C. ilicicola*	ABAA	L.L. Liu, J.L. Han & L.S. Sun	OK168042	OK169079	OK169179	OK169263
45	0–20 cm	20200731-1-(2)_0–20 cm_B_R1_SC2	CSF20704	*C. hongkongensis*	AA--	L.L. Liu, J.L. Han & L.S. Sun	OK167281	OK168318	–	–
45	0–20 cm	20200731-1-(2)_0–20 cm_B_R2_SC1	CSF20705	*C. aconidialis*	AA--	L.L. Liu, J.L. Han & L.S. Sun	OK167739	OK168776	–	–
46	0–20 cm	20200731-1-(3)_0–20 cm_A_R1_SC1	CSF20707	*C. hongkongensis*	AA--	L.L. Liu, J.L. Han & L.S. Sun	OK167282	OK168319	–	–
46	0–20 cm	20200731-1-(3)_0–20 cm_A_R1_SC2	CSF20708	*C. hongkongensis*	AA--	L.L. Liu, J.L. Han & L.S. Sun	OK167283	OK168320	–	–
46	0–20 cm	20200731-1-(3)_0–20 cm_B_R1_SC1	CSF20709	*C. hongkongensis*	AA--	L.L. Liu, J.L. Han & L.S. Sun	OK167284	OK168321	–	–
46	0–20 cm	20200731-1-(3)_0–20 cm_B_R1_SC2	CSF20710	*C. hongkongensis*	AA--	L.L. Liu, J.L. Han & L.S. Sun	OK167285	OK168322	–	–
46	0–20 cm	20200731-1-(3)_0–20 cm_B_R2_SC1	CSF20711	*C. hongkongensis*	AG--	L.L. Liu, J.L. Han & L.S. Sun	OK167642	OK168679	–	–
46	0–20 cm	20200731-1-(3)_0–20 cm_B_R2_SC2	CSF20712	*C. hongkongensis*	AG--	L.L. Liu, J.L. Han & L.S. Sun	OK167643	OK168680	–	–
47	0–20 cm	20200731-1-(4)_0–20 cm_A_R2_SC1	CSF20713	*C. aconidialis*	AA--	L.L. Liu, J.L. Han & L.S. Sun	OK167740	OK168777	–	–
47	0–20 cm	20200731-1-(4)_0–20 cm_A_R2_SC2	CSF20714	*C. aconidialis*	AA--	L.L. Liu, J.L. Han & L.S. Sun	OK167741	OK168778	–	–
47	0–20 cm	20200731-1-(4)_0–20 cm_B_R1_SC1	CSF20715	*C. aconidialis*	AA--	L.L. Liu, J.L. Han & L.S. Sun	OK167742	OK168779	–	–
47	0–20 cm	20200731-1-(4)_0–20 cm_B_R1_SC2	CSF20716	*C. aconidialis*	AA--	L.L. Liu, J.L. Han & L.S. Sun	OK167743	OK168780	–	–
47	20–40 cm	20200731-1-(4)_0–40 cm_A_R2_SC1	CSF20717	*C. aconidialis*	AA--	L.L. Liu, J.L. Han & L.S. Sun	OK167744	OK168781	–	–
47	20–40 cm	20200731-1-(4)_0–40 cm_A_R2_SC2	CSF20718	*C. aconidialis*	AA--	L.L. Liu, J.L. Han & L.S. Sun	OK167745	OK168782	–	–
47	20–40 cm	20200731-1-(4)_0–40 cm_A_R2_SC3	CSF20719	*C. aconidialis*	AA--	L.L. Liu, J.L. Han & L.S. Sun	OK167746	OK168783	–	–

47	20–40 cm	20200731-1-(4)_0–40 cm_A_R2_SC4	CSF20720	*C. aconidialis*	AA--	L.L. Liu, J.L. Han & L.S. Sun	OK167747	OK168784	–	–
48	0–20 cm	20200731-1-(5)_0–20 cm_A_R2_SC1	CSF20721	*C. aconidialis*	AA--	L.L. Liu, J.L. Han & L.S. Sun	OK167748	OK168785	–	–
48	0–20 cm	20200731-1-(5)_0–20 cm_A_R2_SC2	CSF20722	*C. aconidialis*	AA--	L.L. Liu, J.L. Han & L.S. Sun	OK167749	OK168786	–	–
48	0–20 cm	20200731-1-(5)_0–20 cm_B_R2_SC1	CSF20723	*C. aconidialis*	AA--	L.L. Liu, J.L. Han & L.S. Sun	OK167750	OK168787	–	–
48	0–20 cm	20200731-1-(5)_0–20 cm_B_R2_SC2	CSF20724	*C. aconidialis*	AA--	L.L. Liu, J.L. Han & L.S. Sun	OK167751	OK168788	–	–
49	0–20 cm	20200731-1-(6)_0–20 cm_A_R1_SC1	CSF20725	*C. hongkongensis*	DA--	L.L. Liu, J.L. Han & L.S. Sun	OK167686	OK168723	–	–
49	0–20 cm	20200731-1-(6)_0–20 cm_A_R1_SC2	CSF20726	*C. hongkongensis*	DA--	L.L. Liu, J.L. Han & L.S. Sun	OK167687	OK168724	–	–
49	0–20 cm	20200731-1-(6)_0–20 cm_B_R2_SC1	CSF20727	*C. aconidialis*	AA--	L.L. Liu, J.L. Han & L.S. Sun	OK167752	OK168789	–	–
50	0–20 cm	20200731-1-(7)_0–20 cm_A_R1_SC1	CSF20729	*C. hongkongensis*	AA--	L.L. Liu, J.L. Han & L.S. Sun	OK167286	OK168323	–	–
50	0–20 cm	20200731-1-(7)_0–20 cm_B_R1_SC1	CSF20730	*C. hongkongensis*	AA--	L.L. Liu, J.L. Han & L.S. Sun	OK167287	OK168324	–	–
50	0–20 cm	20200731-1-(7)_0–20 cm_B_R1_SC2	CSF20731	*C. hongkongensis*	AA--	L.L. Liu, J.L. Han & L.S. Sun	OK167288	OK168325	–	–
50	0–20 cm	20200731-1-(7)_0–20 cm_B_R2_SC1	CSF20732	*C. hongkongensis*	AA--	L.L. Liu, J.L. Han & L.S. Sun	OK167289	OK168326	–	–
50	0–20 cm	20200731-1-(7)_0–20 cm_B_R2_SC2	CSF20733	*C. hongkongensis*	AA--	L.L. Liu, J.L. Han & L.S. Sun	OK167290	OK168327	–	–
51	0–20 cm	20200809-1-(1)_0–20 cm_A_R1_SC1	CSF20734	*C. hongkongensis*	BAAA	L.L. Liu, J.L. Han & L.S. Sun	OK167652	OK168689	OK169137	OK169221
51	0–20 cm	20200809-1-(1)_0–20 cm_A_R1_SC2	CSF20735	*C. hongkongensis*	BA--	L.L. Liu, J.L. Han & L.S. Sun	OK167653	OK168690	–	–
51	0–20 cm	20200809-1-(1)_0–20 cm_A_R2_SC1	CSF20736	*C. hongkongensis*	AA--	L.L. Liu, J.L. Han & L.S. Sun	OK167291	OK168328	–	–
51	0–20 cm	20200809-1-(1)_0–20 cm_A_R2_SC2	CSF20737	*C. hongkongensis*	BA--	L.L. Liu, J.L. Han & L.S. Sun	OK167654	OK168691	–	–

51	0–20 cm	20200809-1-(1)_0–20 cm_B_R1_SC1	CSF20738	*C. hongkongensis*	BA--	L.L. Liu, J.L. Han & L.S. Sun	OK167655	OK168692	–	–
51	0–20 cm	20200809-1-(1)_0–20 cm_B_R1_SC2	CSF20739	*C. hongkongensis*	AA--	L.L. Liu, J.L. Han & L.S. Sun	OK167292	OK168329	–	–
51	0–20 cm	20200809-1-(1)_0–20 cm_B_R2_SC1	CSF20740	*C. hongkongensis*	BA--	L.L. Liu, J.L. Han & L.S. Sun	OK167656	OK168693	–	–
51	0–20 cm	20200809-1-(1)_0–20 cm_B_R2_SC2	CSF20741	*C. hongkongensis*	AA--	L.L. Liu, J.L. Han & L.S. Sun	OK167293	OK168330	–	–
51	20–40 cm	20200809-1-(1)_0–40 cm_A_R1_SC1	CSF20742	*C. aconidialis*	AA--	L.L. Liu, J.L. Han & L.S. Sun	OK167753	OK168790	–	–
51	20–40 cm	20200809-1-(1)_0–40 cm_A_R1_SC2	CSF20743	*C. aconidialis*	AA--	L.L. Liu, J.L. Han & L.S. Sun	OK167754	OK168791	–	–
51	20–40 cm	20200809-1-(1)_0–40 cm_A_R1_SC4	CSF20745	*C. aconidialis*	AA--	L.L. Liu, J.L. Han & L.S. Sun	OK167755	OK168792	–	–
51	20–40 cm	20200809-1-(1)_0–40 cm_A_R1_SC5	CSF20746	*C. aconidialis*	AA--	L.L. Liu, J.L. Han & L.S. Sun	OK167756	OK168793	–	–
51	20–40 cm	20200809-1-(1)_0–40 cm_A_R2_SC1	CSF20750	*C. aconidialis*	AA--	L.L. Liu, J.L. Han & L.S. Sun	OK167757	OK168794	–	–
51	20–40 cm	20200809-1-(1)_0–40 cm_A_R2_SC2	CSF20751	*C. aconidialis*	AA--	L.L. Liu, J.L. Han & L.S. Sun	OK167758	OK168795	–	–
51	20–40 cm	20200809-1-(1)_0–40 cm_A_R2_SC3	CSF20752	*C. aconidialis*	AA--	L.L. Liu, J.L. Han & L.S. Sun	OK167759	OK168796	–	–
51	20–40 cm	20200809-1-(1)_0–40 cm_A_R2_SC4	CSF20753	*C. aconidialis*	AA--	L.L. Liu, J.L. Han & L.S. Sun	OK167760	OK168797	–	–
52	0–20 cm	20200809-1-(2)_0–20 cm_A_R1_SC1	CSF20754	*C. hongkongensis*	AA--	L.L. Liu, J.L. Han & L.S. Sun	OK167294	OK168331	–	–
52	0–20 cm	20200809-1-(2)_0–20 cm_A_R1_SC2	CSF20755	*C. hongkongensis*	AA--	L.L. Liu, J.L. Han & L.S. Sun	OK167295	OK168332	–	–
52	0–20 cm	20200809-1-(2)_0–20 cm_A_R2_SC1	CSF20756	*C. chinensis*	AAAA	L.L. Liu, J.L. Han & L.S. Sun	OK168055	OK169092	OK169184	OK169268
52	0–20 cm	20200809-1-(2)_0–20 cm_A_R2_SC2	CSF20757	*C. hongkongensis*	AA--	L.L. Liu, J.L. Han & L.S. Sun	OK167296	OK168333	–	–
52	0–20 cm	20200809-1-(2)_0–20 cm_A_R2_SC3	CSF20758	*C. hongkongensis*	ABA-	L.L. Liu, J.L. Han & L.S. Sun	OK167596	OK168633	OK169113	–

52	0–20 cm	20200809-1-(2)_0–20 cm_A_R2_SC4	CSF20759	*C. chinensis*	AAAA	L.L. Liu, J.L. Han & L.S. Sun	OK168056	OK169093	OK169185	OK169269
52	0–20 cm	20200809-1-(2)_0–20 cm_B_R1_SC1	CSF20760	*C. hongkongensis*	AHAA	L.L. Liu, J.L. Han & L.S. Sun	OK167645	OK168682	OK169132	OK169216
52	0–20 cm	20200809-1-(2)_0–20 cm_B_R1_SC2	CSF20761	*C. hongkongensis*	AHAA	L.L. Liu, J.L. Han & L.S. Sun	OK167646	OK168683	OK169133	OK169217
52	0–20 cm	20200809-1-(2)_0–20 cm_B_R2_SC1	CSF20762	*C. hongkongensis*	AA--	L.L. Liu, J.L. Han & L.S. Sun	OK167297	OK168334	–	–
52	0–20 cm	20200809-1-(2)_0–20 cm_B_R2_SC2	CSF20763	*C. hongkongensis*	AA--	L.L. Liu, J.L. Han & L.S. Sun	OK167298	OK168335	–	–
53	0–20 cm	20200809-1-(3)_0–20 cm_A_R1_SC2	CSF20765	*C. hongkongensis*	AA--	L.L. Liu, J.L. Han & L.S. Sun	OK167299	OK168336	–	–
53	0–20 cm	20200809-1-(3)_0–20 cm_A_R1_SC3	CSF20766	*C. hongkongensis*	AA--	L.L. Liu, J.L. Han & L.S. Sun	OK167300	OK168337	–	–
53	0–20 cm	20200809-1-(3)_0–20 cm_A_R1_SC4	CSF20767	*C. hongkongensis*	AA--	L.L. Liu, J.L. Han & L.S. Sun	OK167301	OK168338	–	–
53	0–20 cm	20200809-1-(3)_0–20 cm_A_R2_SC1	CSF20768	*C. kyotensis*	AB--	L.L. Liu, J.L. Han & L.S. Sun	OK167991	OK169028	–	–
53	0–20 cm	20200809-1-(3)_0–20 cm_A_R2_SC2	CSF20769	*C. kyotensis*	AB--	L.L. Liu, J.L. Han & L.S. Sun	OK167992	OK169029	–	–
53	0–20 cm	20200809-1-(3)_0–20 cm_B_R1_SC1	CSF20770	*C. hongkongensis*	AA--	L.L. Liu, J.L. Han & L.S. Sun	OK167302	OK168339	–	–
53	0–20 cm	20200809-1-(3)_0–20 cm_B_R1_SC2	CSF20771	*C. hongkongensis*	AA--	L.L. Liu, J.L. Han & L.S. Sun	OK167303	OK168340	–	–
53	0–20 cm	20200809-1-(3)_0–20 cm_B_R2_SC1	CSF20772	*C. hongkongensis*	AA--	L.L. Liu, J.L. Han & L.S. Sun	OK167304	OK168341	–	–
53	0–20 cm	20200809-1-(3)_0–20 cm_B_R2_SC2	CSF20773	*C. hongkongensis*	AA--	L.L. Liu, J.L. Han & L.S. Sun	OK167305	OK168342	–	–
54	0–20 cm	20200809-1-(4)_0–20 cm_B_R2_SC1	CSF20774	*C. kyotensis*	AA--	L.L. Liu, J.L. Han & L.S. Sun	OK167956	OK168993	–	–
54	0–20 cm	20200809-1-(4)_0–20 cm_B_R2_SC2	CSF20775	*C. kyotensis*	AA--	L.L. Liu, J.L. Han & L.S. Sun	OK167957	OK168994	–	–
55	0–20 cm	20200809-1-(5)_0–20 cm_A_R1_SC1	CSF20776	*C. hongkongensis*	AA--	L.L. Liu, J.L. Han & L.S. Sun	OK167306	OK168343	–	–

55	0–20 cm	20200809-1-(5)_0–20 cm_A_R1_SC2	CSF20777	*C. hongkongensis*	AA--	L.L. Liu, J.L. Han & L.S. Sun	OK167307	OK168344	–	–
55	0–20 cm	20200809-1-(5)_0–20 cm_B_R1_SC2	CSF20779	*C. hongkongensis*	AA--	L.L. Liu, J.L. Han & L.S. Sun	OK167308	OK168345	–	–
55	0–20 cm	20200809-1-(5)_0–20 cm_B_R2_SC1	CSF20780	*C. aconidialis*	AC--	L.L. Liu, J.L. Han & L.S. Sun	OK167893	OK168930	–	–
55	40–60 cm	20200809-1-(5)_0–60 cm_B_R2_SC1	CSF20782	*C. hongkongensis*	AF--	L.L. Liu, J.L. Han & L.S. Sun	OK167618	OK168655	–	–
55	40–60 cm	20200809-1-(5)_0–60 cm_B_R2_SC2	CSF20783	*C. hongkongensis*	AF--	L.L. Liu, J.L. Han & L.S. Sun	OK167619	OK168656	–	–
55	40–60 cm	20200809-1-(5)_0–60 cm_B_R2_SC3	CSF20784	*C. hongkongensis*	AF--	L.L. Liu, J.L. Han & L.S. Sun	OK167620	OK168657	–	–
55	40–60 cm	20200809-1-(5)_0–60 cm_B_R2_SC4	CSF20785	*C. hongkongensis*	AF--	L.L. Liu, J.L. Han & L.S. Sun	OK167621	OK168658	–	–
56	0–20 cm	20200809-1-(6)_0–20 cm_A_R2_SC1	CSF20788	*C. hongkongensis*	AA--	L.L. Liu, J.L. Han & L.S. Sun	OK167309	OK168346	–	–
56	0–20 cm	20200809-1-(6)_0–20 cm_A_R2_SC2	CSF20789	*C. hongkongensis*	AA--	L.L. Liu, J.L. Han & L.S. Sun	OK167310	OK168347	–	–
56	0–20 cm	20200809-1-(6)_0–20 cm_B_R1_SC1	CSF20790	*C. hongkongensis*	AA--	L.L. Liu, J.L. Han & L.S. Sun	OK167311	OK168348	–	–
56	0–20 cm	20200809-1-(6)_0–20 cm_B_R2_SC1	CSF20792	*C. hongkongensis*	AA--	L.L. Liu, J.L. Han & L.S. Sun	OK167312	OK168349	–	–
56	0–20 cm	20200809-1-(6)_0–20 cm_B_R2_SC2	CSF20793	*C. hongkongensis*	AA--	L.L. Liu, J.L. Han & L.S. Sun	OK167313	OK168350	–	–
57	0–20 cm	20200810-1-(1)_0–20 cm_A_R1_SC1	CSF20794	*C. aconidialis*	AA--	L.L. Liu, J.L. Han & L.S. Sun	OK167761	OK168798	–	–
57	0–20 cm	20200810-1-(1)_0–20 cm_A_R1_SC2	CSF20795	*C. aconidialis*	AA--	L.L. Liu, J.L. Han & L.S. Sun	OK167762	OK168799	–	–
57	0–20 cm	20200810-1-(1)_0–20 cm_A_R2_SC1	CSF20796	*C. aconidialis*	AA--	L.L. Liu, J.L. Han & L.S. Sun	OK167763	OK168800	–	–
57	0–20 cm	20200810-1-(1)_0–20 cm_A_R2_SC2	CSF20797	*C. hongkongensis*	DA--	L.L. Liu, J.L. Han & L.S. Sun	OK167688	OK168725	–	–
57	0–20 cm	20200810-1-(1)_0–20 cm_B_R1_SC1	CSF20798	*C. hongkongensis*	AA--	L.L. Liu, J.L. Han & L.S. Sun	OK167314	OK168351	–	–

57	0–20 cm	20200810-1-(1)_0–20 cm_B_R1_SC2	CSF20799	*C. hongkongensis*	AA--	L.L. Liu, J.L. Han & L.S. Sun	OK167315	OK168352	–	–
58	0–20 cm	20200810-1-(2)_0–20 cm_A_R1_SC1	CSF20800	*C. hongkongensis*	AA--	L.L. Liu, J.L. Han & L.S. Sun	OK167316	OK168353	–	–
58	0–20 cm	20200810-1-(2)_0–20 cm_A_R1_SC2	CSF20801	*C. hongkongensis*	AA--	L.L. Liu, J.L. Han & L.S. Sun	OK167317	OK168354	–	–
58	0–20 cm	20200810-1-(2)_0–20 cm_A_R2_SC1	CSF20802	*C. hongkongensis*	AA--	L.L. Liu, J.L. Han & L.S. Sun	OK167318	OK168355	–	–
58	0–20 cm	20200810-1-(2)_0–20 cm_A_R2_SC2	CSF20803	*C. hongkongensis*	AA--	L.L. Liu, J.L. Han & L.S. Sun	OK167319	OK168356	–	–
58	0–20 cm	20200810-1-(2)_0–20 cm_B_R1_SC1	CSF20804	*C. hongkongensis*	AA--	L.L. Liu, J.L. Han & L.S. Sun	OK167320	OK168357	–	–
58	0–20 cm	20200810-1-(2)_0–20 cm_B_R1_SC2	CSF20805	*C. hongkongensis*	AA--	L.L. Liu, J.L. Han & L.S. Sun	OK167321	OK168358	–	–
58	0–20 cm	20200810-1-(2)_0–20 cm_B_R2_SC1	CSF20806	*C. hongkongensis*	AA--	L.L. Liu, J.L. Han & L.S. Sun	OK167322	OK168359	–	–
58	0–20 cm	20200810-1-(2)_0–20 cm_B_R2_SC2	CSF20807	*C. hongkongensis*	AA--	L.L. Liu, J.L. Han & L.S. Sun	OK167323	OK168360	–	–
58	20–40 cm	20200810-1-(2)_0–40 cm_A_R1_SC1	CSF20808	*C. hongkongensis*	AA--	L.L. Liu, J.L. Han & L.S. Sun	OK167324	OK168361	–	–
58	20–40 cm	20200810-1-(2)_0–40 cm_A_R1_SC2	CSF20809	*C. hongkongensis*	AA--	L.L. Liu, J.L. Han & L.S. Sun	OK167325	OK168362	–	–
58	20–40 cm	20200810-1-(2)_0–40 cm_A_R1_SC3	CSF20810	*C. hongkongensis*	AA--	L.L. Liu, J.L. Han & L.S. Sun	OK167326	OK168363	–	–
58	20–40 cm	20200810-1-(2)_0–40 cm_A_R1_SC4	CSF20811	*C. hongkongensis*	AA--	L.L. Liu, J.L. Han & L.S. Sun	OK167327	OK168364	–	–
58	20–40 cm	20200810-1-(2)_0–40 cm_A_R2_SC1	CSF20812	*C. hongkongensis*	AA--	L.L. Liu, J.L. Han & L.S. Sun	OK167328	OK168365	–	–
58	20–40 cm	20200810-1-(2)_0–40 cm_A_R2_SC2	CSF20813	*C. hongkongensis*	AA--	L.L. Liu, J.L. Han & L.S. Sun	OK167329	OK168366	–	–
58	20–40 cm	20200810-1-(2)_0–40 cm_A_R2_SC3	CSF20814	*C. hongkongensis*	AA--	L.L. Liu, J.L. Han & L.S. Sun	OK167330	OK168367	–	–
58	20–40 cm	20200810-1-(2)_0–40 cm_A_R2_SC4	CSF20815	*C. hongkongensis*	AA--	L.L. Liu, J.L. Han & L.S. Sun	OK167331	OK168368	–	–

59	0–20 cm	20200810-1-(3)_0–20 cm_A_R1_SC1	CSF20816	*C. hongkongensis*	AA--	L.L. Liu, J.L. Han & L.S. Sun	OK167332	OK168369	–	–
59	0–20 cm	20200810-1-(3)_0–20 cm_A_R1_SC2	CSF20817	*C. hongkongensis*	AA--	L.L. Liu, J.L. Han & L.S. Sun	OK167333	OK168370	–	–
59	0–20 cm	20200810-1-(3)_0–20 cm_A_R2_SC1	CSF20818	*C. hongkongensis*	AA--	L.L. Liu, J.L. Han & L.S. Sun	OK167334	OK168371	–	–
59	0–20 cm	20200810-1-(3)_0–20 cm_A_R2_SC2	CSF20819	*C. hongkongensis*	AA--	L.L. Liu, J.L. Han & L.S. Sun	OK167335	OK168372	–	–
59	0–20 cm	20200810-1-(3)_0–20 cm_B_R1_SC1	CSF20820	*C. hongkongensis*	AA--	L.L. Liu, J.L. Han & L.S. Sun	OK167336	OK168373	–	–
59	0–20 cm	20200810-1-(3)_0–20 cm_B_R1_SC2	CSF20821	*C. hongkongensis*	AA--	L.L. Liu, J.L. Han & L.S. Sun	OK167337	OK168374	–	–
59	0–20 cm	20200810-1-(3)_0–20 cm_B_R2_SC1	CSF20822	*C. hongkongensis*	AA--	L.L. Liu, J.L. Han & L.S. Sun	OK167338	OK168375	–	–
59	0–20 cm	20200810-1-(3)_0–20 cm_B_R2_SC2	CSF20823	*C. hongkongensis*	AA--	L.L. Liu, J.L. Han & L.S. Sun	OK167339	OK168376	–	–
59	60–80 cm	20200810-1-(3)_0–80 cm_A_R1_SC1	CSF20824	*C. aconidialis*	AA--	L.L. Liu, J.L. Han & L.S. Sun	OK167764	OK168801	–	–
59	60–80 cm	20200810-1-(3)_0–80 cm_A_R1_SC2	CSF20825	*C. aconidialis*	AA--	L.L. Liu, J.L. Han & L.S. Sun	OK167765	OK168802	–	–
59	60–80 cm	20200810-1-(3)_0–80 cm_A_R1_SC3	CSF20826	*C. aconidialis*	AA--	L.L. Liu, J.L. Han & L.S. Sun	OK167766	OK168803	–	–
59	60–80 cm	20200810-1-(3)_0–80 cm_A_R1_SC4	CSF20827	*C. aconidialis*	AA--	L.L. Liu, J.L. Han & L.S. Sun	OK167767	OK168804	–	–
60	0–20 cm	20200810-1-(4)_0–20 cm_A_R1_SC1	CSF20832	*C. hongkongensis*	AA--	L.L. Liu, J.L. Han & L.S. Sun	OK167340	OK168377	–	–
60	0–20 cm	20200810-1-(4)_0–20 cm_A_R1_SC2	CSF20833	*C. aconidialis*	AA--	L.L. Liu, J.L. Han & L.S. Sun	OK167768	OK168805	–	–
60	0–20 cm	20200810-1-(4)_0–20 cm_B_R1_SC1	CSF20834	*C. hongkongensis*	ADAA	L.L. Liu, J.L. Han & L.S. Sun	OK167604	OK168641	OK169120	OK169204
60	0–20 cm	20200810-1-(4)_0–20 cm_B_R1_SC2	CSF20835	*C. aconidialis*	AA--	L.L. Liu, J.L. Han & L.S. Sun	OK167769	OK168806	–	–
60	0–20 cm	20200810-1-(4)_0–20 cm_B_R2_SC1	CSF20836	*C. hongkongensis*	AD--	L.L. Liu, J.L. Han & L.S. Sun	OK167605	OK168642	–	–

60	0–20 cm	20200810-1-(4)_0–20 cm_B_R2_SC2	CSF20837	*C. aconidialis*	AA--	L.L. Liu, J.L. Han & L.S. Sun	OK167770	OK168807	–	–
61	0–20 cm	20200810-1-(5)_0–20 cm_A_R1_SC1	CSF20838	*C. aconidialis*	AA--	L.L. Liu, J.L. Han & L.S. Sun	OK167771	OK168808	–	–
61	0–20 cm	20200810-1-(5)_0–20 cm_A_R2_SC1	CSF20840	*C. hongkongensis*	AA--	L.L. Liu, J.L. Han & L.S. Sun	OK167341	OK168378	–	–
61	0–20 cm	20200810-1-(5)_0–20 cm_A_R2_SC2	CSF20841	*C. hongkongensis*	AA--	L.L. Liu, J.L. Han & L.S. Sun	OK167342	OK168379	–	–
61	0–20 cm	20200810-1-(5)_0–20 cm_B_R1_SC1	CSF20842	*C. hongkongensis*	AA--	L.L. Liu, J.L. Han & L.S. Sun	OK167343	OK168380	–	–
61	0–20 cm	20200810-1-(5)_0–20 cm_B_R1_SC2	CSF20843	*C. hongkongensis*	AA--	L.L. Liu, J.L. Han & L.S. Sun	OK167344	OK168381	–	–
61	0–20 cm	20200810-1-(5)_0–20 cm_B_R2_SC1	CSF20844	*C. hongkongensis*	AA--	L.L. Liu, J.L. Han & L.S. Sun	OK167345	OK168382	–	–
61	0–20 cm	20200810-1-(5)_0–20 cm_B_R2_SC2	CSF20845	*C. hongkongensis*	AA--	L.L. Liu, J.L. Han & L.S. Sun	OK167346	OK168383	–	–
61	20–40 cm	20200810-1-(5)_0–40 cm_A_R1_SC5	CSF20850	*C. hongkongensis*	AA--	L.L. Liu, J.L. Han & L.S. Sun	OK167347	OK168384	–	–
61	20–40 cm	20200810-1-(5)_0–40 cm_A_R1_SC6	CSF20851	*C. hongkongensis*	AA--	L.L. Liu, J.L. Han & L.S. Sun	OK167348	OK168385	–	–
61	20–40 cm	20200810-1-(5)_0–40 cm_A_R1_SC7	CSF20852	*C. hongkongensis*	AA--	L.L. Liu, J.L. Han & L.S. Sun	OK167349	OK168386	–	–
61	20–40 cm	20200810-1-(5)_0–40 cm_A_R1_SC8	CSF20853	*C. ilicicola*	BBAA	L.L. Liu, J.L. Han & L.S. Sun	OK168043	OK169080	OK169180	OK169264
61	20–40 cm	20200810-1-(5)_0–40 cm_A_R2_SC1	CSF20854	*C. hongkongensis*	AA--	L.L. Liu, J.L. Han & L.S. Sun	OK167350	OK168387	–	–
61	20–40 cm	20200810-1-(5)_0–40 cm_A_R2_SC2	CSF20855	*C. hongkongensis*	AA--	L.L. Liu, J.L. Han & L.S. Sun	OK167351	OK168388	–	–
61	20–40 cm	20200810-1-(5)_0–40 cm_A_R2_SC3	CSF20856	*C. hongkongensis*	AA--	L.L. Liu, J.L. Han & L.S. Sun	OK167352	OK168389	–	–
61	20–40 cm	20200810-1-(5)_0–40 cm_A_R2_SC4	CSF20857	*C. hongkongensis*	AA--	L.L. Liu, J.L. Han & L.S. Sun	OK167353	OK168390	–	–
61	20–40 cm	20200810-1-(5)_0–40 cm_B_R1_SC1	CSF20858	*C. hongkongensis*	DA--	L.L. Liu, J.L. Han & L.S. Sun	OK167689	OK168726	–	–

61	20–40 cm	20200810-1-(5)_0–40 cm_B_R1_SC2	CSF20859	*C. hongkongensis*	DA--	L.L. Liu, J.L. Han & L.S. Sun	OK167690	OK168727	–	–
61	20–40 cm	20200810-1-(5)_0–40 cm_B_R1_SC3	CSF20860	*C. hongkongensis*	DA--	L.L. Liu, J.L. Han & L.S. Sun	OK167691	OK168728	–	–
61	20–40 cm	20200810-1-(5)_0–40 cm_B_R1_SC4	CSF20861	*C. hongkongensis*	DA--	L.L. Liu, J.L. Han & L.S. Sun	OK167692	OK168729	–	–
61	20–40 cm	20200810-1-(5)_0–40 cm_B_R2_SC1	CSF20862	*C. hongkongensis*	AA--	L.L. Liu, J.L. Han & L.S. Sun	OK167354	OK168391	–	–
61	20–40 cm	20200810-1-(5)_0–40 cm_B_R2_SC2	CSF20863	*C. hongkongensis*	AA--	L.L. Liu, J.L. Han & L.S. Sun	OK167355	OK168392	–	–
61	20–40 cm	20200810-1-(5)_0–40 cm_B_R2_SC3	CSF20864	*C. hongkongensis*	AA--	L.L. Liu, J.L. Han & L.S. Sun	OK167356	OK168393	–	–
61	20–40 cm	20200810-1-(5)_0–40 cm_B_R2_SC4	CSF20865	*C. hongkongensis*	AA--	L.L. Liu, J.L. Han & L.S. Sun	OK167357	OK168394	–	–
62	0–20 cm	20200810-1-(6)_0–20 cm_A_R1_SC1	CSF20866	*C. aconidialis*	AA--	L.L. Liu, J.L. Han & L.S. Sun	OK167772	OK168809	–	–
62	0–20 cm	20200810-1-(6)_0–20 cm_A_R1_SC2	CSF20867	*C. aconidialis*	AA--	L.L. Liu, J.L. Han & L.S. Sun	OK167773	OK168810	–	–
62	0–20 cm	20200810-1-(6)_0–20 cm_A_R2_SC1	CSF20868	*C. aconidialis*	AA--	L.L. Liu, J.L. Han & L.S. Sun	OK167774	OK168811	–	–
62	0–20 cm	20200810-1-(6)_0–20 cm_A_R2_SC2	CSF20869	*C. aconidialis*	AA--	L.L. Liu, J.L. Han & L.S. Sun	OK167775	OK168812	–	–
62	0–20 cm	20200810-1-(6)_0–20 cm_B_R1_SC1	CSF20870	*C. aconidialis*	AA--	L.L. Liu, J.L. Han & L.S. Sun	OK167776	OK168813	–	–
62	0–20 cm	20200810-1-(6)_0–20 cm_B_R1_SC2	CSF20871	*C. aconidialis*	AA--	L.L. Liu, J.L. Han & L.S. Sun	OK167777	OK168814	–	–
62	0–20 cm	20200810-1-(6)_0–20 cm_B_R2_SC1	CSF20872	*C. aconidialis*	AA--	L.L. Liu, J.L. Han & L.S. Sun	OK167778	OK168815	–	–
62	0–20 cm	20200810-1-(6)_0–20 cm_B_R2_SC2	CSF20873	*C. aconidialis*	AA--	L.L. Liu, J.L. Han & L.S. Sun	OK167779	OK168816	–	–
62	20–40 cm	20200810-1-(6)_0–40 cm_A_R1_SC1	CSF20874	*C. aconidialis*	AA--	L.L. Liu, J.L. Han & L.S. Sun	OK167780	OK168817	–	–
62	20–40 cm	20200810-1-(6)_0–40 cm_A_R1_SC2	CSF20875	*C. aconidialis*	AA--	L.L. Liu, J.L. Han & L.S. Sun	OK167781	OK168818	–	–

62	20–40 cm	20200810-1-(6)_0–40 cm_A_R1_SC3	CSF20876	*C. aconidialis*	AA--	L.L. Liu, J.L. Han & L.S. Sun	OK167782	OK168819	–	–
62	20–40 cm	20200810-1-(6)_0–40 cm_A_R1_SC4	CSF20877	*C. aconidialis*	AA--	L.L. Liu, J.L. Han & L.S. Sun	OK167783	OK168820	–	–
62	20–40 cm	20200810-1-(6)_0–40 cm_A_R2_SC1	CSF20878	*C. aconidialis*	AC--	L.L. Liu, J.L. Han & L.S. Sun	OK167894	OK168931	–	–
62	20–40 cm	20200810-1-(6)_0–40 cm_A_R2_SC2	CSF20879	*C. aconidialis*	AA--	L.L. Liu, J.L. Han & L.S. Sun	OK167784	OK168821	–	–
62	20–40 cm	20200810-1-(6)_0–40 cm_A_R2_SC3	CSF20880	*C. aconidialis*	AA--	L.L. Liu, J.L. Han & L.S. Sun	OK167785	OK168822	–	–
62	20–40 cm	20200810-1-(6)_0–40 cm_A_R2_SC4	CSF20881	*C. aconidialis*	AA--	L.L. Liu, J.L. Han & L.S. Sun	OK167786	OK168823	–	–
62	20–40 cm	20200810-1-(6)_0–40 cm_B_R1_SC1	CSF20882	*C. aconidialis*	AC--	L.L. Liu, J.L. Han & L.S. Sun	OK167895	OK168932	–	–
62	20–40 cm	20200810-1-(6)_0–40 cm_B_R1_SC2	CSF20883	*C. aconidialis*	AC--	L.L. Liu, J.L. Han & L.S. Sun	OK167896	OK168933	–	–
62	20–40 cm	20200810-1-(6)_0–40 cm_B_R1_SC3	CSF20884	*C. aconidialis*	AA--	L.L. Liu, J.L. Han & L.S. Sun	OK167787	OK168824	–	–
62	20–40 cm	20200810-1-(6)_0–40 cm_B_R1_SC4	CSF20885	*C. aconidialis*	AC--	L.L. Liu, J.L. Han & L.S. Sun	OK167897	OK168934	–	–
62	20–40 cm	20200810-1-(6)_0–40 cm_B_R2_SC1	CSF20886	*C. aconidialis*	AA--	L.L. Liu, J.L. Han & L.S. Sun	OK167788	OK168825	–	–
62	20–40 cm	20200810-1-(6)_0–40 cm_B_R2_SC2	CSF20887	*C. aconidialis*	AA--	L.L. Liu, J.L. Han & L.S. Sun	OK167789	OK168826	–	–
62	20–40 cm	20200810-1-(6)_0–40 cm_B_R2_SC3	CSF20888	*C. aconidialis*	AA--	L.L. Liu, J.L. Han & L.S. Sun	OK167790	OK168827	–	–
62	20–40 cm	20200810-1-(6)_0–40 cm_B_R2_SC4	CSF20889	*C. aconidialis*	AA--	L.L. Liu, J.L. Han & L.S. Sun	OK167791	OK168828	–	–
63	0–20 cm	20200810-1-(7)_0–20 cm_A_R1_SC1	CSF20890	*C. hongkongensis*	AA--	L.L. Liu, J.L. Han & L.S. Sun	OK167358	OK168395	–	–
63	0–20 cm	20200810-1-(7)_0–20 cm_A_R1_SC2	CSF20891	*C. hongkongensis*	DA--	L.L. Liu, J.L. Han & L.S. Sun	OK167693	OK168730	–	–
63	0–20 cm	20200810-1-(7)_0–20 cm_A_R2_SC1	CSF20892	*C. kyotensis*	AB--	L.L. Liu, J.L. Han & L.S. Sun	OK167993	OK169030	–	–

63	0–20 cm	20200810-1-(7)_0–20 cm_A_R2_SC2	CSF20893	*C. hongkongensis*	AA--	L.L. Liu, J.L. Han & L.S. Sun	OK167359	OK168396	–	–
63	0–20 cm	20200810-1-(7)_0–20 cm_B_R1_SC1	CSF20894	*C. hongkongensis*	AA--	L.L. Liu, J.L. Han & L.S. Sun	OK167360	OK168397	–	–
63	0–20 cm	20200810-1-(7)_0–20 cm_B_R1_SC2	CSF20895	*C. aconidialis*	AC--	L.L. Liu, J.L. Han & L.S. Sun	OK167898	OK168935	–	–
63	0–20 cm	20200810-1-(7)_0–20 cm_B_R2_SC1	CSF20896	*C. hongkongensis*	AA--	L.L. Liu, J.L. Han & L.S. Sun	OK167361	OK168398	–	–
63	0–20 cm	20200810-1-(7)_0–20 cm_B_R2_SC2	CSF20897	*C. hongkongensis*	AA--	L.L. Liu, J.L. Han & L.S. Sun	OK167362	OK168399	–	–
63	20–40 cm	20200810-1-(7)_0–40 cm_B_R1_SC1	CSF20898	*C. hongkongensis*	AA--	L.L. Liu, J.L. Han & L.S. Sun	OK167363	OK168400	–	–
63	20–40 cm	20200810-1-(7)_0–40 cm_B_R1_SC2	CSF20899	*C. hongkongensis*	AA--	L.L. Liu, J.L. Han & L.S. Sun	OK167364	OK168401	–	–
63	20–40 cm	20200810-1-(7)_0–40 cm_B_R1_SC3	CSF20900	*C. hongkongensis*	AA--	L.L. Liu, J.L. Han & L.S. Sun	OK167365	OK168402	–	–
63	20–40 cm	20200810-1-(7)_0–40 cm_B_R1_SC4	CSF20901	*C. hongkongensis*	AA--	L.L. Liu, J.L. Han & L.S. Sun	OK167366	OK168403	–	–
64	0–20 cm	20200810-1-(8)_0–20 cm_A_R1_SC1	CSF20910	*C. aconidialis*	AA--	L.L. Liu, J.L. Han & L.S. Sun	OK167792	OK168829	–	–
64	0–20 cm	20200810-1-(8)_0–20 cm_A_R1_SC2	CSF20911	*C. aconidialis*	AC--	L.L. Liu, J.L. Han & L.S. Sun	OK167899	OK168936	–	–
64	0–20 cm	20200810-1-(8)_0–20 cm_A_R2_SC1	CSF20912	*C. aconidialis*	AA--	L.L. Liu, J.L. Han & L.S. Sun	OK167793	OK168830	–	–
64	0–20 cm	20200810-1-(8)_0–20 cm_A_R2_SC2	CSF20913	*C. aconidialis*	AC--	L.L. Liu, J.L. Han & L.S. Sun	OK167900	OK168937	–	–
64	0–20 cm	20200810-1-(8)_0–20 cm_B_R1_SC1	CSF20914	*C. aconidialis*	AA--	L.L. Liu, J.L. Han & L.S. Sun	OK167794	OK168831	–	–
64	0–20 cm	20200810-1-(8)_0–20 cm_B_R1_SC2	CSF20915	*C. aconidialis*	AA--	L.L. Liu, J.L. Han & L.S. Sun	OK167795	OK168832	–	–
64	0–20 cm	20200810-1-(8)_0–20 cm_B_R2_SC1	CSF20916	*C. aconidialis*	AA--	L.L. Liu, J.L. Han & L.S. Sun	OK167796	OK168833	–	–
64	0–20 cm	20200810-1-(8)_0–20 cm_B_R2_SC2	CSF20917	*C. aconidialis*	AA--	L.L. Liu, J.L. Han & L.S. Sun	OK167797	OK168834	–	–

64	20–40 cm	20200810-1-(8)_0–40 cm_B_R2_SC4	CSF20918	*C. aconidialis*	AC--	L.L. Liu, J.L. Han & L.S. Sun	OK167901	OK168938	–	–
64	40–60 cm	20200810-1-(8)_0–60 cm_B_R2_SC1	CSF20919	*C. hongkongensis*	AA--	L.L. Liu, J.L. Han & L.S. Sun	OK167367	OK168404	–	–
64	40–60 cm	20200810-1-(8)_0–60 cm_B_R2_SC2	CSF20920	*C. hongkongensis*	AA--	L.L. Liu, J.L. Han & L.S. Sun	OK167368	OK168405	–	–
64	40–60 cm	20200810-1-(8)_0–60 cm_B_R2_SC3	CSF20921	*C. hongkongensis*	AA--	L.L. Liu, J.L. Han & L.S. Sun	OK167369	OK168406	–	–
64	40–60 cm	20200810-1-(8)_0–60 cm_B_R2_SC4	CSF20922	*C. hongkongensis*	AA--	L.L. Liu, J.L. Han & L.S. Sun	OK167370	OK168407	–	–
65	0–20 cm	20200811-1-(1)_0–20 cm_A_R1_SC1	CSF20923	*C. hongkongensis*	AEAA	L.L. Liu, J.L. Han & L.S. Sun	OK167608	OK168645	OK169122	OK169206
65	0–20 cm	20200811-1-(1)_0–20 cm_A_R1_SC2	CSF20924	*C. hongkongensis*	AEAA	L.L. Liu, J.L. Han & L.S. Sun	OK167609	OK168646	OK169123	OK169207
65	0–20 cm	20200811-1-(1)_0–20 cm_A_R2_SC1	CSF20925	*C. hongkongensis*	AA--	L.L. Liu, J.L. Han & L.S. Sun	OK167371	OK168408	–	–
65	0–20 cm	20200811-1-(1)_0–20 cm_A_R2_SC2	CSF20926	*C. hongkongensis*	AA--	L.L. Liu, J.L. Han & L.S. Sun	OK167372	OK168409	–	–
66	0–20 cm	20200811-1-(2)_0–20 cm_A_R1_SC1	CSF20927	*C. kyotensis*	AA--	L.L. Liu, J.L. Han & L.S. Sun	OK167958	OK168995	–	–
66	0–20 cm	20200811-1-(2)_0–20 cm_A_R1_SC2	CSF20928	*C. hongkongensis*	AD--	L.L. Liu, J.L. Han & L.S. Sun	OK167606	OK168643	–	–
66	0–20 cm	20200811-1-(2)_0–20 cm_A_R2_SC2	CSF20930	*C. kyotensis*	AA--	L.L. Liu, J.L. Han & L.S. Sun	OK167959	OK168996	–	–
66	0–20 cm	20200811-1-(2)_0–20 cm_B_R1_SC1	CSF20931	*C. hongkongensis*	AA--	L.L. Liu, J.L. Han & L.S. Sun	OK167373	OK168410	–	–
66	0–20 cm	20200811-1-(2)_0–20 cm_B_R1_SC2	CSF20932	*C. hongkongensis*	AA--	L.L. Liu, J.L. Han & L.S. Sun	OK167374	OK168411	–	–
66	0–20 cm	20200811-1-(2)_0–20 cm_B_R2_SC1	CSF20933	*C. hongkongensis*	AA--	L.L. Liu, J.L. Han & L.S. Sun	OK167375	OK168412	–	–
66	0–20 cm	20200811-1-(2)_0–20 cm_B_R2_SC2	CSF20934	*C. kyotensis*	AA--	L.L. Liu, J.L. Han & L.S. Sun	OK167960	OK168997	–	–
67	0–20 cm	20200811-1-(3)_0–20 cm_A_R1_SC1	CSF20935	*C. aconidialis*	AC--	L.L. Liu, J.L. Han & L.S. Sun	OK167902	OK168939	–	–

67	0–20 cm	20200811-1-(3)_0–20 cm_A_R1_SC2	CSF20936	*C. aconidialis*	AC--	L.L. Liu, J.L. Han & L.S. Sun	OK167903	OK168940	–	–
67	0–20 cm	20200811-1-(3)_0–20 cm_A_R2_SC1	CSF20937	*C. aconidialis*	AC--	L.L. Liu, J.L. Han & L.S. Sun	OK167904	OK168941	–	–
67	0–20 cm	20200811-1-(3)_0–20 cm_A_R2_SC2	CSF20938	*C. aconidialis*	AC--	L.L. Liu, J.L. Han & L.S. Sun	OK167905	OK168942	–	–
67	0–20 cm	20200811-1-(3)_0–20 cm_B_R1_SC1	CSF20939	*C. aconidialis*	AC--	L.L. Liu, J.L. Han & L.S. Sun	OK167906	OK168943	–	–
67	0–20 cm	20200811-1-(3)_0–20 cm_B_R1_SC2	CSF20940	*C. aconidialis*	AC--	L.L. Liu, J.L. Han & L.S. Sun	OK167907	OK168944	–	–
67	0–20 cm	20200811-1-(3)_0–20 cm_B_R2_SC1	CSF20941	*C. aconidialis*	AA--	L.L. Liu, J.L. Han & L.S. Sun	OK167798	OK168835	–	–
67	0–20 cm	20200811-1-(3)_0–20 cm_B_R2_SC2	CSF20942	*C. aconidialis*	AC--	L.L. Liu, J.L. Han & L.S. Sun	OK167908	OK168945	–	–
67	20–40 cm	20200811-1-(3)_0–40 cm_A_R1_SC1	CSF20943	*C. aconidialis*	AC--	L.L. Liu, J.L. Han & L.S. Sun	OK167909	OK168946	–	–
67	20–40 cm	20200811-1-(3)_0–40 cm_A_R1_SC2	CSF20944	*C. aconidialis*	AC--	L.L. Liu, J.L. Han & L.S. Sun	OK167910	OK168947	–	–
67	20–40 cm	20200811-1-(3)_0–40 cm_A_R1_SC3	CSF20945	*C. aconidialis*	AC--	L.L. Liu, J.L. Han & L.S. Sun	OK167911	OK168948	–	–
67	20–40 cm	20200811-1-(3)_0–40 cm_A_R1_SC4	CSF20946	*C. aconidialis*	AC--	L.L. Liu, J.L. Han & L.S. Sun	OK167912	OK168949	–	–
67	20–40 cm	20200811-1-(3)_0–40 cm_A_R2_SC1	CSF20947	*C. aconidialis*	AC--	L.L. Liu, J.L. Han & L.S. Sun	OK167913	OK168950	–	–
67	20–40 cm	20200811-1-(3)_0–40 cm_A_R2_SC2	CSF20948	*C. aconidialis*	AC--	L.L. Liu, J.L. Han & L.S. Sun	OK167914	OK168951	–	–
67	20–40 cm	20200811-1-(3)_0–40 cm_A_R2_SC3	CSF20949	*C. aconidialis*	AC--	L.L. Liu, J.L. Han & L.S. Sun	OK167915	OK168952	–	–
67	20–40 cm	20200811-1-(3)_0–40 cm_A_R2_SC4	CSF20950	*C. aconidialis*	AC--	L.L. Liu, J.L. Han & L.S. Sun	OK167916	OK168953	–	–
67	20–40 cm	20200811-1-(3)_0–40 cm_B_R1_SC1	CSF20951	*C. aconidialis*	AC--	L.L. Liu, J.L. Han & L.S. Sun	OK167917	OK168954	–	–
67	20–40 cm	20200811-1-(3)_0–40 cm_B_R1_SC2	CSF20952	*C. aconidialis*	AC--	L.L. Liu, J.L. Han & L.S. Sun	OK167918	OK168955	–	–

67	20–40 cm	20200811-1-(3)_0–40 cm_B_R1_SC3	CSF20953	*C. aconidialis*	AC--	L.L. Liu, J.L. Han & L.S. Sun	OK167919	OK168956	–	–
67	20–40 cm	20200811-1-(3)_0–40 cm_B_R1_SC4	CSF20954	*C. aconidialis*	AC--	L.L. Liu, J.L. Han & L.S. Sun	OK167920	OK168957	–	–
67	20–40 cm	20200811-1-(3)_0–40 cm_B_R2_SC1	CSF20955	*C. aconidialis*	AC--	L.L. Liu, J.L. Han & L.S. Sun	OK167921	OK168958	–	–
67	20–40 cm	20200811-1-(3)_0–40 cm_B_R2_SC2	CSF20956	*C. aconidialis*	AC--	L.L. Liu, J.L. Han & L.S. Sun	OK167922	OK168959	–	–
67	20–40 cm	20200811-1-(3)_0–40 cm_B_R2_SC3	CSF20957	*C. aconidialis*	AC--	L.L. Liu, J.L. Han & L.S. Sun	OK167923	OK168960	–	–
67	20–40 cm	20200811-1-(3)_0–40 cm_B_R2_SC4	CSF20958	*C. aconidialis*	AC--	L.L. Liu, J.L. Han & L.S. Sun	OK167924	OK168961	–	–
67	40–60 cm	20200811-1-(3)_0–60 cm_A_R1_SC1	CSF20959	*C. aconidialis*	AC--	L.L. Liu, J.L. Han & L.S. Sun	OK167925	OK168962	–	–
67	40–60 cm	20200811-1-(3)_0–60 cm_A_R1_SC2	CSF20960	*C. aconidialis*	AC--	L.L. Liu, J.L. Han & L.S. Sun	OK167926	OK168963	–	–
67	40–60 cm	20200811-1-(3)_0–60 cm_A_R1_SC3	CSF20961	*C. aconidialis*	AC--	L.L. Liu, J.L. Han & L.S. Sun	OK167927	OK168964	–	–
67	40–60 cm	20200811-1-(3)_0–60 cm_A_R1_SC4	CSF20962	*C. aconidialis*	AC--	L.L. Liu, J.L. Han & L.S. Sun	OK167928	OK168965	–	–
67	40–60 cm	20200811-1-(3)_0–60 cm_A_R2_SC1	CSF20963	*C. aconidialis*	AC--	L.L. Liu, J.L. Han & L.S. Sun	OK167929	OK168966	–	–
67	40–60 cm	20200811-1-(3)_0–60 cm_A_R2_SC2	CSF20964	*C. aconidialis*	AC--	L.L. Liu, J.L. Han & L.S. Sun	OK167930	OK168967	–	–
67	40–60 cm	20200811-1-(3)_0–60 cm_A_R2_SC3	CSF20965	*C. aconidialis*	AC--	L.L. Liu, J.L. Han & L.S. Sun	OK167931	OK168968	–	–
67	40–60 cm	20200811-1-(3)_0–60 cm_A_R2_SC4	CSF20966	*C. aconidialis*	AC--	L.L. Liu, J.L. Han & L.S. Sun	OK167932	OK168969	–	–
67	40–60 cm	20200811-1-(3)_0–60 cm_B_R2_SC1	CSF20967	*C. aconidialis*	AC--	L.L. Liu, J.L. Han & L.S. Sun	OK167933	OK168970	–	–
67	40–60 cm	20200811-1-(3)_0–60 cm_B_R2_SC2	CSF20968	*C. aconidialis*	AC--	L.L. Liu, J.L. Han & L.S. Sun	OK167934	OK168971	–	–
67	40–60 cm	20200811-1-(3)_0–60 cm_B_R2_SC3	CSF20969	*C. aconidialis*	AC--	L.L. Liu, J.L. Han & L.S. Sun	OK167935	OK168972	–	–

67	40–60 cm	20200811-1-(3)_0–60 cm_B_R2_SC4	CSF20970	*C. aconidialis*	AC--	L.L. Liu, J.L. Han & L.S. Sun	OK167936	OK168973	–	–
68	0–20 cm	20200811-1-(4)_0–20 cm_A_R2_SC1	CSF20971	*C. aconidialis*	AA--	L.L. Liu, J.L. Han & L.S. Sun	OK167799	OK168836	–	–
68	0–20 cm	20200811-1-(4)_0–20 cm_A_R2_SC2	CSF20972	*C. aconidialis*	AA--	L.L. Liu, J.L. Han & L.S. Sun	OK167800	OK168837	–	–
68	0–20 cm	20200811-1-(4)_0–20 cm_B_R1_SC1	CSF20973	*C. hongkongensis*	AA--	L.L. Liu, J.L. Han & L.S. Sun	OK167376	OK168413	–	–
68	0–20 cm	20200811-1-(4)_0–20 cm_B_R1_SC2	CSF20974	*C. hongkongensis*	AA--	L.L. Liu, J.L. Han & L.S. Sun	OK167377	OK168414	–	–
68	20–40 cm	20200811-1-(4)_0–40 cm_A_R1_SC1	CSF20975	*C. aconidialis*	AA--	L.L. Liu, J.L. Han & L.S. Sun	OK167801	OK168838	–	–
68	20–40 cm	20200811-1-(4)_0–40 cm_A_R1_SC2	CSF20976	*C. aconidialis*	AA--	L.L. Liu, J.L. Han & L.S. Sun	OK167802	OK168839	–	–
68	20–40 cm	20200811-1-(4)_0–40 cm_A_R1_SC3	CSF20977	*C. aconidialis*	AA--	L.L. Liu, J.L. Han & L.S. Sun	OK167803	OK168840	–	–
68	20–40 cm	20200811-1-(4)_0–40 cm_A_R1_SC4	CSF20978	*C. aconidialis*	AA--	L.L. Liu, J.L. Han & L.S. Sun	OK167804	OK168841	–	–
68	20–40 cm	20200811-1-(4)_0–40 cm_A_R2_SC1	CSF20979	*C. aconidialis*	AA--	L.L. Liu, J.L. Han & L.S. Sun	OK167805	OK168842	–	–
68	20–40 cm	20200811-1-(4)_0–40 cm_A_R2_SC2	CSF20980	*C. aconidialis*	AA--	L.L. Liu, J.L. Han & L.S. Sun	OK167806	OK168843	–	–
68	20–40 cm	20200811-1-(4)_0–40 cm_A_R2_SC3	CSF20981	*C. aconidialis*	AA--	L.L. Liu, J.L. Han & L.S. Sun	OK167807	OK168844	–	–
68	20–40 cm	20200811-1-(4)_0–40 cm_A_R2_SC4	CSF20982	*C. aconidialis*	AA--	L.L. Liu, J.L. Han & L.S. Sun	OK167808	OK168845	–	–
68	20–40 cm	20200811-1-(4)_0–40 cm_B_R1_SC1	CSF20983	*C. aconidialis*	AA--	L.L. Liu, J.L. Han & L.S. Sun	OK167809	OK168846	–	–
68	20–40 cm	20200811-1-(4)_0–40 cm_B_R1_SC2	CSF20984	*C. aconidialis*	AA--	L.L. Liu, J.L. Han & L.S. Sun	OK167810	OK168847	–	–
68	20–40 cm	20200811-1-(4)_0–40 cm_B_R1_SC3	CSF20985	*C. aconidialis*	ABBA	L.L. Liu, J.L. Han & L.S. Sun	OK167856	OK168893	OK169152	OK169236
68	20–40 cm	20200811-1-(4)_0–40 cm_B_R2_SC1	CSF20987	*C. aconidialis*	AA--	L.L. Liu, J.L. Han & L.S. Sun	OK167811	OK168848	–	–

68	20–40 cm	20200811-1-(4)_0–40 cm_B_R2_SC2	CSF20988	*C. aconidialis*	AA--	L.L. Liu, J.L. Han & L.S. Sun	OK167812	OK168849	–	–
68	20–40 cm	20200811-1-(4)_0–40 cm_B_R2_SC3	CSF20989	*C. aconidialis*	AA--	L.L. Liu, J.L. Han & L.S. Sun	OK167813	OK168850	–	–
68	20–40 cm	20200811-1-(4)_0–40 cm_B_R2_SC4	CSF20990	*C. aconidialis*	AA--	L.L. Liu, J.L. Han & L.S. Sun	OK167814	OK168851	–	–
69	0–20 cm	20200811-1-(5)_0–20 cm_A_R1_SC1	CSF20991	*C. hongkongensis*	AA--	L.L. Liu, J.L. Han & L.S. Sun	OK167378	OK168415	–	–
69	0–20 cm	20200811-1-(5)_0–20 cm_A_R1_SC2	CSF20992	*C. kyotensis*	AA--	L.L. Liu, J.L. Han & L.S. Sun	OK167961	OK168998	–	–
69	0–20 cm	20200811-1-(5)_0–20 cm_B_R1_SC1	CSF20993	*C. hongkongensis*	AA--	L.L. Liu, J.L. Han & L.S. Sun	OK167379	OK168416	–	–
69	0–20 cm	20200811-1-(5)_0–20 cm_B_R1_SC2	CSF20994	*C. kyotensis*	AA--	L.L. Liu, J.L. Han & L.S. Sun	OK167962	OK168999	–	–
69	0–20 cm	20200811-1-(5)_0–20 cm_B_R2_SC1	CSF20995	*C. aconidialis*	AA--	L.L. Liu, J.L. Han & L.S. Sun	OK167815	OK168852	–	–
69	0–20 cm	20200811-1-(5)_0–20 cm_B_R2_SC2	CSF20996	*C. kyotensis*	AA--	L.L. Liu, J.L. Han & L.S. Sun	OK167963	OK169000	–	–
69	20–40 cm	20200811-1-(5)_0–40 cm_A_R1_SC1	CSF20997	*C. hongkongensis*	AA--	L.L. Liu, J.L. Han & L.S. Sun	OK167380	OK168417	–	–
69	20–40 cm	20200811-1-(5)_0–40 cm_A_R1_SC2	CSF20998	*C. hongkongensis*	AA--	L.L. Liu, J.L. Han & L.S. Sun	OK167381	OK168418	–	–
69	20–40 cm	20200811-1-(5)_0–40 cm_A_R1_SC3	CSF20999	*C. hongkongensis*	AA--	L.L. Liu, J.L. Han & L.S. Sun	OK167382	OK168419	–	–
69	20–40 cm	20200811-1-(5)_0–40 cm_A_R1_SC4	CSF21000	*C. hongkongensis*	AA--	L.L. Liu, J.L. Han & L.S. Sun	OK167383	OK168420	–	–
69	40–60 cm	20200811-1-(5)_0–60 cm_B_R2_SC1	CSF21001	*C. hongkongensis*	AA--	L.L. Liu, J.L. Han & L.S. Sun	OK167384	OK168421	–	–
69	40–60 cm	20200811-1-(5)_0–60 cm_B_R2_SC2	CSF21002	*C. hongkongensis*	AA--	L.L. Liu, J.L. Han & L.S. Sun	OK167385	OK168422	–	–
69	40–60 cm	20200811-1-(5)_0–60 cm_B_R2_SC3	CSF21003	*C. hongkongensis*	AA--	L.L. Liu, J.L. Han & L.S. Sun	OK167386	OK168423	–	–
69	40–60 cm	20200811-1-(5)_0–60 cm_B_R2_SC4	CSF21004	*C. hongkongensis*	AA--	L.L. Liu, J.L. Han & L.S. Sun	OK167387	OK168424	–	–

70	0–20 cm	20200811-1-(6)_0–20 cm_A_R2_SC1	CSF21005	*C. hongkongensis*	AA--	L.L. Liu, J.L. Han & L.S. Sun	OK167388	OK168425	–	–
70	0–20 cm	20200811-1-(6)_0–20 cm_A_R2_SC2	CSF21006	*C. hongkongensis*	AA--	L.L. Liu, J.L. Han & L.S. Sun	OK167389	OK168426	–	–
70	0–20 cm	20200811-1-(6)_0–20 cm_B_R1_SC2	CSF21007	*C. hongkongensis*	AA--	L.L. Liu, J.L. Han & L.S. Sun	OK167390	OK168427	–	–
70	0–20 cm	20200811-1-(6)_0–20 cm_B_R2_SC1	CSF21008	*C. aconidialis*	AA--	L.L. Liu, J.L. Han & L.S. Sun	OK167816	OK168853	–	–
70	0–20 cm	20200811-1-(6)_0–20 cm_B_R2_SC2	CSF21009	*C. aconidialis*	AA--	L.L. Liu, J.L. Han & L.S. Sun	OK167817	OK168854	–	–
70	20–40 cm	20200811-1-(6)_0–40 cm_A_R1_SC1	CSF21010	*C. hongkongensis*	AA--	L.L. Liu, J.L. Han & L.S. Sun	OK167391	OK168428	–	–
70	20–40 cm	20200811-1-(6)_0–40 cm_A_R1_SC2	CSF21011	*C. hongkongensis*	AA--	L.L. Liu, J.L. Han & L.S. Sun	OK167392	OK168429	–	–
70	20–40 cm	20200811-1-(6)_0–40 cm_A_R1_SC3	CSF21012	*C. hongkongensis*	AA--	L.L. Liu, J.L. Han & L.S. Sun	OK167393	OK168430	–	–
70	20–40 cm	20200811-1-(6)_0–40 cm_A_R1_SC4	CSF21013	*C. hongkongensis*	AA--	L.L. Liu, J.L. Han & L.S. Sun	OK167394	OK168431	–	–
71	0–20 cm	20200811-1-(7)_0–20 cm_A_R1_SC1	CSF21014	*C. hongkongensis*	AA--	L.L. Liu, J.L. Han & L.S. Sun	OK167395	OK168432	–	–
71	0–20 cm	20200811-1-(7)_0–20 cm_A_R1_SC2	CSF21015	*C. hongkongensis*	AA--	L.L. Liu, J.L. Han & L.S. Sun	OK167396	OK168433	–	–
71	0–20 cm	20200811-1-(7)_0–20 cm_A_R2_SC1	CSF21016	*C. hongkongensis*	AA--	L.L. Liu, J.L. Han & L.S. Sun	OK167397	OK168434	–	–
71	0–20 cm	20200811-1-(7)_0–20 cm_A_R2_SC2	CSF21017	*C. hongkongensis*	AA--	L.L. Liu, J.L. Han & L.S. Sun	OK167398	OK168435	–	–
71	0–20 cm	20200811-1-(7)_0–20 cm_B_R1_SC1	CSF21018	*C. hongkongensis*	AGAA	L.L. Liu, J.L. Han & L.S. Sun	OK167644	OK168681	OK169131	OK169215
71	0–20 cm	20200811-1-(7)_0–20 cm_B_R1_SC2	CSF21019	*C. hongkongensis*	AA--	L.L. Liu, J.L. Han & L.S. Sun	OK167399	OK168436	–	–
71	0–20 cm	20200811-1-(7)_0–20 cm_B_R2_SC1	CSF21020	*C. hongkongensis*	AA--	L.L. Liu, J.L. Han & L.S. Sun	OK167400	OK168437	–	–
71	0–20 cm	20200811-1-(7)_0–20 cm_B_R2_SC2	CSF21021	*C. hongkongensis*	AA--	L.L. Liu, J.L. Han & L.S. Sun	OK167401	OK168438	–	–

71	20–40 cm	20200811-1-(7)_0–40 cm_A_R1_SC1	CSF21022	*C. hongkongensis*	AA--	L.L. Liu, J.L. Han & L.S. Sun	OK167402	OK168439	–	–
71	20–40 cm	20200811-1-(7)_0–40 cm_A_R1_SC2	CSF21023	*C. hongkongensis*	AA--	L.L. Liu, J.L. Han & L.S. Sun	OK167403	OK168440	–	–
71	20–40 cm	20200811-1-(7)_0–40 cm_A_R1_SC3	CSF21024	*C. hongkongensis*	AA--	L.L. Liu, J.L. Han & L.S. Sun	OK167404	OK168441	–	–
71	20–40 cm	20200811-1-(7)_0–40 cm_A_R1_SC4	CSF21025	*C. hongkongensis*	AA--	L.L. Liu, J.L. Han & L.S. Sun	OK167405	OK168442	–	–
71	20–40 cm	20200811-1-(7)_0–40 cm_A_R2_SC1	CSF21026	*C. hongkongensis*	AA--	L.L. Liu, J.L. Han & L.S. Sun	OK167406	OK168443	–	–
71	20–40 cm	20200811-1-(7)_0–40 cm_A_R2_SC2	CSF21027	*C. hongkongensis*	AA--	L.L. Liu, J.L. Han & L.S. Sun	OK167407	OK168444	–	–
71	20–40 cm	20200811-1-(7)_0–40 cm_A_R2_SC3	CSF21028	*C. hongkongensis*	AA--	L.L. Liu, J.L. Han & L.S. Sun	OK167408	OK168445	–	–
71	20–40 cm	20200811-1-(7)_0–40 cm_A_R2_SC4	CSF21029	*C. hongkongensis*	AA--	L.L. Liu, J.L. Han & L.S. Sun	OK167409	OK168446	–	–
71	20–40 cm	20200811-1-(7)_0–40 cm_B_R1_SC1	CSF21030	*C. hongkongensis*	AA--	L.L. Liu, J.L. Han & L.S. Sun	OK167410	OK168447	–	–
71	20–40 cm	20200811-1-(7)_0–40 cm_B_R1_SC2	CSF21031	*C. hongkongensis*	AA--	L.L. Liu, J.L. Han & L.S. Sun	OK167411	OK168448	–	–
71	20–40 cm	20200811-1-(7)_0–40 cm_B_R1_SC3	CSF21032	*C. hongkongensis*	AA--	L.L. Liu, J.L. Han & L.S. Sun	OK167412	OK168449	–	–
71	20–40 cm	20200811-1-(7)_0–40 cm_B_R1_SC4	CSF21033	*C. hongkongensis*	AA--	L.L. Liu, J.L. Han & L.S. Sun	OK167413	OK168450	–	–
72	0–20 cm	20200811-1-(8)_0–20 cm_A_R1_SC1	CSF21034	*C. hongkongensis*	AA--	L.L. Liu, J.L. Han & L.S. Sun	OK167414	OK168451	–	–
72	0–20 cm	20200811-1-(8)_0–20 cm_A_R1_SC2	CSF21035	*C. hongkongensis*	AA--	L.L. Liu, J.L. Han & L.S. Sun	OK167415	OK168452	–	–
72	20–40 cm	20200811-1-(8)_0–40 cm_A_R1_SC1	CSF21036	*C. kyotensis*	AA--	L.L. Liu, J.L. Han & L.S. Sun	OK167964	OK169001	–	–
72	20–40 cm	20200811-1-(8)_0–40 cm_A_R1_SC2	CSF21037	*C. kyotensis*	AA--	L.L. Liu, J.L. Han & L.S. Sun	OK167965	OK169002	–	–
72	20–40 cm	20200811-1-(8)_0–40 cm_A_R1_SC3	CSF21038	*C. kyotensis*	AA--	L.L. Liu, J.L. Han & L.S. Sun	OK167966	OK169003	–	–

72	20–40 cm	20200811-1-(8)_0–40 cm_A_R1_SC4	CSF21039	*C. kyotensis*	AA--	L.L. Liu, J.L. Han & L.S. Sun	OK167967	OK169004	–	–
72	20–40 cm	20200811-1-(8)_0–40 cm_B_R2_SC1	CSF21040	*C. hongkongensis*	AA--	L.L. Liu, J.L. Han & L.S. Sun	OK167416	OK168453	–	–
72	20–40 cm	20200811-1-(8)_0–40 cm_B_R2_SC2	CSF21041	*C. hongkongensis*	AA--	L.L. Liu, J.L. Han & L.S. Sun	OK167417	OK168454	–	–
72	20–40 cm	20200811-1-(8)_0–40 cm_B_R2_SC3	CSF21042	*C. hongkongensis*	AA--	L.L. Liu, J.L. Han & L.S. Sun	OK167418	OK168455	–	–
72	20–40 cm	20200811-1-(8)_0–40 cm_B_R2_SC4	CSF21043	*C. hongkongensis*	AA--	L.L. Liu, J.L. Han & L.S. Sun	OK167419	OK168456	–	–
73	0–20 cm	20200812-1-(1)_0–20 cm_A_R2_SC1	CSF21044	*C. aconidialis*	AC--	L.L. Liu, J.L. Han & L.S. Sun	OK167937	OK168974	–	–
73	0–20 cm	20200812-1-(1)_0–20 cm_A_R2_SC2	CSF21045	*C. aconidialis*	AC--	L.L. Liu, J.L. Han & L.S. Sun	OK167938	OK168975	–	–
73	0–20 cm	20200812-1-(1)_0–20 cm_B_R1_SC1	CSF21046	*C. hongkongensis*	AA--	L.L. Liu, J.L. Han & L.S. Sun	OK167420	OK168457	–	–
73	0–20 cm	20200812-1-(1)_0–20 cm_B_R1_SC2	CSF21047	*C. hongkongensis*	AA--	L.L. Liu, J.L. Han & L.S. Sun	OK167421	OK168458	–	–
73	20–40 cm	20200812-1-(1)_0–40 cm_A_R2_SC1	CSF21048	*C. hongkongensis*	AA--	L.L. Liu, J.L. Han & L.S. Sun	OK167422	OK168459	–	–
73	20–40 cm	20200812-1-(1)_0–40 cm_A_R2_SC2	CSF21049	*C. hongkongensis*	AA--	L.L. Liu, J.L. Han & L.S. Sun	OK167423	OK168460	–	–
73	20–40 cm	20200812-1-(1)_0–40 cm_A_R2_SC3	CSF21050	*C. hongkongensis*	AA--	L.L. Liu, J.L. Han & L.S. Sun	OK167424	OK168461	–	–
73	20–40 cm	20200812-1-(1)_0–40 cm_A_R2_SC4	CSF21051	*C. hongkongensis*	AA--	L.L. Liu, J.L. Han & L.S. Sun	OK167425	OK168462	–	–
74	0–20 cm	20200812-1-(2)_0–20 cm_A_R1_SC1	CSF21052	*C. ilicicola*	BBBA	L.L. Liu, J.L. Han & L.S. Sun	OK168044	OK169081	OK169181	OK169265
74	0–20 cm	20200812-1-(2)_0–20 cm_A_R1_SC2	CSF21053	*C. hongkongensis*	AA--	L.L. Liu, J.L. Han & L.S. Sun	OK167426	OK168463	–	–
74	0–20 cm	20200812-1-(2)_0–20 cm_A_R2_SC1	CSF21054	*C. hongkongensis*	AA--	L.L. Liu, J.L. Han & L.S. Sun	OK167427	OK168464	–	–
74	0–20 cm	20200812-1-(2)_0–20 cm_A_R2_SC2	CSF21055	*C. ilicicola*	BB--	L.L. Liu, J.L. Han & L.S. Sun	OK168045	OK169082	–	–

74	0–20 cm	20200812-1-(2)_0–20 cm_B_R1_SC1	CSF21056	*C. ilicicola*	BB--	L.L. Liu, J.L. Han & L.S. Sun	OK168046	OK169083	–	–
74	0–20 cm	20200812-1-(2)_0–20 cm_B_R1_SC2	CSF21057	*C. hongkongensis*	AA--	L.L. Liu, J.L. Han & L.S. Sun	OK167428	OK168465	–	–
74	0–20 cm	20200812-1-(2)_0–20 cm_B_R2_SC1	CSF21058	*C. hongkongensis*	AA--	L.L. Liu, J.L. Han & L.S. Sun	OK167429	OK168466	–	–
74	0–20 cm	20200812-1-(2)_0–20 cm_B_R2_SC2	CSF21059	*C. hongkongensis*	AA--	L.L. Liu, J.L. Han & L.S. Sun	OK167430	OK168467	–	–
75	0–20 cm	20200812-1-(3)_0–20 cm_A_R1_SC1	CSF21060	*C. kyotensis*	AA--	L.L. Liu, J.L. Han & L.S. Sun	OK167968	OK169005	–	–
75	0–20 cm	20200812-1-(3)_0–20 cm_A_R1_SC2	CSF21061	*C. kyotensis*	AA--	L.L. Liu, J.L. Han & L.S. Sun	OK167969	OK169006	–	–
75	0–20 cm	20200812-1-(3)_0–20 cm_A_R2_SC1	CSF21062	*C. kyotensis*	AA--	L.L. Liu, J.L. Han & L.S. Sun	OK167970	OK169007	–	–
75	0–20 cm	20200812-1-(3)_0–20 cm_A_R2_SC2	CSF21063	*C. hongkongensis*	AA--	L.L. Liu, J.L. Han & L.S. Sun	OK167431	OK168468	–	–
75	0–20 cm	20200812-1-(3)_0–20 cm_B_R1_SC1	CSF21064	*C. kyotensis*	AA--	L.L. Liu, J.L. Han & L.S. Sun	OK167971	OK169008	–	–
75	0–20 cm	20200812-1-(3)_0–20 cm_B_R1_SC2	CSF21065	*C. kyotensis*	AA--	L.L. Liu, J.L. Han & L.S. Sun	OK167972	OK169009	–	–
75	0–20 cm	20200812-1-(3)_0–20 cm_B_R2_SC1	CSF21066	*C. hongkongensis*	AA--	L.L. Liu, J.L. Han & L.S. Sun	OK167432	OK168469	–	–
75	0–20 cm	20200812-1-(3)_0–20 cm_B_R2_SC2	CSF21067	*C. hongkongensis*	AA--	L.L. Liu, J.L. Han & L.S. Sun	OK167433	OK168470	–	–
75	60–80 cm	20200812-1-(3)_0–80 cm_A_R2_SC1	CSF21068	*C. kyotensis*	AB--	L.L. Liu, J.L. Han & L.S. Sun	OK167994	OK169031	–	–
75	60–80 cm	20200812-1-(3)_0–80 cm_A_R2_SC2	CSF21069	*C. kyotensis*	AB--	L.L. Liu, J.L. Han & L.S. Sun	OK167995	OK169032	–	–
75	60–80 cm	20200812-1-(3)_0–80 cm_A_R2_SC3	CSF21070	*C. kyotensis*	AB--	L.L. Liu, J.L. Han & L.S. Sun	OK167996	OK169033	–	–
75	60–80 cm	20200812-1-(3)_0–80 cm_A_R2_SC4	CSF21071	*C. kyotensis*	AB--	L.L. Liu, J.L. Han & L.S. Sun	OK167997	OK169034	–	–
76	0–20 cm	20200812-1-(4)_0–20 cm_A_R1_SC1	CSF21076	*C. hongkongensis*	AA--	L.L. Liu, J.L. Han & L.S. Sun	OK167434	OK168471	–	–

76	0–20 cm	20200812-1-(4)_0–20 cm_A_R1_SC2	CSF21077	*C. hongkongensis*	AA--	L.L. Liu, J.L. Han & L.S. Sun	OK167435	OK168472	–	–
76	0–20 cm	20200812-1-(4)_0–20 cm_A_R2_SC1	CSF21078	*C. hongkongensis*	AA--	L.L. Liu, J.L. Han & L.S. Sun	OK167436	OK168473	–	–
76	0–20 cm	20200812-1-(4)_0–20 cm_A_R2_SC2	CSF21079	*C. aconidialis*	AA--	L.L. Liu, J.L. Han & L.S. Sun	OK167818	OK168855	–	–
76	0–20 cm	20200812-1-(4)_0–20 cm_B_R2_SC1	CSF21080	*C. aconidialis*	AA--	L.L. Liu, J.L. Han & L.S. Sun	OK167819	OK168856	–	–
76	0–20 cm	20200812-1-(4)_0–20 cm_B_R2_SC2	CSF21081	*C. kyotensis*	AA--	L.L. Liu, J.L. Han & L.S. Sun	OK167973	OK169010	–	–
76	20–40 cm	20200812-1-(4)_0–40 cm_A_R1_SC1	CSF21082	*C. hongkongensis*	AA--	L.L. Liu, J.L. Han & L.S. Sun	OK167437	OK168474	–	–
76	20–40 cm	20200812-1-(4)_0–40 cm_A_R1_SC2	CSF21083	*C. hongkongensis*	AA--	L.L. Liu, J.L. Han & L.S. Sun	OK167438	OK168475	–	–
76	20–40 cm	20200812-1-(4)_0–40 cm_A_R1_SC3	CSF21084	*C. hongkongensis*	AA--	L.L. Liu, J.L. Han & L.S. Sun	OK167439	OK168476	–	–
76	20–40 cm	20200812-1-(4)_0–40 cm_A_R1_SC4	CSF21085	*C. hongkongensis*	AA--	L.L. Liu, J.L. Han & L.S. Sun	OK167440	OK168477	–	–
76	20–40 cm	20200812-1-(4)_0–40 cm_B_R2_SC1	CSF21086	*C. hongkongensis*	AA--	L.L. Liu, J.L. Han & L.S. Sun	OK167441	OK168478	–	–
76	20–40 cm	20200812-1-(4)_0–40 cm_B_R2_SC2	CSF21087	*C. hongkongensis*	AA--	L.L. Liu, J.L. Han & L.S. Sun	OK167442	OK168479	–	–
76	20–40 cm	20200812-1-(4)_0–40 cm_B_R2_SC3	CSF21088	*C. hongkongensis*	AA--	L.L. Liu, J.L. Han & L.S. Sun	OK167443	OK168480	–	–
76	20–40 cm	20200812-1-(4)_0–40 cm_B_R2_SC4	CSF21089	*C. hongkongensis*	AA--	L.L. Liu, J.L. Han & L.S. Sun	OK167444	OK168481	–	–
76	40–60 cm	20200812-1-(4)_0–60 cm_A_R2_SC1	CSF21090	*C. hongkongensis*	AF--	L.L. Liu, J.L. Han & L.S. Sun	OK167622	OK168659	–	–
76	40–60 cm	20200812-1-(4)_0–60 cm_A_R2_SC2	CSF21091	*C. hongkongensis*	AF--	L.L. Liu, J.L. Han & L.S. Sun	OK167623	OK168660	–	–
76	40–60 cm	20200812-1-(4)_0–60 cm_A_R2_SC3	CSF21092	*C. hongkongensis*	AF--	L.L. Liu, J.L. Han & L.S. Sun	OK167624	OK168661	–	–
77	0–20 cm	20200812-1-(5)_0–20 cm_A_R1_SC1	CSF21093	*C. aconidialis*	AA--	L.L. Liu, J.L. Han & L.S. Sun	OK167820	OK168857	–	–

77	0–20 cm	20200812-1-(5)_0–20 cm_A_R1_SC2	CSF21094	*C. aconidialis*	AA--	L.L. Liu, J.L. Han & L.S. Sun	OK167821	OK168858	–	–
77	0–20 cm	20200812-1-(5)_0–20 cm_A_R2_SC1	CSF21095	*C. aconidialis*	AA--	L.L. Liu, J.L. Han & L.S. Sun	OK167822	OK168859	–	–
77	0–20 cm	20200812-1-(5)_0–20 cm_A_R2_SC2	CSF21096	*C. aconidialis*	AA--	L.L. Liu, J.L. Han & L.S. Sun	OK167823	OK168860	–	–
77	0–20 cm	20200812-1-(5)_0–20 cm_B_R1_SC1	CSF21097	*C. aconidialis*	AA--	L.L. Liu, J.L. Han & L.S. Sun	OK167824	OK168861	–	–
77	0–20 cm	20200812-1-(5)_0–20 cm_B_R1_SC2	CSF21098	*C. hongkongensis*	AA--	L.L. Liu, J.L. Han & L.S. Sun	OK167445	OK168482	–	–
77	0–20 cm	20200812-1-(5)_0–20 cm_B_R2_SC1	CSF21099	*C. hongkongensis*	AA--	L.L. Liu, J.L. Han & L.S. Sun	OK167446	OK168483	–	–
77	0–20 cm	20200812-1-(5)_0–20 cm_B_R2_SC2	CSF21100	*C. aconidialis*	AA--	L.L. Liu, J.L. Han & L.S. Sun	OK167825	OK168862	–	–
77	20–40 cm	20200812-1-(5)_0–40 cm_B_R1_SC1	CSF21101	*C. hongkongensis*	AA--	L.L. Liu, J.L. Han & L.S. Sun	OK167447	OK168484	–	–
77	20–40 cm	20200812-1-(5)_0–40 cm_B_R1_SC2	CSF21102	*C. hongkongensis*	AA--	L.L. Liu, J.L. Han & L.S. Sun	OK167448	OK168485	–	–
77	20–40 cm	20200812-1-(5)_0–40 cm_B_R1_SC3	CSF21103	*C. hongkongensis*	AA--	L.L. Liu, J.L. Han & L.S. Sun	OK167449	OK168486	–	–
77	20–40 cm	20200812-1-(5)_0–40 cm_B_R1_SC4	CSF21104	*C. hongkongensis*	AA--	L.L. Liu, J.L. Han & L.S. Sun	OK167450	OK168487	–	–
78	0–20 cm	20200812-1-(6)_0–20 cm_A_R1_SC1	CSF21105	*C. hongkongensis*	AA--	L.L. Liu, J.L. Han & L.S. Sun	OK167451	OK168488	–	–
78	0–20 cm	20200812-1-(6)_0–20 cm_A_R1_SC2	CSF21106	*C. hongkongensis*	AA--	L.L. Liu, J.L. Han & L.S. Sun	OK167452	OK168489	–	–
78	0–20 cm	20200812-1-(6)_0–20 cm_A_R2_SC1	CSF21107	*C. aconidialis*	AA--	L.L. Liu, J.L. Han & L.S. Sun	OK167826	OK168863	–	–
78	0–20 cm	20200812-1-(6)_0–20 cm_A_R2_SC2	CSF21108	*C. aconidialis*	AA--	L.L. Liu, J.L. Han & L.S. Sun	OK167827	OK168864	–	–
78	0–20 cm	20200812-1-(6)_0–20 cm_B_R1_SC1	CSF21109	*C. aconidialis*	AA--	L.L. Liu, J.L. Han & L.S. Sun	OK167828	OK168865	–	–
78	0–20 cm	20200812-1-(6)_0–20 cm_B_R1_SC2	CSF21110	*C. aconidialis*	AA--	L.L. Liu, J.L. Han & L.S. Sun	OK167829	OK168866	–	–

78	0–20 cm	20200812-1-(6)_0–20 cm_B_R2_SC1	CSF21111	*C. kyotensis*	BAAA	L.L. Liu, J.L. Han & L.S. Sun	OK168006	OK169043	OK169170	OK169254
79	0–20 cm	20200812-1-(7)_0–20 cm_B_R1_SC1	CSF21112	*C. hongkongensis*	AA--	L.L. Liu, J.L. Han & L.S. Sun	OK167453	OK168490	–	–
79	0–20 cm	20200812-1-(7)_0–20 cm_B_R1_SC2	CSF21113	*C. hongkongensis*	AA--	L.L. Liu, J.L. Han & L.S. Sun	OK167454	OK168491	–	–
79	0–20 cm	20200812-1-(7)_0–20 cm_B_R2_SC1	CSF21114	*C. hongkongensis*	AA--	L.L. Liu, J.L. Han & L.S. Sun	OK167455	OK168492	–	–
79	0–20 cm	20200812-1-(7)_0–20 cm_B_R2_SC2	CSF21115	*C. hongkongensis*	AA--	L.L. Liu, J.L. Han & L.S. Sun	OK167456	OK168493	–	–
79	20–40 cm	20200812-1-(7)_0–40 cm_B_R2_SC2	CSF21117	*C. hongkongensis*	AA--	L.L. Liu, J.L. Han & L.S. Sun	OK167457	OK168494	–	–
79	20–40 cm	20200812-1-(7)_0–40 cm_B_R2_SC3	CSF21118	*C. hongkongensis*	AA--	L.L. Liu, J.L. Han & L.S. Sun	OK167458	OK168495	–	–
79	20–40 cm	20200812-1-(7)_0–40 cm_B_R2_SC4	CSF21119	*C. hongkongensis*	AA--	L.L. Liu, J.L. Han & L.S. Sun	OK167459	OK168496	–	–
80	0–20 cm	20200812-1-(8)_0–20 cm_A_R1_SC1	CSF21120	*C. hongkongensis*	AA--	L.L. Liu, J.L. Han & L.S. Sun	OK167460	OK168497	–	–
80	0–20 cm	20200812-1-(8)_0–20 cm_A_R1_SC2	CSF21121	*C. hongkongensis*	AA--	L.L. Liu, J.L. Han & L.S. Sun	OK167461	OK168498	–	–
80	0–20 cm	20200812-1-(8)_0–20 cm_B_R1_SC1	CSF21122	*C. hongkongensis*	AA--	L.L. Liu, J.L. Han & L.S. Sun	OK167462	OK168499	–	–
80	0–20 cm	20200812-1-(8)_0–20 cm_B_R1_SC2	CSF21123	*C. hongkongensis*	AA--	L.L. Liu, J.L. Han & L.S. Sun	OK167463	OK168500	–	–
80	0–20 cm	20200812-1-(8)_0–20 cm_B_R2_SC1	CSF21124	*C. hongkongensis*	AA--	L.L. Liu, J.L. Han & L.S. Sun	OK167464	OK168501	–	–
80	0–20 cm	20200812-1-(8)_0–20 cm_B_R2_SC2	CSF21125	*C. hongkongensis*	AA--	L.L. Liu, J.L. Han & L.S. Sun	OK167465	OK168502	–	–
80	20–40 cm	20200812-1-(8)_0–40 cm_A_R2_SC1	CSF21126	*C. ilicicola*	AAAB	L.L. Liu, J.L. Han & L.S. Sun	OK168010	OK169047	OK169173	OK169257
80	20–40 cm	20200812-1-(8)_0–40 cm_A_R2_SC2	CSF21127	*C. ilicicola*	AA--	L.L. Liu, J.L. Han & L.S. Sun	OK168011	OK169048	–	–
80	20–40 cm	20200812-1-(8)_0–40 cm_A_R2_SC3	CSF21128	*C. ilicicola*	AA--	L.L. Liu, J.L. Han & L.S. Sun	OK168012	OK169049	–	–

80	20–40 cm	20200812-1-(8)_0–40 cm_A_R2_SC4	CSF21129	*C. ilicicola*	AA--	L.L. Liu, J.L. Han & L.S. Sun	OK168013	OK169050	–	–
81	0–20 cm	20200813-1-(1)_0–20 cm_A_R1_SC1	CSF21130	*C. hongkongensis*	DA--	L.L. Liu, J.L. Han & L.S. Sun	OK167694	OK168731	–	–
81	0–20 cm	20200813-1-(1)_0–20 cm_A_R1_SC2	CSF21131	*C. hongkongensis*	AA--	L.L. Liu, J.L. Han & L.S. Sun	OK167466	OK168503	–	–
81	0–20 cm	20200813-1-(1)_0–20 cm_A_R2_SC1	CSF21132	*C. hongkongensis*	AA--	L.L. Liu, J.L. Han & L.S. Sun	OK167467	OK168504	–	–
81	0–20 cm	20200813-1-(1)_0–20 cm_A_R2_SC2	CSF21133	*C. hongkongensis*	AA--	L.L. Liu, J.L. Han & L.S. Sun	OK167468	OK168505	–	–
81	0–20 cm	20200813-1-(1)_0–20 cm_B_R1_SC1	CSF21134	*C. hongkongensis*	AA--	L.L. Liu, J.L. Han & L.S. Sun	OK167469	OK168506	–	–
81	0–20 cm	20200813-1-(1)_0–20 cm_B_R1_SC2	CSF21135	*C. hongkongensis*	AA--	L.L. Liu, J.L. Han & L.S. Sun	OK167470	OK168507	–	–
81	0–20 cm	20200813-1-(1)_0–20 cm_B_R2_SC1	CSF21136	*C. aconidialis*	AA--	L.L. Liu, J.L. Han & L.S. Sun	OK167830	OK168867	–	–
81	20–40 cm	20200813-1-(1)_0–40 cm_A_R2_SC1	CSF21138	*C. kyotensis*	AA--	L.L. Liu, J.L. Han & L.S. Sun	OK167974	OK169011	–	–
81	20–40 cm	20200813-1-(1)_0–40 cm_A_R2_SC2	CSF21139	*C. kyotensis*	AA--	L.L. Liu, J.L. Han & L.S. Sun	OK167975	OK169012	–	–
81	20–40 cm	20200813-1-(1)_0–40 cm_A_R2_SC3	CSF21140	*C. kyotensis*	AA--	L.L. Liu, J.L. Han & L.S. Sun	OK167976	OK169013	–	–
81	20–40 cm	20200813-1-(1)_0–40 cm_A_R2_SC4	CSF21141	*C. kyotensis*	AA--	L.L. Liu, J.L. Han & L.S. Sun	OK167977	OK169014	–	–
81	20–40 cm	20200813-1-(1)_0–40 cm_B_R1_SC1	CSF21142	*C. hongkongensis*	AA--	L.L. Liu, J.L. Han & L.S. Sun	OK167471	OK168508	–	–
81	20–40 cm	20200813-1-(1)_0–40 cm_B_R1_SC2	CSF21143	*C. hongkongensis*	AA--	L.L. Liu, J.L. Han & L.S. Sun	OK167472	OK168509	–	–
81	20–40 cm	20200813-1-(1)_0–40 cm_B_R1_SC3	CSF21144	*C. hongkongensis*	DA--	L.L. Liu, J.L. Han & L.S. Sun	OK167695	OK168732	–	–
81	20–40 cm	20200813-1-(1)_0–40 cm_B_R1_SC4	CSF21145	*C. hongkongensis*	AA--	L.L. Liu, J.L. Han & L.S. Sun	OK167473	OK168510	–	–
81	20–40 cm	20200813-1-(1)_0–40 cm_B_R2_SC1	CSF21146	*C. hongkongensis*	AA--	L.L. Liu, J.L. Han & L.S. Sun	OK167474	OK168511	–	–

81	20–40 cm	20200813-1-(1)_0–40 cm_B_R2_SC2	CSF21147	*C. hongkongensis*	AA--	L.L. Liu, J.L. Han & L.S. Sun	OK167475	OK168512	–	–
81	20–40 cm	20200813-1-(1)_0–40 cm_B_R2_SC3	CSF21148	*C. hongkongensis*	AA--	L.L. Liu, J.L. Han & L.S. Sun	OK167476	OK168513	–	–
81	20–40 cm	20200813-1-(1)_0–40 cm_B_R2_SC4	CSF21149	*C. hongkongensis*	AA--	L.L. Liu, J.L. Han & L.S. Sun	OK167477	OK168514	–	–
82	0–20 cm	20200813-1-(2)_0–20 cm_A_R1_SC1	CSF21150	*C. hongkongensis*	AA--	L.L. Liu, J.L. Han & L.S. Sun	OK167478	OK168515	–	–
82	0–20 cm	20200813-1-(2)_0–20 cm_A_R1_SC2	CSF21151	*C. hongkongensis*	AA--	L.L. Liu, J.L. Han & L.S. Sun	OK167479	OK168516	–	–
82	0–20 cm	20200813-1-(2)_0–20 cm_A_R2_SC2	CSF21152	*C. hongkongensis*	AA--	L.L. Liu, J.L. Han & L.S. Sun	OK167480	OK168517	–	–
82	0–20 cm	20200813-1-(2)_0–20 cm_B_R1_SC1	CSF21153	*C. hongkongensis*	AA--	L.L. Liu, J.L. Han & L.S. Sun	OK167481	OK168518	–	–
82	0–20 cm	20200813-1-(2)_0–20 cm_B_R1_SC2	CSF21154	*C. hongkongensis*	AA--	L.L. Liu, J.L. Han & L.S. Sun	OK167482	OK168519	–	–
82	0–20 cm	20200813-1-(2)_0–20 cm_B_R2_SC1	CSF21155	*C. hongkongensis*	AHAA	L.L. Liu, J.L. Han & L.S. Sun	OK167647	OK168684	OK169134	OK169218
82	0–20 cm	20200813-1-(2)_0–20 cm_B_R2_SC2	CSF21156	*C. hongkongensis*	AHAA	L.L. Liu, J.L. Han & L.S. Sun	OK167648	OK168685	OK169135	OK169219
83	0–20 cm	20200813-1-(3)_0–20 cm_B_R1_SC1	CSF21157	*C. hongkongensis*	AA--	L.L. Liu, J.L. Han & L.S. Sun	OK167483	OK168520	–	–
83	0–20 cm	20200813-1-(3)_0–20 cm_B_R1_SC2	CSF21158	*C. kyotensis*	AA--	L.L. Liu, J.L. Han & L.S. Sun	OK167978	OK169015	–	–
84	0–20 cm	20200813-1-(4)_0–20 cm_A_R1_SC1	CSF21159	*C. hongkongensis*	AA--	L.L. Liu, J.L. Han & L.S. Sun	OK167484	OK168521	–	–
84	0–20 cm	20200813-1-(4)_0–20 cm_A_R1_SC2	CSF21160	*C. hongkongensis*	AA--	L.L. Liu, J.L. Han & L.S. Sun	OK167485	OK168522	–	–
84	0–20 cm	20200813-1-(4)_0–20 cm_A_R2_SC1	CSF21161	*C. hongkongensis*	AA--	L.L. Liu, J.L. Han & L.S. Sun	OK167486	OK168523	–	–
84	0–20 cm	20200813-1-(4)_0–20 cm_A_R2_SC2	CSF21162	*C. hongkongensis*	AA--	L.L. Liu, J.L. Han & L.S. Sun	OK167487	OK168524	–	–
84	0–20 cm	20200813-1-(4)_0–20 cm_B_R1_SC1	CSF21163	*C. hongkongensis*	AA--	L.L. Liu, J.L. Han & L.S. Sun	OK167488	OK168525	–	–

84	0–20 cm	20200813-1-(4)_0–20 cm_B_R1_SC2	CSF21164	*C. hongkongensis*	AA--	L.L. Liu, J.L. Han & L.S. Sun	OK167489	OK168526	–	–
84	0–20 cm	20200813-1-(4)_0–20 cm_B_R2_SC1	CSF21165	*C. hongkongensis*	AA--	L.L. Liu, J.L. Han & L.S. Sun	OK167490	OK168527	–	–
84	0–20 cm	20200813-1-(4)_0–20 cm_B_R2_SC2	CSF21166	*C. hongkongensis*	AA--	L.L. Liu, J.L. Han & L.S. Sun	OK167491	OK168528	–	–
84	20–40 cm	20200813-1-(4)_0–40 cm_B_R2_SC1	CSF21167	*C. hongkongensis*	AF--	L.L. Liu, J.L. Han & L.S. Sun	OK167625	OK168662	–	–
84	20–40 cm	20200813-1-(4)_0–40 cm_B_R2_SC2	CSF21168	*C. hongkongensis*	AF--	L.L. Liu, J.L. Han & L.S. Sun	OK167626	OK168663	–	–
84	20–40 cm	20200813-1-(4)_0–40 cm_B_R2_SC3	CSF21169	*C. hongkongensis*	AF--	L.L. Liu, J.L. Han & L.S. Sun	OK167627	OK168664	–	–
84	20–40 cm	20200813-1-(4)_0–40 cm_B_R2_SC4	CSF21170	*C. hongkongensis*	AF--	L.L. Liu, J.L. Han & L.S. Sun	OK167628	OK168665	–	–
85	0–20 cm	20200814-1-(1)_0–20 cm_A_R1_SC1	CSF21171	*C. hongkongensis*	AA--	L.L. Liu, J.L. Han & L.S. Sun	OK167492	OK168529	–	–
85	0–20 cm	20200814-1-(1)_0–20 cm_A_R1_SC2	CSF21172	*C. hongkongensis*	AA--	L.L. Liu, J.L. Han & L.S. Sun	OK167493	OK168530	–	–
85	0–20 cm	20200814-1-(1)_0–20 cm_A_R2_SC1	CSF21173	*C. hongkongensis*	AA--	L.L. Liu, J.L. Han & L.S. Sun	OK167494	OK168531	–	–
85	0–20 cm	20200814-1-(1)_0–20 cm_A_R2_SC2	CSF21174	*C. hongkongensis*	AA--	L.L. Liu, J.L. Han & L.S. Sun	OK167495	OK168532	–	–
85	0–20 cm	20200814-1-(1)_0–20 cm_A_R2_SC3	CSF21175	*C. hongkongensis*	AA--	L.L. Liu, J.L. Han & L.S. Sun	OK167496	OK168533	–	–
85	0–20 cm	20200814-1-(1)_0–20 cm_A_R2_SC4	CSF21176	*C. hongkongensis*	AA--	L.L. Liu, J.L. Han & L.S. Sun	OK167497	OK168534	–	–
85	0–20 cm	20200814-1-(1)_0–20 cm_B_R1_SC1	CSF21177	*C. hongkongensis*	AA--	L.L. Liu, J.L. Han & L.S. Sun	OK167498	OK168535	–	–
85	0–20 cm	20200814-1-(1)_0–20 cm_B_R1_SC2	CSF21178	*C. hongkongensis*	AA--	L.L. Liu, J.L. Han & L.S. Sun	OK167499	OK168536	–	–
85	0–20 cm	20200814-1-(1)_0–20 cm_B_R2_SC1	CSF21179	*C. hongkongensis*	AA--	L.L. Liu, J.L. Han & L.S. Sun	OK167500	OK168537	–	–
85	0–20 cm	20200814-1-(1)_0–20 cm_B_R2_SC2	CSF21180	*C. hongkongensis*	AA--	L.L. Liu, J.L. Han & L.S. Sun	OK167501	OK168538	–	–

86	0–20 cm	20200814-1-(2)_0–20 cm_A_R2_SC1	CSF21181	*C. aconidialis*	AC--	L.L. Liu, J.L. Han & L.S. Sun	OK167939	OK168976	–	–
86	0–20 cm	20200814-1-(2)_0–20 cm_A_R2_SC2	CSF21182	*C. hongkongensis*	AA--	L.L. Liu, J.L. Han & L.S. Sun	OK167502	OK168539	–	–
86	0–20 cm	20200814-1-(2)_0–20 cm_B_R1_SC1	CSF21183	*C. hongkongensis*	BAAA	L.L. Liu, J.L. Han & L.S. Sun	OK167657	OK168694	OK169138	OK169222
86	0–20 cm	20200814-1-(2)_0–20 cm_B_R1_SC2	CSF21184	*C. hongkongensis*	BA--	L.L. Liu, J.L. Han & L.S. Sun	OK167658	OK168695	–	–
86	0–20 cm	20200814-1-(2)_0–20 cm_B_R2_SC1	CSF21185	*C. aconidialis*	AA--	L.L. Liu, J.L. Han & L.S. Sun	OK167831	OK168868	–	–
86	0–20 cm	20200814-1-(2)_0–20 cm_B_R2_SC2	CSF21186	*C. aconidialis*	AA--	L.L. Liu, J.L. Han & L.S. Sun	OK167832	OK168869	–	–
86	20–40 cm	20200814-1-(2)_0–40 cm_A_R1_SC1	CSF21187	*C. hongkongensis*	AA--	L.L. Liu, J.L. Han & L.S. Sun	OK167503	OK168540	–	–
86	20–40 cm	20200814-1-(2)_0–40 cm_A_R1_SC2	CSF21188	*C. hongkongensis*	AA--	L.L. Liu, J.L. Han & L.S. Sun	OK167504	OK168541	–	–
86	20–40 cm	20200814-1-(2)_0–40 cm_A_R1_SC3	CSF21189	*C. hongkongensis*	AA--	L.L. Liu, J.L. Han & L.S. Sun	OK167505	OK168542	–	–
86	20–40 cm	20200814-1-(2)_0–40 cm_A_R1_SC4	CSF21190	*C. hongkongensis*	AA--	L.L. Liu, J.L. Han & L.S. Sun	OK167506	OK168543	–	–
86	40–60 cm	20200814-1-(2)_0–60 cm_B_R2_SC1	CSF21191	*C. kyotensis*	ABAA	L.L. Liu, J.L. Han & L.S. Sun	OK167998	OK169035	OK169167	OK169251
86	40–60 cm	20200814-1-(2)_0–60 cm_B_R2_SC2	CSF21192	*C. kyotensis*	AB--	L.L. Liu, J.L. Han & L.S. Sun	OK167999	OK169036	–	–
86	40–60 cm	20200814-1-(2)_0–60 cm_B_R2_SC3	CSF21193	*C. kyotensis*	AB--	L.L. Liu, J.L. Han & L.S. Sun	OK168000	OK169037	–	–
86	40–60 cm	20200814-1-(2)_0–60 cm_B_R2_SC4	CSF21194	*C. kyotensis*	AB--	L.L. Liu, J.L. Han & L.S. Sun	OK168001	OK169038	–	–
87	0–20 cm	20200814-1-(3)_0–20 cm_A_R1_SC1	CSF21195	*C. hongkongensis*	AA--	L.L. Liu, J.L. Han & L.S. Sun	OK167507	OK168544	–	–
87	0–20 cm	20200814-1-(3)_0–20 cm_A_R1_SC2	CSF21196	*C. hongkongensis*	AA--	L.L. Liu, J.L. Han & L.S. Sun	OK167508	OK168545	–	–
87	0–20 cm	20200814-1-(3)_0–20 cm_A_R2_SC1	CSF21197	*C. hongkongensis*	AA--	L.L. Liu, J.L. Han & L.S. Sun	OK167509	OK168546	–	–

87	0–20 cm	20200814-1-(3)_0–20 cm_A_R2_SC2	CSF21198	*C. ilicicola*	BBBA	L.L. Liu, J.L. Han & L.S. Sun	OK168047	OK169084	OK169182	OK169266
87	0–20 cm	20200814-1-(3)_0–20 cm_B_R1_SC1	CSF21199	*C. hongkongensis*	AA--	L.L. Liu, J.L. Han & L.S. Sun	OK167510	OK168547	–	–
87	0–20 cm	20200814-1-(3)_0–20 cm_B_R1_SC2	CSF21200	*C. hongkongensis*	AA--	L.L. Liu, J.L. Han & L.S. Sun	OK167511	OK168548	–	–
87	0–20 cm	20200814-1-(3)_0–20 cm_B_R2_SC1	CSF21201	*C. aconidialis*	AA--	L.L. Liu, J.L. Han & L.S. Sun	OK167833	OK168870	–	–
87	0–20 cm	20200814-1-(3)_0–20 cm_B_R2_SC2	CSF21202	*C. aconidialis*	AA--	L.L. Liu, J.L. Han & L.S. Sun	OK167834	OK168871	–	–
88	0–20 cm	20200815-1-(1)_0–20 cm_A_R1_SC1	CSF21203	*C. hongkongensis*	AA--	L.L. Liu, J.L. Han & L.S. Sun	OK167512	OK168549	–	–
88	0–20 cm	20200815-1-(1)_0–20 cm_A_R1_SC2	CSF21204	*C. hongkongensis*	AA--	L.L. Liu, J.L. Han & L.S. Sun	OK167513	OK168550	–	–
88	0–20 cm	20200815-1-(1)_0–20 cm_A_R2_SC1	CSF21205	*C. hongkongensis*	AA--	L.L. Liu, J.L. Han & L.S. Sun	OK167514	OK168551	–	–
88	0–20 cm	20200815-1-(1)_0–20 cm_A_R2_SC2	CSF21206	*C. hongkongensis*	AA--	L.L. Liu, J.L. Han & L.S. Sun	OK167515	OK168552	–	–
88	0–20 cm	20200815-1-(1)_0–20 cm_B_R1_SC1	CSF21207	*C. hongkongensis*	AA--	L.L. Liu, J.L. Han & L.S. Sun	OK167516	OK168553	–	–
88	0–20 cm	20200815-1-(1)_0–20 cm_B_R1_SC2	CSF21208	*C. hongkongensis*	AA--	L.L. Liu, J.L. Han & L.S. Sun	OK167517	OK168554	–	–
88	0–20 cm	20200815-1-(1)_0–20 cm_B_R2_SC1	CSF21209	*C. hongkongensis*	AA--	L.L. Liu, J.L. Han & L.S. Sun	OK167518	OK168555	–	–
88	0–20 cm	20200815-1-(1)_0–20 cm_B_R2_SC2	CSF21210	*C. ilicicola*	BB--	L.L. Liu, J.L. Han & L.S. Sun	OK168048	OK169085	–	–
88	20–40 cm	20200815-1-(1)_0–40 cm_A_R1_SC1	CSF21211	*C. hongkongensis*	AA--	L.L. Liu, J.L. Han & L.S. Sun	OK167519	OK168556	–	–
88	20–40 cm	20200815-1-(1)_0–40 cm_A_R1_SC2	CSF21212	*C. hongkongensis*	AA--	L.L. Liu, J.L. Han & L.S. Sun	OK167520	OK168557	–	–
88	20–40 cm	20200815-1-(1)_0–40 cm_A_R1_SC3	CSF21213	*C. hongkongensis*	AA--	L.L. Liu, J.L. Han & L.S. Sun	OK167521	OK168558	–	–
88	20–40 cm	20200815-1-(1)_0–40 cm_A_R1_SC4	CSF21214	*C. hongkongensis*	AA--	L.L. Liu, J.L. Han & L.S. Sun	OK167522	OK168559	–	–

88	60–80 cm	20200815-1-(1)_0–80 cm_B_R1_SC1	CSF21215	*C. ilicicola*	BB--	L.L. Liu, J.L. Han & L.S. Sun	OK168049	OK169086	–	–
88	60–80 cm	20200815-1-(1)_0–80 cm_B_R1_SC2	CSF21216	*C. ilicicola*	BB--	L.L. Liu, J.L. Han & L.S. Sun	OK168050	OK169087	–	–
88	60–80 cm	20200815-1-(1)_0–80 cm_B_R1_SC3	CSF21217	*C. ilicicola*	BB--	L.L. Liu, J.L. Han & L.S. Sun	OK168051	OK169088	–	–
88	60–80 cm	20200815-1-(1)_0–80 cm_B_R1_SC4	CSF21218	*C. ilicicola*	BB--	L.L. Liu, J.L. Han & L.S. Sun	OK168052	OK169089	–	–
89	0–20 cm	20200815-1-(2)_0–20 cm_A_R2_SC1	CSF21219	*C. ilicicola*	AAAB	L.L. Liu, J.L. Han & L.S. Sun	OK168014	OK169051	OK169174	OK169258
89	0–20 cm	20200815-1-(2)_0–20 cm_A_R2_SC2	CSF21220	*C. ilicicola*	AA--	L.L. Liu, J.L. Han & L.S. Sun	OK168015	OK169052	–	–
89	0–20 cm	20200815-1-(2)_0–20 cm_B_R1_SC1	CSF21221	*C. hongkongensis*	AA--	L.L. Liu, J.L. Han & L.S. Sun	OK167523	OK168560	–	–
89	0–20 cm	20200815-1-(2)_0–20 cm_B_R1_SC2	CSF21222	*C. hongkongensis*	AA--	L.L. Liu, J.L. Han & L.S. Sun	OK167524	OK168561	–	–
89	80–100 cm	20200815-1-(2)_0–100 cm_B_R2_SC1	CSF21223	*C. aconidialis*	AA--	L.L. Liu, J.L. Han & L.S. Sun	OK167835	OK168872	–	–
89	80–100 cm	20200815-1-(2)_0–100 cm_B_R2_SC2	CSF21224	*C. aconidialis*	AA--	L.L. Liu, J.L. Han & L.S. Sun	OK167836	OK168873	–	–
89	80–100 cm	20200815-1-(2)_0–100 cm_B_R2_SC3	CSF21225	*C. aconidialis*	AA--	L.L. Liu, J.L. Han & L.S. Sun	OK167837	OK168874	–	–
89	80–100 cm	20200815-1-(2)_0–100 cm_B_R2_SC4	CSF21226	*C. aconidialis*	AA--	L.L. Liu, J.L. Han & L.S. Sun	OK167838	OK168875	–	–
90	0–20 cm	20200815-1-(3)_0–20 cm_A_R1_SC1	CSF21227	*C. hongkongensis*	AA--	L.L. Liu, J.L. Han & L.S. Sun	OK167525	OK168562	–	–
90	0–20 cm	20200815-1-(3)_0–20 cm_A_R1_SC2	CSF21228	*C. hongkongensis*	AA--	L.L. Liu, J.L. Han & L.S. Sun	OK167526	OK168563	–	–
90	0–20 cm	20200815-1-(3)_0–20 cm_A_R2_SC1	CSF21229	*C. hongkongensis*	AA--	L.L. Liu, J.L. Han & L.S. Sun	OK167527	OK168564	–	–
90	0–20 cm	20200815-1-(3)_0–20 cm_A_R2_SC2	CSF21230	*C. hongkongensis*	AA--	L.L. Liu, J.L. Han & L.S. Sun	OK167528	OK168565	–	–
90	0–20 cm	20200815-1-(3)_0–20 cm_B_R1_SC1	CSF21231	*C. hongkongensis*	AA--	L.L. Liu, J.L. Han & L.S. Sun	OK167529	OK168566	–	–

90	0–20 cm	20200815-1-(3)_0–20 cm_B_R2_SC2	CSF21233	*C. hongkongensis*	AFAA	L.L. Liu, J.L. Han & L.S. Sun	OK167629	OK168666	OK169127	OK169211
90	20–40 cm	20200815-1-(3)_0–40 cm_A_R1_SC1	CSF21234	*C. hongkongensis*	AA--	L.L. Liu, J.L. Han & L.S. Sun	OK167530	OK168567	–	–
90	20–40 cm	20200815-1-(3)_0–40 cm_A_R1_SC2	CSF21235	*C. hongkongensis*	AA--	L.L. Liu, J.L. Han & L.S. Sun	OK167531	OK168568	–	–
90	20–40 cm	20200815-1-(3)_0–40 cm_A_R1_SC3	CSF21236	*C. hongkongensis*	AA--	L.L. Liu, J.L. Han & L.S. Sun	OK167532	OK168569	–	–
90	20–40 cm	20200815-1-(3)_0–40 cm_A_R1_SC4	CSF21237	*C. hongkongensis*	AA--	L.L. Liu, J.L. Han & L.S. Sun	OK167533	OK168570	–	–
90	20–40 cm	20200815-1-(3)_0–40 cm_A_R2_SC1	CSF21238	*C. hongkongensis*	AA--	L.L. Liu, J.L. Han & L.S. Sun	OK167534	OK168571	–	–
90	20–40 cm	20200815-1-(3)_0–40 cm_A_R2_SC2	CSF21239	*C. hongkongensis*	AA--	L.L. Liu, J.L. Han & L.S. Sun	OK167535	OK168572	–	–
90	20–40 cm	20200815-1-(3)_0–40 cm_A_R2_SC3	CSF21240	*C. hongkongensis*	AA--	L.L. Liu, J.L. Han & L.S. Sun	OK167536	OK168573	–	–
90	20–40 cm	20200815-1-(3)_0–40 cm_A_R2_SC4	CSF21241	*C. aconidialis*	AC--	L.L. Liu, J.L. Han & L.S. Sun	OK167940	OK168977	–	–
90	20–40 cm	20200815-1-(3)_0–40 cm_B_R2_SC1	CSF21242	*C. hongkongensis*	AA--	L.L. Liu, J.L. Han & L.S. Sun	OK167537	OK168574	–	–
90	20–40 cm	20200815-1-(3)_0–40 cm_B_R2_SC2	CSF21243	*C. hongkongensis*	AA--	L.L. Liu, J.L. Han & L.S. Sun	OK167538	OK168575	–	–
90	20–40 cm	20200815-1-(3)_0–40 cm_B_R2_SC4	CSF21245	*C. hongkongensis*	AA--	L.L. Liu, J.L. Han & L.S. Sun	OK167539	OK168576	–	–
91	0–20 cm	20200815-1-(4)_0–20 cm_A_R2_SC1	CSF21246	*C. aconidialis*	AA--	L.L. Liu, J.L. Han & L.S. Sun	OK167839	OK168876	–	–
91	0–20 cm	20200815-1-(4)_0–20 cm_A_R2_SC2	CSF21247	*C. aconidialis*	AA--	L.L. Liu, J.L. Han & L.S. Sun	OK167840	OK168877	–	–
91	0–20 cm	20200815-1-(4)_0–20 cm_B_R1_SC1	CSF21248	*C. hongkongensis*	AA--	L.L. Liu, J.L. Han & L.S. Sun	OK167540	OK168577	–	–
91	0–20 cm	20200815-1-(4)_0–20 cm_B_R1_SC2	CSF21249	*C. hongkongensis*	AA--	L.L. Liu, J.L. Han & L.S. Sun	OK167541	OK168578	–	–
91	0–20 cm	20200815-1-(4)_0–20 cm_B_R2_SC1	CSF21250	*C. hongkongensis*	AA--	L.L. Liu, J.L. Han & L.S. Sun	OK167542	OK168579	–	–

92	0–20 cm	20200815-1-(5)_0–20 cm_A_R1_SC1	CSF21252	*C. hongkongensis*	DA--	L.L. Liu, J.L. Han & L.S. Sun	OK167696	OK168733	–	–
92	0–20 cm	20200815-1-(5)_0–20 cm_A_R1_SC2	CSF21253	*C. hongkongensis*	DA--	L.L. Liu, J.L. Han & L.S. Sun	OK167697	OK168734	–	–
92	0–20 cm	20200815-1-(5)_0–20 cm_A_R2_SC1	CSF21254	*C. aconidialis*	AA--	L.L. Liu, J.L. Han & L.S. Sun	OK167841	OK168878	–	–
92	0–20 cm	20200815-1-(5)_0–20 cm_A_R2_SC2	CSF21255	*C. aconidialis*	AA--	L.L. Liu, J.L. Han & L.S. Sun	OK167842	OK168879	–	–
92	0–20 cm	20200815-1-(5)_0–20 cm_B_R1_SC1	CSF21256	*C. aconidialis*	AC--	L.L. Liu, J.L. Han & L.S. Sun	OK167941	OK168978	–	–
92	0–20 cm	20200815-1-(5)_0–20 cm_B_R1_SC2	CSF21257	*C. hongkongensis*	DA--	L.L. Liu, J.L. Han & L.S. Sun	OK167698	OK168735	–	–
92	0–20 cm	20200815-1-(5)_0–20 cm_B_R2_SC1	CSF21258	*C. hongkongensis*	AA--	L.L. Liu, J.L. Han & L.S. Sun	OK167543	OK168580	–	–
92	0–20 cm	20200815-1-(5)_0–20 cm_B_R2_SC2	CSF21259	*C. hongkongensis*	AA--	L.L. Liu, J.L. Han & L.S. Sun	OK167544	OK168581	–	–
93	0–20 cm	20200816-1-(1)_0–20 cm_B_R1_SC1	CSF21260	*C. aconidialis*	AA--	L.L. Liu, J.L. Han & L.S. Sun	OK167843	OK168880	–	–
93	0–20 cm	20200816-1-(1)_0–20 cm_B_R1_SC2	CSF21261	*C. aconidialis*	AA--	L.L. Liu, J.L. Han & L.S. Sun	OK167844	OK168881	–	–
93	20–40 cm	20200816-1-(1)_0–40 cm_B_R1_SC1	CSF21262	*C. aconidialis*	ABBA	L.L. Liu, J.L. Han & L.S. Sun	OK167857	OK168894	OK169153	OK169237
93	20–40 cm	20200816-1-(1)_0–40 cm_B_R1_SC2	CSF21263	*C. aconidialis*	AB--	L.L. Liu, J.L. Han & L.S. Sun	OK167858	OK168895	–	–
93	20–40 cm	20200816-1-(1)_0–40 cm_B_R1_SC3	CSF21264	*C. aconidialis*	AB--	L.L. Liu, J.L. Han & L.S. Sun	OK167859	OK168896	–	–
93	20–40 cm	20200816-1-(1)_0–40 cm_B_R1_SC4	CSF21265	*C. aconidialis*	AB--	L.L. Liu, J.L. Han & L.S. Sun	OK167860	OK168897	–	–
93	20–40 cm	20200816-1-(1)_0–40 cm_B_R2_SC2	CSF21266	*C. aconidialis*	ABBA	L.L. Liu, J.L. Han & L.S. Sun	OK167861	OK168898	OK169154	OK169238
93	20–40 cm	20200816-1-(1)_0–40 cm_B_R2_SC3	CSF21267	*C. aconidialis*	AB--	L.L. Liu, J.L. Han & L.S. Sun	OK167862	OK168899	–	–
93	20–40 cm	20200816-1-(1)_0–40 cm_B_R2_SC4	CSF21268	*C. aconidialis*	AB--	L.L. Liu, J.L. Han & L.S. Sun	OK167863	OK168900	–	–

93	60–80 cm	20200816-1-(1)_0–80 cm_A_R1_SC1	CSF21269	*C. aconidialis*	AA--	L.L. Liu, J.L. Han & L.S. Sun	OK167845	OK168882	–	–
93	60–80 cm	20200816-1-(1)_0–80 cm_A_R1_SC2	CSF21270	*C. aconidialis*	AA--	L.L. Liu, J.L. Han & L.S. Sun	OK167846	OK168883	–	–
93	60–80 cm	20200816-1-(1)_0–80 cm_A_R1_SC3	CSF21271	*C. aconidialis*	AA--	L.L. Liu, J.L. Han & L.S. Sun	OK167847	OK168884	–	–
93	60–80 cm	20200816-1-(1)_0–80 cm_A_R1_SC4	CSF21272	*C. aconidialis*	AA--	L.L. Liu, J.L. Han & L.S. Sun	OK167848	OK168885	–	–
94	0–20 cm	20200816-1-(2)_0–20 cm_A_R1_SC1	CSF21276	*C. hongkongensis*	AA--	L.L. Liu, J.L. Han & L.S. Sun	OK167545	OK168582	–	–
94	0–20 cm	20200816-1-(2)_0–20 cm_A_R1_SC2	CSF21277	*C. hongkongensis*	AA--	L.L. Liu, J.L. Han & L.S. Sun	OK167546	OK168583	–	–
94	0–20 cm	20200816-1-(2)_0–20 cm_A_R2_SC1	CSF21278	*C. hongkongensis*	AA--	L.L. Liu, J.L. Han & L.S. Sun	OK167547	OK168584	–	–
94	0–20 cm	20200816-1-(2)_0–20 cm_A_R2_SC2	CSF21279	*C. hongkongensis*	AA--	L.L. Liu, J.L. Han & L.S. Sun	OK167548	OK168585	–	–
94	0–20 cm	20200816-1-(2)_0–20 cm_B_R1_SC1	CSF21280	*C. hongkongensis*	AA--	L.L. Liu, J.L. Han & L.S. Sun	OK167549	OK168586	–	–
94	0–20 cm	20200816-1-(2)_0–20 cm_B_R1_SC2	CSF21281	*C. hongkongensis*	AA--	L.L. Liu, J.L. Han & L.S. Sun	OK167550	OK168587	–	–
94	0–20 cm	20200816-1-(2)_0–20 cm_B_R2_SC1	CSF21282	*C. hongkongensis*	AA--	L.L. Liu, J.L. Han & L.S. Sun	OK167551	OK168588	–	–
94	0–20 cm	20200816-1-(2)_0–20 cm_B_R2_SC2	CSF21283	*C. hongkongensis*	AA--	L.L. Liu, J.L. Han & L.S. Sun	OK167552	OK168589	–	–
94	20–40 cm	20200816-1-(2)_0–40 cm_A_R1_SC1	CSF21284	*C. hongkongensis*	AA--	L.L. Liu, J.L. Han & L.S. Sun	OK167553	OK168590	–	–
94	20–40 cm	20200816-1-(2)_0–40 cm_A_R1_SC2	CSF21285	*C. hongkongensis*	AA--	L.L. Liu, J.L. Han & L.S. Sun	OK167554	OK168591	–	–
94	20–40 cm	20200816-1-(2)_0–40 cm_A_R1_SC3	CSF21286	*C. hongkongensis*	AA--	L.L. Liu, J.L. Han & L.S. Sun	OK167555	OK168592	–	–
94	20–40 cm	20200816-1-(2)_0–40 cm_A_R1_SC4	CSF21287	*C. hongkongensis*	AA--	L.L. Liu, J.L. Han & L.S. Sun	OK167556	OK168593	–	–
94	20–40 cm	20200816-1-(2)_0–40 cm_A_R2_SC1	CSF21288	*C. hongkongensis*	AA--	L.L. Liu, J.L. Han & L.S. Sun	OK167557	OK168594	–	–

94	20–40 cm	20200816-1-(2)_0–40 cm_A_R2_SC2	CSF21289	*C. hongkongensis*	AA--	L.L. Liu, J.L. Han & L.S. Sun	OK167558	OK168595	–	–
94	20–40 cm	20200816-1-(2)_0–40 cm_A_R2_SC3	CSF21290	*C. hongkongensis*	AA--	L.L. Liu, J.L. Han & L.S. Sun	OK167559	OK168596	–	–
94	20–40 cm	20200816-1-(2)_0–40 cm_A_R2_SC4	CSF21291	*C. hongkongensis*	AA--	L.L. Liu, J.L. Han & L.S. Sun	OK167560	OK168597	–	–
95	0–20 cm	20200816-1-(3)_0–20 cm_A_R1_SC1	CSF21292	*C. ilicicola*	BBBA	L.L. Liu, J.L. Han & L.S. Sun	OK168053	OK169090	OK169183	OK169267
95	0–20 cm	20200816-1-(3)_0–20 cm_A_R1_SC2	CSF21293	*C. ilicicola*	BB--	L.L. Liu, J.L. Han & L.S. Sun	OK168054	OK169091	–	–
95	0–20 cm	20200816-1-(3)_0–20 cm_A_R2_SC1	CSF21294	*C. aconidialis*	AC--	L.L. Liu, J.L. Han & L.S. Sun	OK167942	OK168979	–	–
95	0–20 cm	20200816-1-(3)_0–20 cm_A_R2_SC2	CSF21295	*C. aconidialis*	AC--	L.L. Liu, J.L. Han & L.S. Sun	OK167943	OK168980	–	–
95	0–20 cm	20200816-1-(3)_0–20 cm_B_R1_SC1	CSF21296	*C. aconidialis*	AA--	L.L. Liu, J.L. Han & L.S. Sun	OK167849	OK168886	–	–
95	0–20 cm	20200816-1-(3)_0–20 cm_B_R1_SC2	CSF21297	*C. hongkongensis*	AA--	L.L. Liu, J.L. Han & L.S. Sun	OK167561	OK168598	–	–
95	20–40 cm	20200816-1-(3)_0–40 cm_B_R1_SC1	CSF21298	*C. hongkongensis*	AA--	L.L. Liu, J.L. Han & L.S. Sun	OK167562	OK168599	–	–
95	20–40 cm	20200816-1-(3)_0–40 cm_B_R1_SC2	CSF21299	*C. hongkongensis*	AA--	L.L. Liu, J.L. Han & L.S. Sun	OK167563	OK168600	–	–
95	20–40 cm	20200816-1-(3)_0–40 cm_B_R1_SC3	CSF21300	*C. hongkongensis*	AA--	L.L. Liu, J.L. Han & L.S. Sun	OK167564	OK168601	–	–
95	20–40 cm	20200816-1-(3)_0–40 cm_B_R1_SC4	CSF21301	*C. hongkongensis*	AA--	L.L. Liu, J.L. Han & L.S. Sun	OK167565	OK168602	–	–
96	0–20 cm	20200816-1-(4)_0–20 cm_A_R1_SC1	CSF21302	*C. hongkongensis*	AA--	L.L. Liu, J.L. Han & L.S. Sun	OK167566	OK168603	–	–
96	0–20 cm	20200816-1-(4)_0–20 cm_A_R1_SC2	CSF21303	*C. hongkongensis*	AA--	L.L. Liu, J.L. Han & L.S. Sun	OK167567	OK168604	–	–
96	0–20 cm	20200816-1-(4)_0–20 cm_A_R2_SC1	CSF21304	*C. hongkongensis*	ADAA	L.L. Liu, J.L. Han & L.S. Sun	OK167607	OK168644	OK169121	OK169205
96	0–20 cm	20200816-1-(4)_0–20 cm_A_R2_SC2	CSF21305	*C. hongkongensis*	AA--	L.L. Liu, J.L. Han & L.S. Sun	OK167568	OK168605	–	–

96	0–20 cm	20200816-1-(4)_0–20 cm_B_R1_SC2	CSF21307	*C. hongkongensis*	AA--	L.L. Liu, J.L. Han & L.S. Sun	OK167569	OK168606	–	–
96	0–20 cm	20200816-1-(4)_0–20 cm_B_R2_SC1	CSF21308	*C. hongkongensis*	AA--	L.L. Liu, J.L. Han & L.S. Sun	OK167570	OK168607	–	–
96	0–20 cm	20200816-1-(4)_0–20 cm_B_R2_SC2	CSF21309	*C. hongkongensis*	BA--	L.L. Liu, J.L. Han & L.S. Sun	OK167659	OK168696	–	–
96	20–40 cm	20200816-1-(4)_0–40 cm_A_R1_SC1	CSF21310	*C. ilicicola*	AAAB	L.L. Liu, J.L. Han & L.S. Sun	OK168016	OK169053	OK169175	OK169259
96	20–40 cm	20200816-1-(4)_0–40 cm_A_R1_SC2	CSF21311	*C. ilicicola*	AA--	L.L. Liu, J.L. Han & L.S. Sun	OK168017	OK169054	–	–
96	20–40 cm	20200816-1-(4)_0–40 cm_A_R1_SC3	CSF21312	*C. ilicicola*	AA--	L.L. Liu, J.L. Han & L.S. Sun	OK168018	OK169055	–	–
96	20–40 cm	20200816-1-(4)_0–40 cm_A_R1_SC4	CSF21313	*C. ilicicola*	AA--	L.L. Liu, J.L. Han & L.S. Sun	OK168019	OK169056	–	–
96	20–40 cm	20200816-1-(4)_0–40 cm_A_R2_SC1	CSF21314	*C. hongkongensis*	AA--	L.L. Liu, J.L. Han & L.S. Sun	OK167571	OK168608	–	–
96	20–40 cm	20200816-1-(4)_0–40 cm_A_R2_SC2	CSF21315	*C. hongkongensis*	AA--	L.L. Liu, J.L. Han & L.S. Sun	OK167572	OK168609	–	–
96	20–40 cm	20200816-1-(4)_0–40 cm_A_R2_SC3	CSF21316	*C. hongkongensis*	AA--	L.L. Liu, J.L. Han & L.S. Sun	OK167573	OK168610	–	–
96	20–40 cm	20200816-1-(4)_0–40 cm_A_R2_SC4	CSF21317	*C. hongkongensis*	AA--	L.L. Liu, J.L. Han & L.S. Sun	OK167574	OK168611	–	–
96	20–40 cm	20200816-1-(4)_0–40 cm_B_R1_SC1	CSF21318	*C. ilicicola*	AA--	L.L. Liu, J.L. Han & L.S. Sun	OK168020	OK169057	–	–
96	20–40 cm	20200816-1-(4)_0–40 cm_B_R1_SC2	CSF21319	*C. ilicicola*	AA--	L.L. Liu, J.L. Han & L.S. Sun	OK168021	OK169058	–	–
96	20–40 cm	20200816-1-(4)_0–40 cm_B_R1_SC3	CSF21320	*C. ilicicola*	AA--	L.L. Liu, J.L. Han & L.S. Sun	OK168022	OK169059	–	–
96	20–40 cm	20200816-1-(4)_0–40 cm_B_R1_SC4	CSF21321	*C. ilicicola*	AA--	L.L. Liu, J.L. Han & L.S. Sun	OK168023	OK169060	–	–
96	20–40 cm	20200816-1-(4)_0–40 cm_B_R2_SC1	CSF21322	*C. aconidialis*	AA--	L.L. Liu, J.L. Han & L.S. Sun	OK167850	OK168887	–	–
96	20–40 cm	20200816-1-(4)_0–40 cm_B_R2_SC2	CSF21323	*C. ilicicola*	AA--	L.L. Liu, J.L. Han & L.S. Sun	OK168024	OK169061	–	–

96	20–40 cm	20200816-1-(4)_0–40 cm_B_R2_SC3	CSF21324	*C. ilicicola*	AA--	L.L. Liu, J.L. Han & L.S. Sun	OK168025	OK169062	–	–
96	20–40 cm	20200816-1-(4)_0–40 cm_B_R2_SC4	CSF21325	*C. aconidialis*	AA--	L.L. Liu, J.L. Han & L.S. Sun	OK167851	OK168888	–	–
96	40–60 cm	20200816-1-(4)_0–60 cm_B_R1_SC1	CSF21326	*C. ilicicola*	AA--	L.L. Liu, J.L. Han & L.S. Sun	OK168026	OK169063	–	–
96	40–60 cm	20200816-1-(4)_0–60 cm_B_R1_SC2	CSF21327	*C. ilicicola*	AA--	L.L. Liu, J.L. Han & L.S. Sun	OK168027	OK169064	–	–
96	40–60 cm	20200816-1-(4)_0–60 cm_B_R1_SC3	CSF21328	*C. ilicicola*	AA--	L.L. Liu, J.L. Han & L.S. Sun	OK168028	OK169065	–	–
96	40–60 cm	20200816-1-(4)_0–60 cm_B_R1_SC4	CSF21329	*C. ilicicola*	AA--	L.L. Liu, J.L. Han & L.S. Sun	OK168029	OK169066	–	–
96	40–60 cm	20200816-1-(4)_0–60 cm_B_R2_SC1	CSF21330	*C. ilicicola*	AA--	L.L. Liu, J.L. Han & L.S. Sun	OK168030	OK169067	–	–
96	40–60 cm	20200816-1-(4)_0–60 cm_B_R2_SC2	CSF21331	*C. ilicicola*	AA--	L.L. Liu, J.L. Han & L.S. Sun	OK168031	OK169068	–	–
96	40–60 cm	20200816-1-(4)_0–60 cm_B_R2_SC3	CSF21332	*C. ilicicola*	AA--	L.L. Liu, J.L. Han & L.S. Sun	OK168032	OK169069	–	–
96	40–60 cm	20200816-1-(4)_0–60 cm_B_R2_SC4	CSF21333	*C. ilicicola*	AA--	L.L. Liu, J.L. Han & L.S. Sun	OK168033	OK169070	–	–
97	0–20 cm	20200816-1-(5)_0–20 cm_A_R1_SC1	CSF21334	*C. aconidialis*	AA--	L.L. Liu, J.L. Han & L.S. Sun	OK167852	OK168889	–	–
97	0–20 cm	20200816-1-(5)_0–20 cm_A_R1_SC2	CSF21335	*C. kyotensis*	BAAA	L.L. Liu, J.L. Han & L.S. Sun	OK168007	OK169044	OK169171	OK169255
97	0–20 cm	20200816-1-(5)_0–20 cm_A_R2_SC1	CSF21336	*C. aconidialis*	AC--	L.L. Liu, J.L. Han & L.S. Sun	OK167944	OK168981	–	–
97	0–20 cm	20200816-1-(5)_0–20 cm_A_R2_SC2	CSF21337	*C. aconidialis*	AA--	L.L. Liu, J.L. Han & L.S. Sun	OK167853	OK168890	–	–
97	0–20 cm	20200816-1-(5)_0–20 cm_B_R1_SC1	CSF21338	*C. aconidialis*	AC--	L.L. Liu, J.L. Han & L.S. Sun	OK167945	OK168982	–	–
97	0–20 cm	20200816-1-(5)_0–20 cm_B_R1_SC2	CSF21339	*C. kyotensis*	AA--	L.L. Liu, J.L. Han & L.S. Sun	OK167979	OK169016	–	–
97	0–20 cm	20200816-1-(5)_0–20 cm_B_R2_SC1	CSF21340	*C. aconidialis*	AA--	L.L. Liu, J.L. Han & L.S. Sun	OK167854	OK168891	–	–

97	0–20 cm	20200816-1-(5)_0–20 cm_B_R2_SC2	CSF21341	*C. kyotensis*	AA--	L.L. Liu, J.L. Han & L.S. Sun	OK167980	OK169017	–	–
97	40–60 cm	20200816-1-(5)_0–60 cm_B_R2_SC1	CSF21342	*C. hongkongensis*	AA--	L.L. Liu, J.L. Han & L.S. Sun	OK167575	OK168612	–	–
97	40–60 cm	20200816-1-(5)_0–60 cm_B_R2_SC2	CSF21343	*C. hongkongensis*	AA--	L.L. Liu, J.L. Han & L.S. Sun	OK167576	OK168613	–	–
97	40–60 cm	20200816-1-(5)_0–60 cm_B_R2_SC3	CSF21344	*C. hongkongensis*	AA--	L.L. Liu, J.L. Han & L.S. Sun	OK167577	OK168614	–	–
97	40–60 cm	20200816-1-(5)_0–60 cm_B_R2_SC4	CSF21345	*C. hongkongensis*	AA--	L.L. Liu, J.L. Han & L.S. Sun	OK167578	OK168615	–	–
98	0–20 cm	20200816-1-(6)_0–20 cm_A_R1_SC1	CSF21346	*C. aconidialis*	ACAA	L.L. Liu, J.L. Han & L.S. Sun	OK167946	OK168983	OK169159	OK169243
98	0–20 cm	20200816-1-(6)_0–20 cm_A_R1_SC2	CSF21347	*C. aconidialis*	AC--	L.L. Liu, J.L. Han & L.S. Sun	OK167947	OK168984	–	–
98	0–20 cm	20200816-1-(6)_0–20 cm_A_R2_SC1	CSF21348	*C. aconidialis*	AAAA	L.L. Liu, J.L. Han & L.S. Sun	OK167855	OK168892	OK169151	OK169235
98	0–20 cm	20200816-1-(6)_0–20 cm_A_R2_SC2	CSF21349	*C. aconidialis*	ABBA	L.L. Liu, J.L. Han & L.S. Sun	OK167864	OK168901	OK169155	OK169239
98	0–20 cm	20200816-1-(6)_0–20 cm_B_R1_SC1	CSF21350	*C. kyotensis*	AAAA	L.L. Liu, J.L. Han & L.S. Sun	OK167981	OK169018	OK169163	OK169247
98	0–20 cm	20200816-1-(6)_0–20 cm_B_R1_SC2	CSF21351	*C. kyotensis*	AA--	L.L. Liu, J.L. Han & L.S. Sun	OK167982	OK169019	–	–
98	0–20 cm	20200816-1-(6)_0–20 cm_B_R2_SC1	CSF21352	*C. aconidialis*	AC--	L.L. Liu, J.L. Han & L.S. Sun	OK167948	OK168985	–	–
98	0–20 cm	20200816-1-(6)_0–20 cm_B_R2_SC2	CSF21353	*C. aconidialis*	AC--	L.L. Liu, J.L. Han & L.S. Sun	OK167949	OK168986	–	–
98	60–80 cm	20200816-1-(6)_0–80 cm_B_R2_SC1	CSF21354	*C. hongkongensis*	AA--	L.L. Liu, J.L. Han & L.S. Sun	OK167579	OK168616	–	–
98	60–80 cm	20200816-1-(6)_0–80 cm_B_R2_SC2	CSF21355	*C. hongkongensis*	AA--	L.L. Liu, J.L. Han & L.S. Sun	OK167580	OK168617	–	–
98	60–80 cm	20200816-1-(6)_0–80 cm_B_R2_SC3	CSF21356	*C. hongkongensis*	AA--	L.L. Liu, J.L. Han & L.S. Sun	OK167581	OK168618	–	–
98	60–80 cm	20200816-1-(6)_0–80 cm_B_R2_SC4	CSF21357	*C. hongkongensis*	AA--	L.L. Liu, J.L. Han & L.S. Sun	OK167582	OK168619	–	–

99	0–20 cm	20200816-1-(7)_0–20 cm_A_R1_SC1	CSF21358	*C. hongkongensis*	AA--	L.L. Liu, J.L. Han & L.S. Sun	OK167583	OK168620	–	–
99	0–20 cm	20200816-1-(7)_0–20 cm_A_R2_SC1	CSF21359	*C. hongkongensis*	BAAA	L.L. Liu, J.L. Han & L.S. Sun	OK167660	OK168697	OK169139	OK169223
99	0–20 cm	20200816-1-(7)_0–20 cm_A_R2_SC2	CSF21360	*C. hongkongensis*	BA--	L.L. Liu, J.L. Han & L.S. Sun	OK167661	OK168698	–	–
99	0–20 cm	20200816-1-(7)_0–20 cm_B_R1_SC1	CSF21361	*C. hongkongensis*	BA--	L.L. Liu, J.L. Han & L.S. Sun	OK167662	OK168699	–	–
99	0–20 cm	20200816-1-(7)_0–20 cm_B_R1_SC2	CSF21362	*C. hongkongensis*	BA--	L.L. Liu, J.L. Han & L.S. Sun	OK167663	OK168700	–	–
99	40–60 cm	20200816-1-(7)_0–60 cm_B_R2_SC1	CSF21363	*C. hongkongensis*	AA--	L.L. Liu, J.L. Han & L.S. Sun	OK167584	OK168621	–	–
99	40–60 cm	20200816-1-(7)_0–60 cm_B_R2_SC2	CSF21364	*C. hongkongensis*	AA--	L.L. Liu, J.L. Han & L.S. Sun	OK167585	OK168622	–	–
99	40–60 cm	20200816-1-(7)_0–60 cm_B_R2_SC3	CSF21365	*C. hongkongensis*	AA--	L.L. Liu, J.L. Han & L.S. Sun	OK167586	OK168623	–	–
99	40–60 cm	20200816-1-(7)_0–60 cm_B_R2_SC4	CSF21366	*C. hongkongensis*	AA--	L.L. Liu, J.L. Han & L.S. Sun	OK167587	OK168624	–	–
100	0–20 cm	20200816-1-(8)_0–20 cm_A_R1_SC1	CSF21367	*C. hongkongensis*	DAAA	L.L. Liu, J.L. Han & L.S. Sun	OK167699	OK168736	OK169147	OK169231
100	0–20 cm	20200816-1-(8)_0–20 cm_A_R1_SC2	CSF21368	*C. hongkongensis*	ACAB	L.L. Liu, J.L. Han & L.S. Sun	OK167599	OK168636	OK169116	OK169200
100	0–20 cm	20200816-1-(8)_0–20 cm_A_R2_SC2	CSF21370	*C. hongkongensis*	AAAA	L.L. Liu, J.L. Han & L.S. Sun	OK167588	OK168625	OK169112	OK169197
100	0–20 cm	20200816-1-(8)_0–20 cm_B_R1_SC1	CSF21371	*C. hongkongensis*	AA--	L.L. Liu, J.L. Han & L.S. Sun	OK167589	OK168626	–	–
100	0–20 cm	20200816-1-(8)_0–20 cm_B_R1_SC2	CSF21372	*C. hongkongensis*	ACAB	L.L. Liu, J.L. Han & L.S. Sun	OK167600	OK168637	OK169117	OK169201
100	0–20 cm	20200816-1-(8)_0–20 cm_B_R2_SC1	CSF21373	*C. hongkongensis*	AA--	L.L. Liu, J.L. Han & L.S. Sun	OK167590	OK168627	–	–
100	0–20 cm	20200816-1-(8)_0–20 cm_B_R2_SC2	CSF21374	*C. hongkongensis*	AA--	L.L. Liu, J.L. Han & L.S. Sun	OK167591	OK168628	–	–
100	80–100 cm	20200816-1-(8)_0–100 cm_A_R1_SC1	CSF21375	*C. hongkongensis*	AA--	L.L. Liu, J.L. Han & L.S. Sun	OK167592	OK168629	–	–

100	80–100 cm	20200816-1-(8)_0–100 cm_A_R1_SC2	CSF21376	*C. hongkongensis*	AA--	L.L. Liu, J.L. Han & L.S. Sun	OK167593	OK168630	–	–
100	80–100 cm	20200816-1-(8)_0–100 cm_A_R1_SC3	CSF21377	*C. hongkongensis*	AA--	L.L. Liu, J.L. Han & L.S. Sun	OK167594	OK168631	–	–
100	80–100 cm	20200816-1-(8)_0–100 cm_A_R1_SC4	CSF21378	*C. hongkongensis*	AA--	L.L. Liu, J.L. Han & L.S. Sun	OK167595	OK168632	–	–

[1] Number of 100 sampling points in this study. [2] Information associated with sample point and isolate, for example, "20200709-1-(1)_0–20 cm_A_R1_SC1" indicated sample number "20200709-1-(1), soil layer (0–20 cm), sample plastic bag (A), plastic sampling cup (R1), single conidium 1 (SC1). [3] CSF: Culture Collection located at China Eucalypt Research Centre (CERC), Chinese Academy of Forestry, ZhanJiang, GuangDong Province, China. [4] Genotype within each *Calonectria* species, determined by sequences of the *tef1*, *tub2*, *cmdA* and *his3* regions; "-" means not available. [5] *tef1* = translation elongation factor 1-alpha; *tub2* = β-tubulin; *cmdA* = calmodulin; *his3* = histone H3. [6] "–" represents the relative locus was not amplified in the current study.

Journal of Fungi

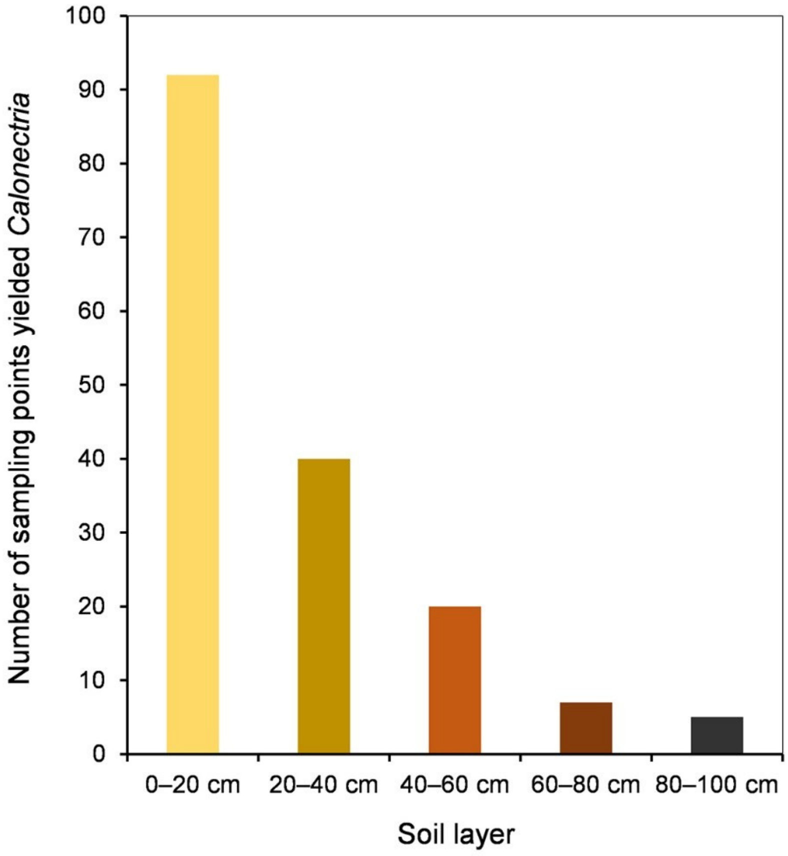

Figure S1. Number of sampling points that yielded *Calonectria* in each of five soil layers.

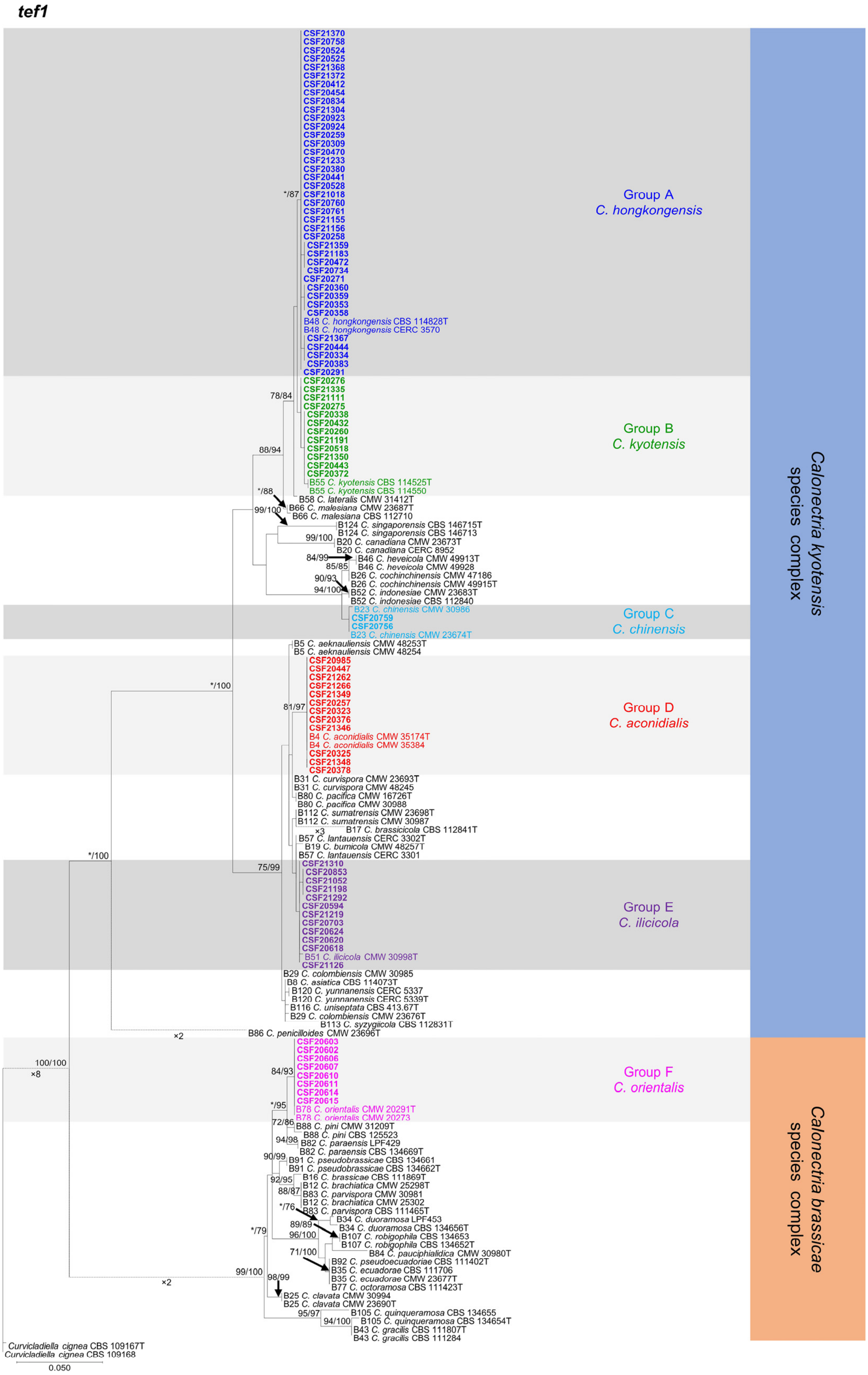

Figure S2. Phylogenetic tree of *Calonectria* species based on maximum likelihood (ML) analyses of the dataset of combined *tef1* gene sequences in this study. Bootstrap support values ≥70% are presented above the branches as follows: ML/MP. Bootstrap values <70% or absent are marked with "*". Isolates highlighted in six different colors and bold were obtained in this study. Ex-type isolates are marked with "T". The "B" species codes are consistent with the recently published results in Liu and co-authors [1]. The *Curvicladiella cignea* (CBS 109167 and CBS 109168) was used as outgroup taxon.

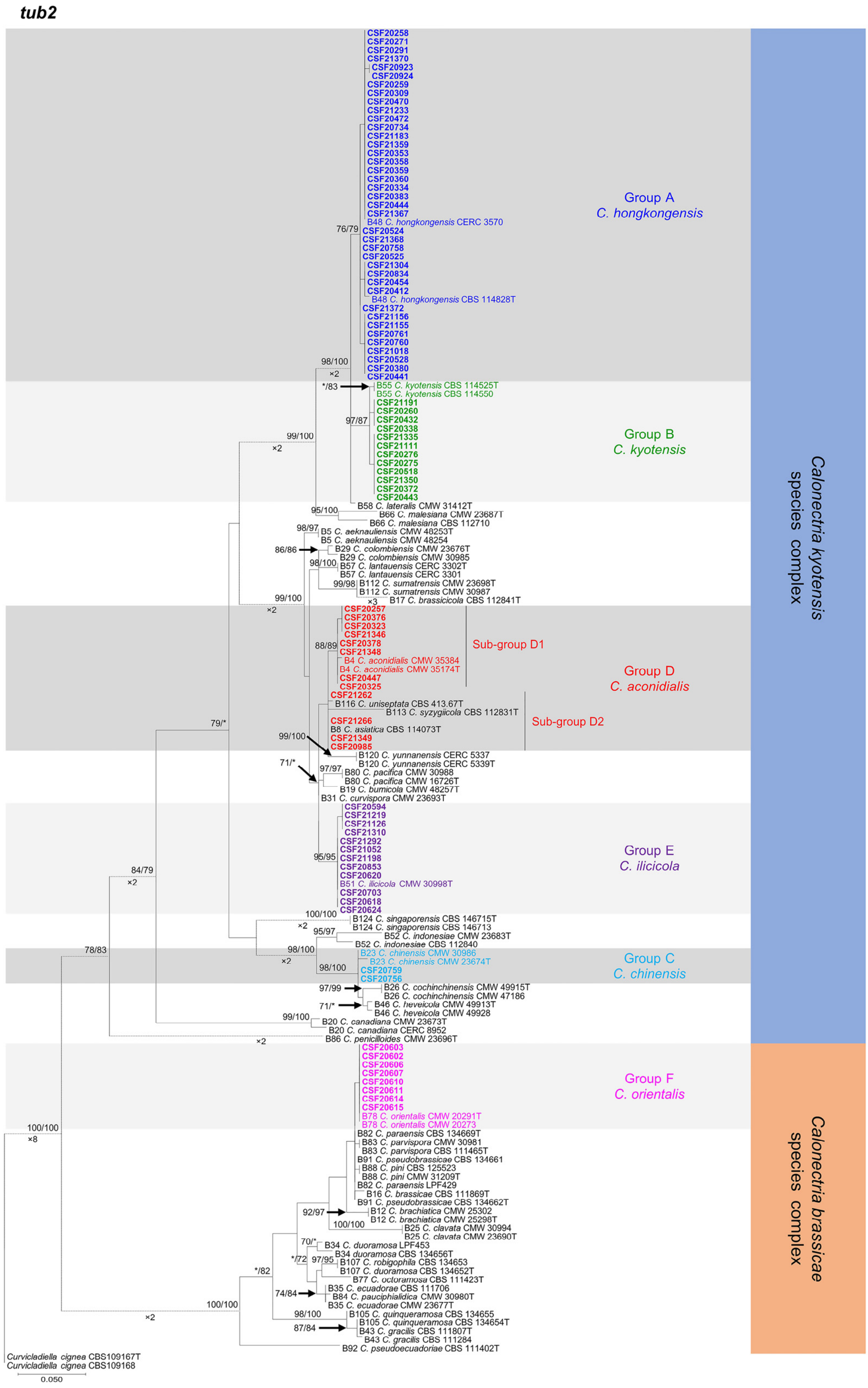

Figure S3. Phylogenetic tree of *Calonectria* species based on maximum likelihood (ML) analyses of the dataset of combined *tub2* gene sequences in this study. Bootstrap support values ≥70% are presented above the branches as follows: ML/MP. Bootstrap values <70% or absent are marked with "*". Isolates highlighted in six different colors and bold were obtained in this study. Ex-type isolates are marked with "T". The "B" species codes are consistent with the recently published results in Liu and co-authors [1]. The *Curvicladiella cignea* (CBS 109167 and CBS 109168) was used as outgroup taxon.

cmdA

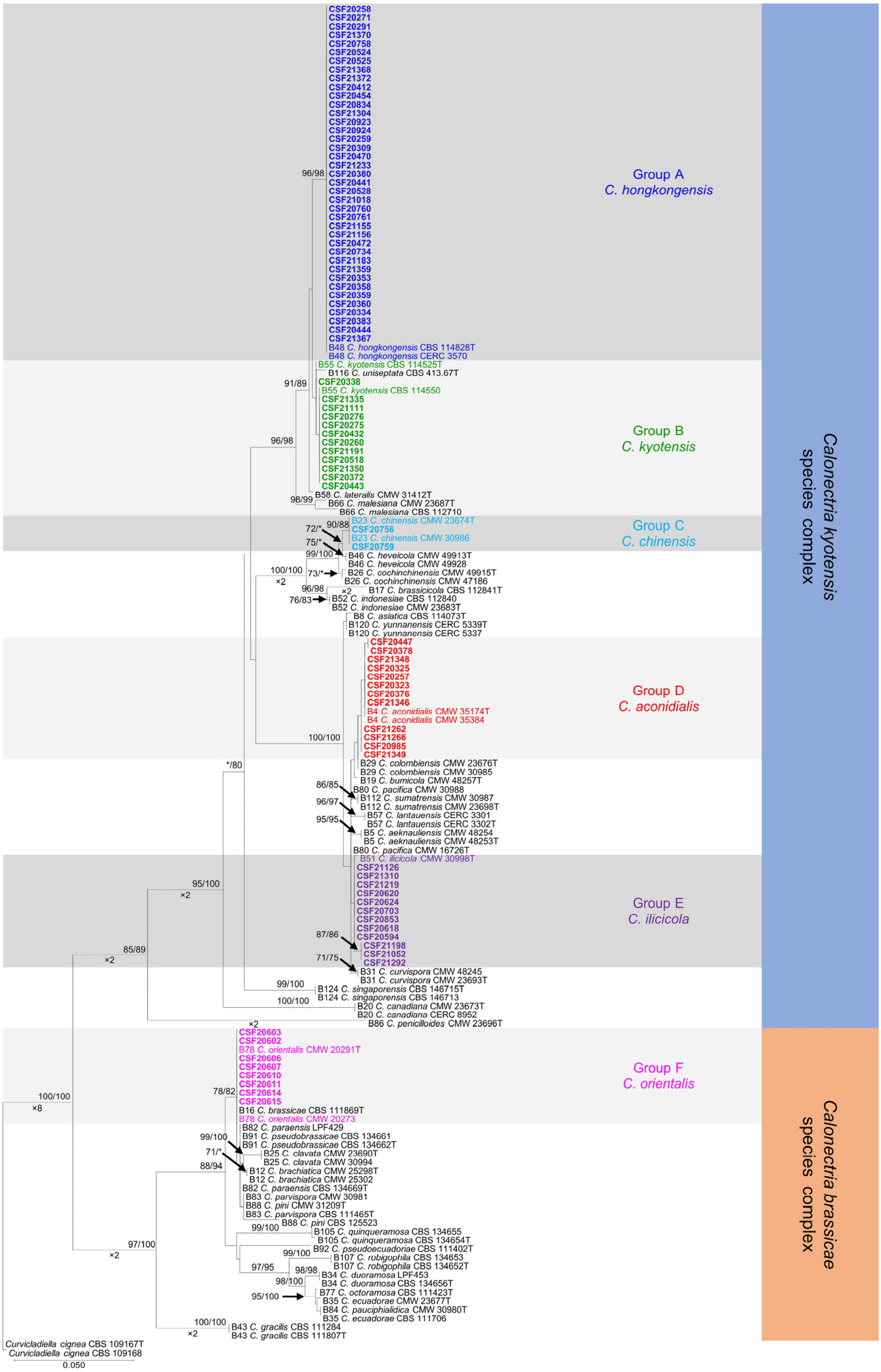

Figure S4. Phylogenetic tree of *Calonectria* species based on maximum likelihood (ML) analyses of the dataset of combined *cmdA* gene sequences in this study. Bootstrap support values ≥70% are presented above the branches as follows: ML/MP. Bootstrap values <70% or absent are marked with "*". Isolates highlighted in six different colors and bold were obtained in this study. Ex-type isolates are marked with "T". The "B" species codes are consistent with the recently published results in Liu and co-authors [1]. The *Curvicladiella cignea* (CBS 109167 and CBS 109168) was used as outgroup taxon.

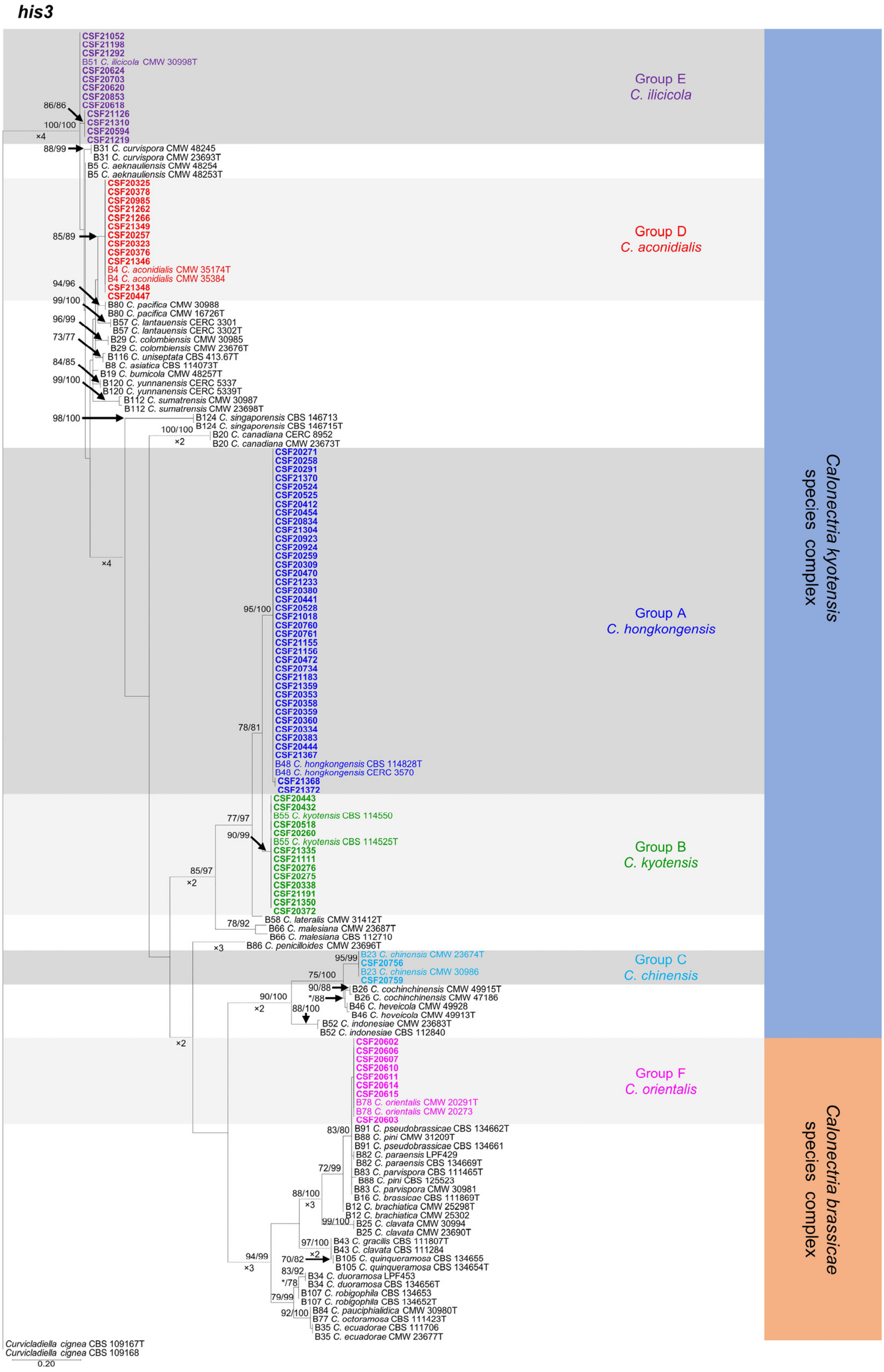

Figure S5. Phylogenetic tree of *Calonectria* species based on maximum likelihood (ML) analyses of the dataset of combined *his3* gene sequences in this study. Bootstrap support values ≥70% are presented above the branches as follows: ML/MP. Bootstrap values <70% or absent are marked with "*". Isolates highlighted in six different colors and bold were obtained in this study. Ex-type isolates are marked with "T". The "B" species codes are consistent with the recently published results in Liu and co-authors [1]. The *Curvicladiella cignea* (CBS 109167 and CBS 109168) was used as outgroup taxon.

References

1. Liu, Q.L.; Li, J.Q.; Wingfield, M.J.; Duong, T.A.; Wingfield, B.D.; Crous, P.W.; Chen, S.F. Reconsideration of species boundaries and proposed DNA barcodes for *Calonectria*. *Stud. Mycol.* **2020**, *97*, 100106, doi:10.1016/j.simyco.2020.08.001.
